# Achieving controllable packing mode and broad colour-tunable emission *via* the end group effect in pyrene-based aggregation-induced emission luminogens

**DOI:** 10.1039/d5sc03105b

**Published:** 2025-08-19

**Authors:** Chongyang Zeng, Shan Liang, Jieyu Lin, Wei Liu, Zhixin Xie, Wenxuan Cai, Carl Redshaw, Xing Feng, Ben Zhong Tang

**Affiliations:** a Guangdong Provincial Key Laboratory of Functional Soft Condensed Matter, School of Materials and Energy, Guangdong University of Technology Guangzhou 510006 P. R. China hyxhn@sina.com; b Chemistry, School of Natural Sciences, University of Hull Hull Yorkshire HU6 7RX UK; c School of Science and Engineering, Shenzhen Institute of Aggregate Science and Technology, The Chinese University of Hong Kong Shenzhen Guangdong 518172 P. R. China tangbenz@cuhk.edu.cn

## Abstract

To clarify the impact of the end groups on aggregation behaviour and emission properties, a series of α-cyanostilbene pyrene-based compounds (Py-R and Py-PR) were synthesized. These compounds display aggregation-induced emission characteristics with broad colour-tunable solid-state emission with the colour ranging from cyan to deep red (500–667 nm), achieved by modulating their end groups. The electronic effect of these end groups plays a key role in regulating the emission and the aggregation mode, demonstrating a strong correlation with Hammett constants. Specifically, as end groups transition from donor to acceptor substituents, the aggregates gradually shift from J-aggregation to H-aggregation. This transition is accompanied by a gradual red shift in emission wavelength and a decrease in quantum yield. This regulatory mechanism is realized by adjusting the electrostatic potential on the aromatic ring through the end groups, which in turn affects the centroid distance between adjacent π planes and intermolecular interactions in the aggregated state. Moreover, these AIEgens demonstrate remarkable photochromism through a continuous *Z* → *E* transformation and a [2 + 2] cycloaddition reaction. Leveraging these fascinating photophysical properties, selected green, yellow and red AIEgens have been successfully applied for anti-counterfeiting. This work demonstrates precise control over end-group effects on aggregation modes and emission behaviour, elucidates the underlying regulatory mechanism, and underscores its significance for tailoring advanced functional materials.

## Abstract 2

To clarify the impact of the end groups on aggregation behavior and emission properties, a series of α-cyanostilbene pyrene-based compounds (Py-R and Py-PR) were synthesized.

## Graphical abstract

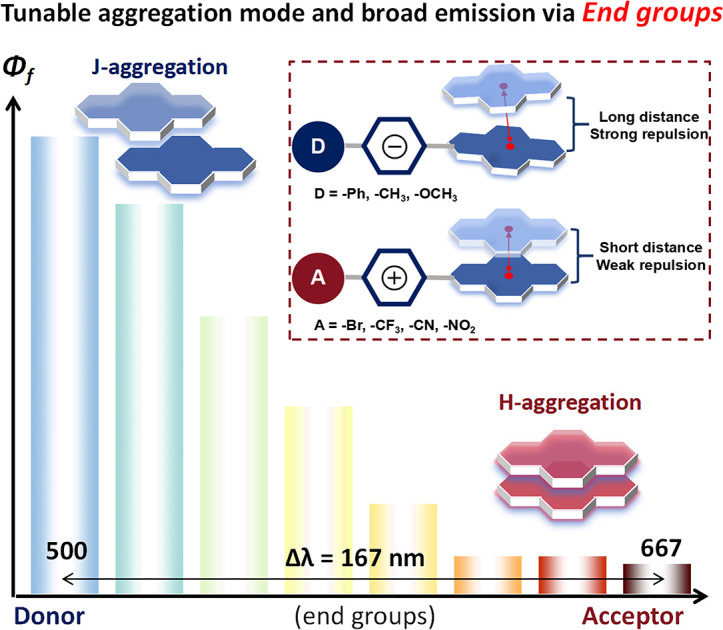

## Introduction

Aggregates, as a collection of molecules *via* interactions, often exhibit properties and functions that are quite different from their molecular units. Organic molecular aggregates have received extensive attention due to their unique aggregated optoelectronic properties, and simultaneously, their key advantages of tunable colour, low cost and easy fabrication have ensured their utility in various fields, such as organic light-emitting diodes (OLEDs), organic solid-state lasers (OSLs) and organic field effect transistors (OFETs).^1,2^ These photoelectric properties are mainly related to the influence of the aggregate structure and packing mode.^3^ For example, H-aggregation is beneficial to charge transport, and J-aggregation is profitable to fluorescence emission.^4^ Although many examples of H- or J-aggregates have been found and their relationship between aggregation modes and properties has been elaborated, it has proved difficult to achieve controllable aggregation modes by design at the single-molecule level.^5,6^ Therefore, it is necessary to find a systematic strategy that will enable pre-programming of molecular design so as to fine-tune the emission features through specific aggregation modes and thereby allow for the development of organic molecular aggregate materials with desirable optoelectronic properties.

End groups play an extremely important role in various fields of organic chemistry and are the smallest units of molecular or material design in the field of organic chemistry or materials science.^7–9^ Although these groups may only have a few differences, even at the atomic level, they may produce huge differences in properties and thus be applied to different fields.^10^ End groups with different electronic effects are widely used to study chemical reaction mechanisms; they can adjust photophysical and chemical processes and can induce inter/intra-molecular interactions, *etc.*^11,12^ For instance, classically, end groups can control the electrophilic substitution reaction at different sites by affecting the distribution of electrons on aromatic hydrocarbons.^13,14^ Furthermore, the end groups play a crucial role by influencing the electronic effect of the substituent to construct donor–acceptor (D–A) systems in luminescent molecules, thereby easily achieving controllable regulation of the emission to realize a multifunctional application.^15^ Unfortunately, the study of substituent effects is more limited to the molecular level, whilst systematic research on the aggregated states has largely been ignored, and importantly, molecules usually exist in the aggregated state in practical applications. Therefore, the realization of the controllable regulation of the substituent effect on the aggregation behaviour and the elaboration of its mechanism will provide valuable ideas for the design of materials in various fields.

Broad colour-tunable organic luminescent materials with aggregation-induced emission (AIE) properties are excellent candidates for potential applications in anti-counterfeiting, chemical sensors, *etc.*^16,17^ Various molecular strategies have been explored to construct colour-tunable luminescent materials, including intramolecular charge transfer (ICT),^18^ excited-state intramolecular proton transfer (ESIPT),^19^ Förster resonance energy transfer (FRET),^20^ and self-assembly.^21^ The preferred strategy involves creating a suitable D–A system by regulating the electronic effects of substituents through molecular engineering due to high controllability, stability and reproducibility.^22^ This approach allows for easier processing and regulation, enabling a wide emission colour range from blue to near-infrared red. Recently, a number of molecular engineering strategies have achieved broad-band emission by adjusting the conjugated backbone or π-bridge.^22–24^ Although these methods are effective, their synthesis is complicated, and the molecular properties are also significantly affected by the newly introduced parts. Therefore, it is meaningful to find easier and even atomic-level strategies to achieve broad colour-tunable emission.

Pyrene, a significant polycyclic aromatic hydrocarbon (PAH), has garnered considerable attention due to its high-efficiency blue photoluminescence and excellent carrier mobility.^25^ It is a candidate unit for studying aggregation behaviour because of its special large π-conjugated structure and high-resolution monomer and dimer emission. Taking pyrene as the research object is more convenient to observe and understand the behaviour of aggregates and then reveal the structure–property relationships. However, due to strong π–π stacking in the aggregated state, the aggregation-caused quenching (ACQ) effect of pyrene-based molecules is very unfavorable for the investigation of fluorescence and even practical applications.^26^ The discovery of AIE luminogens (AIEgens) has provided a solution to overcome ACQ, achieving high-contrast emission changes.^27,28^ α-Cyanostilbenes with a twisted structure are among the important units to endow dyes with AIE characteristics, and they also possess multiple functionality (such as solvatochromism, photochromism, mechanochromism, *etc.*).^29–31^ Furthermore, there is limited research on systematically developing α-cyanostilbene pyrene-based AIEgens with broad colour-tunable emission from blue to red, and the structure–property relationships also remain unclear.

In this study, a series of α-cyanostilbene pyrene-based molecules were synthesized *via* a Knoevenagel reaction characterized by end group modification by different electronic effects. They exhibit remarkable AIE characteristics, with broad colour-tunable emission from cyan to red in the aggregated state, through the precise regulation of the electronic effect of the end groups, ranging from electron-donating (–CH_3_) to electron-withdrawing (–NO_2_) groups. Furthermore, the electronic effect of the end groups on the aggregation modes and emission behaviour was systematically examined. Additionally, these molecules display fascinating photochromism attributed to successive *Z* → *E* isomerization and [2 + 2] cycloaddition reactions, and the mechanisms were elucidated through a combination of experimental and theoretical studies. Moreover, these AIEgens were successfully utilized as colourful inks for fluorescent anti-counterfeiting applications, achieving a vivid “*Burn after Reading*” effect. This work provides valuable insight into how the electronic effect of the end groups influences the aggregation modes and emission behaviour. It also highlights their potential for multi-colour emission in anti-counterfeiting applications.

## Results and discussion

### Synthesis and characterization

**Fig. 1:**
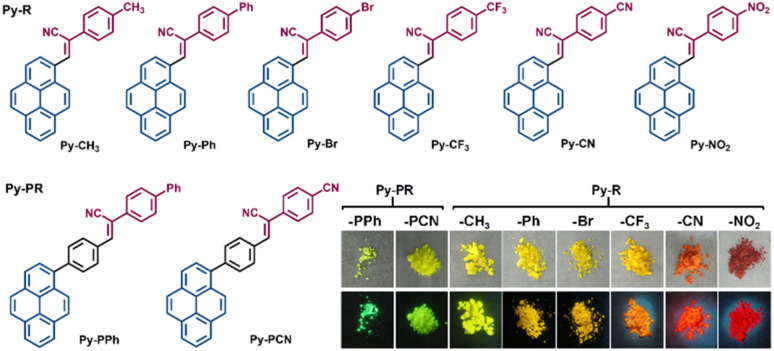
The molecular structures of the α-cyanostilbene pyrene-based molecules Py-R and Py-PR are illustrated. Insets show photographs of compounds Py-R and Py-PR under daylight (top) and UV irradiation (bottom).

In order to study the effect of end groups on the optical behaviour in solution and in the aggregated state, a family of α-cyanostilbene pyrene-based molecules (Py-R) modified with different electronic effect substituents was designed and synthesized, as shown in Scheme S1 (the methoxy modified compound Py-OMe was referred to in previous reports),^32^ and the corresponding molecular structures are summarized in Fig. 1. The Knoevenagel condensation reaction of pyrene-1-carbaldehyde with the corresponding phenylacetonitrile derivatives afforded the target compounds Py-R (Py-CH_3_, Py-Ph, Py-Br, Py-CF_3_, Py-CN and Py-NO_2_) in high yield (*ca.* 70%). As the electronic effect from the donor (D) to the acceptor (A) followed the order of –CH_3_, –Ph, –Br, –CF_3_, –CN, –NO_2_, the apparent colour of the target compounds gradually changed from green to red. To further investigate the effect of the extended π-bridge on photophysical behaviour, 4-(pyren-1-yl)benzaldehyde was mixed with either 2-([1,1′-biphenyl]-4-yl)acetonitrile or 4′-(cyanomethyl)-[1,1′-biphenyl]-4-carbonitrile to produce the final compounds Py-PR (Py-PPh and Py-PCN). All compounds show good solubility in common solvents, such as CH_2_Cl_2_, THF, DMSO, *etc.*, but are insoluble in water and ethanol*.* These obtained pyrene-based cyanostilbene compounds Py-R and Py-PR were characterized by ^1^H/^13^C/^19^F NMR spectroscopy, high-resolution mass spectrometry (HRMS), and single-crystal X-ray diffraction analysis (Fig. S1–S24).

### Photophysical properties

**Fig. 2:**
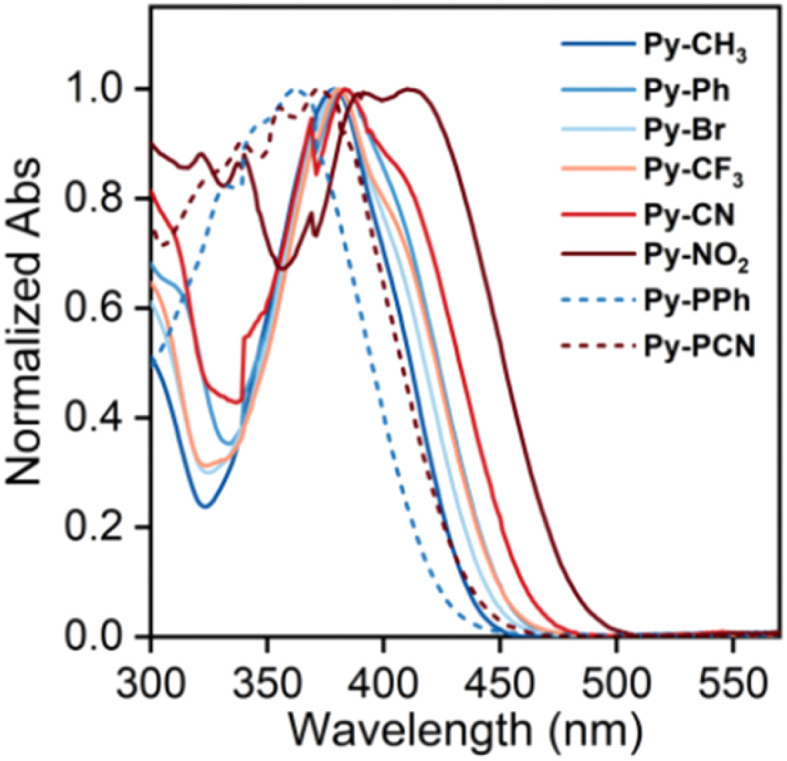
UV-vis absorption spectra of Py-R and Py-PR in THF (10^−5^ M).

To investigate the impact of electronic effects of end groups on the photophysical properties of the α-cyanostilbene pyrene-based molecules, UV-vis absorption spectra were measured in dilute tetrahydrofuran (THF, 10^−5^ M). As shown in Fig. 2, the Py-R molecules exhibit characteristic absorption spectra with a broad absorption band (*λ*_abs_) in the range of 378–410 nm, accompanied by an additional shoulder band near 310 nm (Table 1). As the end-group electronic effect shifts from D to A, the maximum absorption band is red-shifted by *ca.* 32 nm, which is attributed to the intramolecular charge transfer (ICT) effect. The high-energy absorption bands correspond to the H-2 → L transition (40–73%) with a lower oscillator strength (*f*_osc_) of 0.1996 to 0.3025, and the low-energy bands correspond to the dominant H → L transition (∼98%) with a higher *f*_osc_ of 0.6259 to 1.1309 (Table S3). In contrast, the dominant absorption bands of Py-PR molecules are blue-shifted to 358 nm for Py-PPh and 366 nm for Py-PCN, which can be attributed to the presence of a π-bridge (phenyl) between the pyrene and α-cyanostilbene units, twisting the molecular conformation.

**Table 1:** The linear photophysical parameters of Py-R and Py-PR

Comps.	*λ*_max abs_ a [nm]/*ε* [M^−1^ cm^−1^]	*λ*_em_ (nm)^b^			*Ф*_f_			*τ* (ns)		*α*_AIE_ c	CIE1931 (*x*, *y*)^d^
		*f*_w_		Solid	*f*_w_		Solid	THF	Solid		
		0^c^	99%		0	99%					
Py-PPh	358/8.7 × 10^4^	498	525	500	60%	76%	61%	1.64	1.77	1.0	(0.19, 0.56)
Py-PCN	366/2.4 × 10^4^	538	560	520	46%	68%	52%	1.12	2.16	1.1	(0.28, 0.60)
Py-CH_3_	378/3.6 × 10^4^	467	530	525	1%	40%	37%	1.03	3.79	37.0	(0.33, 0.59)
Py-Ph	382/3.1 × 10^4^	482	558	553	1%	26%	25%	0.67	4.13	26.0	(0.42, 0.54)
Py-Br	380/2.9 × 10^4^	476	565	556	1%	16%	12%	1.15	4.90	12.0	(0.43, 0.55)
Py-CF_3_	382/7.0 × 10^4^	488	656	572	1%	5%	5%	0.89	5.21	5.0	(0.48, 0.48)
Py-CN	384/1.7 × 10^4^	500	636	630	1%	4%	5%	1.15	5.69	5.0	(0.64, 0.36)
Py-NO_2_	410/1.2 × 10^4^	538	684	667	1%	2%	4%	1.91	5.92	4.0	(0.70, 0.30)

a Maximum absorption peak in THF solution.

b Maximum emission peak.

c *α*_AIE_ = *Ф*_f solid/_*Ф*_f THF_.

d In the solid state.

### AIE characteristics and aggregation behaviour

**Fig. 3:**
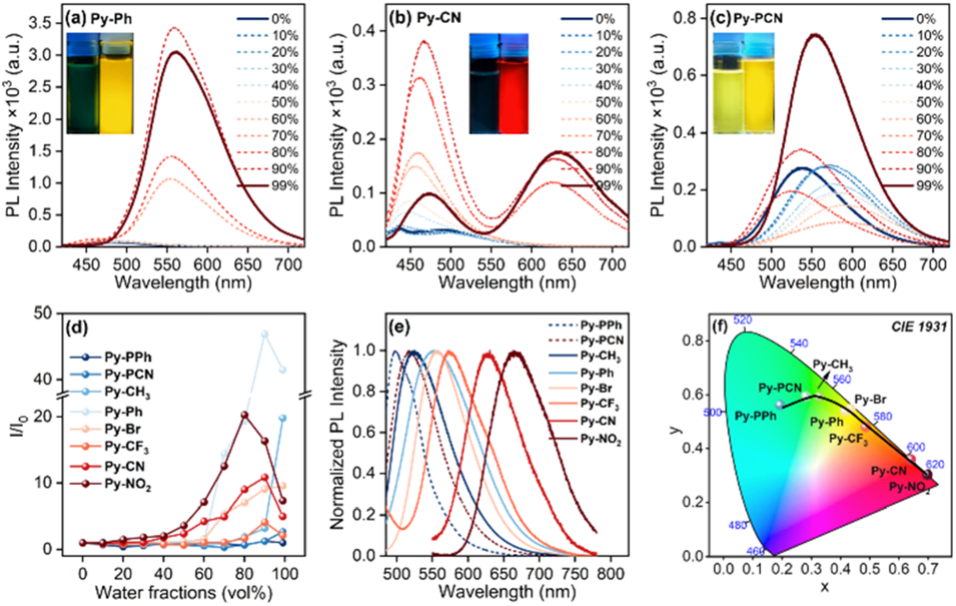
Emission spectra of (a) Py-Ph, (b) Py-CN and (c) Py-PCN in THF/H_2_O with varying *f*_w_; (d) *I*/*I*_0_ curves of Py-R and Py-PR as a function of *f*_w_; (e) emission spectra and (f) CIE1931 color coordinates of Py-R and Py-PR in the solid state.

The α-cyanostilbene unit is widely recognized as a classical substituent for facilitating the ACQ-to-AIE transformation, and chromophores incorporating the cyanostilbene scaffold typically exhibit pronounced AIE characteristics.^33^ The AIE properties of Py-R and Py-PR were investigated in THF/H_2_O mixtures with various water fractions (*f*_w_, vol%) (Fig. 3 and S26–S31). Both Py-CH_3_ and Py-Ph exhibit weak cyan emissions in THF, with maximum emission peaks (*λ*_em_) at 467 and 482 nm. Upon increasing the *f*_w_ from 0 to 99%, the emission intensity was enhanced by 25- and 40-fold with red-shifted emission to 560 and 530 nm, accompanied by strongly enhanced fluorescence quantum yields (*Ф*_f_) of 40% for Py-CH_3_ and 25% for Py-Ph, respectively (Fig. 3a and S27). These results confirm that both Py-CH_3_ and Py-Ph are AIE-active molecules.

In contrast to Py-CH_3_ and Py-Ph, the electron-withdrawing group decorated α-cyanostilbene pyrene-based compounds Py-Br, Py-CF_3_, Py-CN, and Py-NO_2_ exhibit a dual emission with distinct characteristics (Fig. 3b and S28–S30). For example, Py-CN displays weak dual emission bands at approximately 500 and 630 nm (Fig. 3b) with *Ф*_f_ of *ca.* 1%. When *f*_w_ is below 80%, the intensity of the short-wavelength emission was gradually enhanced with a slight redshift. When *f*_w_ = 80%, the intensity at *λ*_em_ = 430 nm is promoted approximately 11-fold, and a new emission peak emerges at 625 nm. Subsequently, the intensity of the short-wavelength emission decreases, accompanied by an increased emission in the long-wavelength emission region, resulting in orange-red fluorescence with a weakly enhanced *Ф*_f_ of 4%. Similarly, Py-Br, Py-CF_3_, and Py-NO_2_ also exhibit dual-emission enhancement properties in the aggregated state, with weakly enhanced *Ф*_f_ values of 16%, 5%, and 2%, respectively, reflecting a weak AIE effect. In addition, the short-wavelength and long-wavelength emissions are assigned to the monomer emission and the excimer emission, respectively.

Unlike Py-R, Py-PPh and Py-PCN exhibit bright emission in THF, with *λ*_em_ at 500 and 520 nm, and relatively high *Ф*_f_ (60% for Py-PPh and 46% for Py-PCN, respectively) (Fig. 3c and S31). As the *f*_w_ increases to 70%, their emission intensity gradually decreases, accompanied by a red-shifted *λ*_em_ to 530 nm for Py-PPh and 580 nm for Py-PCN, which is attributed to the ICT effect. Upon further aggregation, the emission intensity rapidly enhances, with a slightly blue-shifted emission to 525 nm for Py-PPh and 560 nm for Py-PCN, and increased *Ф*_f_ (76% for Py-PPh and 68% for Py-PCN) (Fig. 3d), clearly reflecting aggregation-enhanced emission (AEE) properties. Moreover, the initial red shift is attributed to the ICT effect, while the subsequent blue shift in higher *f*_w_ is due to the dominance of the hydrophobic aggregates overwhelming the ICT effect.^34^

Furthermore, the solvatochromic effects of Py-R and Py-PR were investigated in various solvents (Fig. S32–S39). Although the absorption spectra of these α-cyanostilbene pyrene-based compounds exhibit slight red shifts, the emission spectra show a significant change. As the solvent polarity increased from cyclohexane to DMSO, the long-wavelength emission bands of Py-CH_3_, Py-Ph, Py-Br, Py-CF_3_ and Py-CN were slightly red-shifted by no more than 50 nm, showing the limited ICT effect, but the strongest electron-withdrawing decorated Py-NO_2_ exhibits a pronounced ICT effect with an enormous red-shift to 605 nm. Additionally, D-π-A compounds containing π-bridges, such as Py-PPh and Py-PCN, display distinct solvent-dependent emission with red-shifts of 110 and 160 nm, respectively, showing a much stronger ICT effect (Fig. S38 and S39). These results further confirm that the electronic nature of the end groups can significantly regulate the impact of the solvent effects on the emission properties.

The excited state dynamics of Py-R and Py-PR were further studied by time-resolved emission spectroscopy (Fig. S40–S48). In different solvents, their fluorescence lifetimes show obvious end-group dependence: as the end groups change from electron-donating –CH_3_ to electron-withdrawing –NO_2_, the lifetime of Py-R decreases gradually from 1.14 to 0.57 ns in weakly polar CHX, while increases from 0.70 to 5.92 ns in highly polar DMSO. This is mainly due to the significant difference in the molecular dipole moment caused by the end groups. For the electron-donating modified Py-R (R = –CH_3_, –Ph), their dipole moment is smaller, and they can be stabilized by less polar solvents (*e.g.* CHX, toluene, *etc.*), leading to a longer lifetime, while for the electron-withdrawing modified Py-R (R = –Br, –CF_3_, –CN, –NO_2_), their dipole moment is increased, so they are stabilized in large polar solvents (*e.g.* DMF, DMSO, *etc.*) to extend the lifetime. A similar trend is also applicable in Py-PR, which is reflected in the lifetimes of Py-PPh and Py-PCN, which are 0.66 and 0.39 ns in CHX, while they are 2.32 and 2.59 ns in DMSO, respectively. In the solid state, the lifetime gradually increases from 3.79 to 5.92 ns as the end groups change from –CH_3_ to –NO_2_, which is mainly because the electron-withdrawing group enhances the strong intermolecular interactions such as π–π stacking.

More importantly, these compounds display a wide range of tunable emission from cyan to deep red, with *λ*_em_ spanning from 500 to 667 nm in the solid state (Fig. 3e and f). The emission spectra of Py-PPh and Py-PCN in the solid state closely resemble those in solution, suggesting that the dominant emission primarily originates from the charge transfer process. The presence of the π-bridge (phenyl) effectively suppresses any π–π stacking in the aggregated state, a finding corroborated by the concentration-dependent emission spectra of Py-PR (from 10^−5^ to 2 × 10^−3^ M) (Fig. S49–S56). In contrast, the maximum emission peaks (*λ*_em_) in the solid state for Py-R have dramatically red-shifted by about 58–130 nm, accompanied by a decreased *Ф*_f_ (37% to 4%) in the order of Py-CH_3_ > Py-Ph > Py-Br > Py-CF_3_ > Py-CN > Py-NO_2_, compared to in THF solution. This may be ascribed to the synergistic effect of the weak ICT effect and stronger intermolecular interactions, resulting in a pronounced, red-shifted emission and low *Ф*_f_. It is worth noting that under the similar influence of a limited ICT effect, the electron-withdrawing end-group modified Py-R (R = –NO_2_, –CN, –CF_3_, –Br) show a somewhat lower *α*_AIE_ (4–12) while the electron-donating end-group modified Py-R (R = –Ph, –CH_3_, –OMe) shows higher *α*_AIE_ (26 for Py-Ph, 37 for Py-CH_3_, and 50 for Py-OMe), which means that the *λ*_em_, *α*_AIE_ and *Ф*_f_ of the step-by-step change are mainly related to the aggregation behaviour, and show significant end-group dependent photophysical properties.

The intramolecular motion mechanism was further studied *via* the viscosity-dependent emission in mixtures of DMSO/glycerol with different glycerol fractions (Fig. S57–S64). For Py-R (except Py-NO_2_), with the increase of glycerol fraction, the emission intensity increases about 6-fold with a smaller change of *λ*_em_, due to the restriction of intramolecular motions in the high viscosity of glycerol. As the glycerol fractions increase, the emission behaviour of Py-NO_2_ was changed from dual emission peaks to single emission at 470 nm with enhanced emission intensity, suggesting that π–π stacking has been suppressed. In contrast, the fluorescence intensity of Py-PR changes slightly with the increase of viscosity (about 1.8-fold), indicating that Py-PR has a weaker free motion and structural relaxation, which is consistent with the higher *Φ*_f_ of Py-PR in dilute solution.

**Fig. 4:**
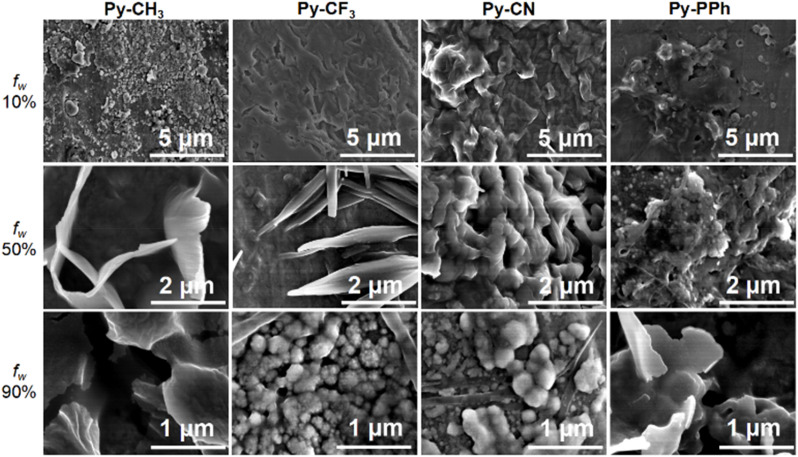
SEM photographs of Py-CH_3_, Py-CF_3_, Py-CN and Py-PPh in a solution of THF/H_2_O with *f*_w_ = 10%, 50% and 90%.

The morphology of aggregates of typical Py-CH_3_, Py-CF_3_, Py-CN and Py-PPh samples in THF/H_2_O with various *f*_w_ was further studied by field emission scanning electron microscopy (FE-SEM) (Fig. 4). When *f*_w_ = 10%, the compounds were dispersed in the field of view without a characteristic aggregation structure. As the *f*_w_ increased to 50%, segmental Py-CH_3_ and Py-CF_3_ initially self-assembled into a twisted linear morphology with a width of about 300 nm, Py-CN aggregated into a sponge-like structure, while Py-PPh showed an aggregation trend without a characteristic aggregation structure at this time. In *f*_w_ = 90%, Py-CH_3_ and Py-PPh formed lamellar aggregates, while Py-CF_3_ and Py-CN formed dense spherical aggregates with an average particle size of about 150 nm (Fig. S65 and S66), indicating that the electron-withdrawing group promotes the formation of spherical aggregates, while the electron-donating group is conducive to lamellar aggregates in high *f*_w_ solutions. These results provide visual evidence for the formation of aggregates.

**Fig. 5:**
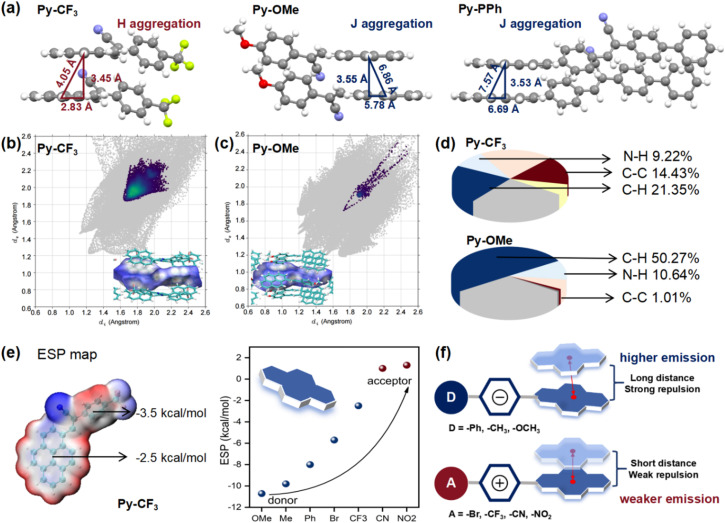
(a) Single crystals and packing modes in dimers of Py-CF_3_, Py-OMe, and Py-PPh; Hirshfeld surfaces and decomposed fingerprint plots of intermolecular C–C interaction of (b) Py-CF_3_ and (c) Py-OMe; (d) the contribution of various atoms in intermolecular interactions; (e) the molecular ESP of Py-CF_3_ and the variation of the ESP on pyrene with the end groups; (f) schematic illustration of aggregation mode influenced by the electronic effect of end groups.

Furthermore, single crystal X-ray diffraction was performed to further understand the electronic effect of the end groups on the molecular packing in the aggregated state (Fig. 5a and S67). Single crystals of Py-CF_3_ (CCDC: 2433820) and Py-PPh (CCDC: 2433821) suitable for X-ray diffraction analysis were obtained from chloroform solutions *via* slow evaporation at room temperature. For comparison, the single crystal structure of Py-PPh (CCDC: 1529865) is also presented. All single crystals exhibit a non-planar *Z*-conformation and form dimers *via* a head-to-head packing mode in aggregation. In Py-CF_3_, Py-OMe and Py-PPh packing modes, the slip stacking of pyrene units between adjacent molecules was observed, with slip distances of 2.83, 5.78 and 6.69 Å and the centroid-to-centroid distances of 4.05, 6.86 and 7.57 Å, respectively. The calculated slip angles for Py-CF_3_, Py-OMe and Py-PPh are 58.5°, 20.8° and 27.8°, respectively; thus, the aggregation pattern of Py-CF_3_ is classified as H-aggregation (slip angle >54.7°), while Py-OMe and Py-PPh are J-aggregation.^35^ Therefore, the electron-withdrawing end groups (such as –NO_2_, –CN, –CF_3_, and –Br) readily induce H-aggregation, which is favourable for charge carrier migration properties, while the electron-donating end groups (such as –Ph, –CH_3_, and –OMe) are more inclined to J-aggregation, which is favourable for luminescent properties. This is consistent with the lower *Ф*_f_ and more enormous red-shifts of the electron-withdrawing Py-R but higher *Ф*_f_ and smaller red-shifts of electron-donating Py-R in the solid state.

To further investigate how the electronic effect of the end group influences the intermolecular interaction between α-cyanostilbene pyrene-based compounds in the aggregated state, Hirshfeld surface analysis was conducted based on the single-crystal structures (Fig. 5b, c and S69).^36,37^ The intermolecular C–C interactions (shaded in the fingerprint graph) usually lead to π–π stacking and long-wavelength emission of dimers, accounting for a higher proportion of 14.43% in all intermolecular interactions of Py-CF_3_, but much lower in Py-OMe and Py-PPh (1.01% and 7.08%). Among them, the π–π stacking in the Py-CF_3_ aggregates is clearly shown in the white highlighted area in the Hirshfeld surface map and is mainly concentrated at the pyrene group and the phenyl moiety. This supports the large-wavelength emission and lower *Φ*_f_ in the aggregated state of Py-R modified by electron-withdrawing groups (–NO_2_, –CN, –CF_3_, and –Br). In addition, about 9% of the N–H interactions in all molecules are attributed to the hydrogen bonds between the cyano group and adjacent molecules. On the other hand, the proportion of weak C–H interactions in Py-OMe and Py-PPh is as high as 50.27% and 41.19%, while that of Py-CF_3_ is only 21.35%, indicating that the electron-donating group-modified Py-R mainly stabilizes the aggregation geometry and produces higher *Φ*_f_*via* weak C–H⋯π interactions and C–H⋯N hydrogen bonds (Fig. 5d and Table S2).

Moreover, molecular electrostatic surface potential (ESP) maps were calculated to further understand the nature of the influence of the electronic effect of the end groups on the aggregation behaviour (Fig. 5e and S70).^38^ The ESP energy of the pyrene unit shows a significant substituent dependence, that is, as the end groups change from the donor to the acceptor, the ESP energy of the minimum point becomes more and more positive (from −10.7 to 2.0 kcal mol^−1^). The larger negative ESP level on the surface of polycyclic aromatic hydrocarbons produces a stronger repulsion between adjacent molecules and prolongs the distance between the two π-planes thereby inhibiting π–π stacking and enhancing emission (Fig. 5f). In addition, according to the distribution of the frontier molecular orbital electron cloud (Fig. S71), in the dimer of Py-CF_3_, the shorter intermolecular distance promotes the accumulation of effective electron π conjugation and the charge transfer between adjacent molecules, so it has a larger red-shift *λ*_em_ in aggregation, which is beneficial to charge transport; while in the dimer of Py-OMe, the longer distance limits the accumulation of electrons between molecules, so the red shift of the *λ*_em_ in the aggregated state is limited. Therefore, the end groups can adjust the electrons on the polycyclic aromatic hydrocarbons through the electronic effect to achieve controllable aggregation mode and AIE characteristics.

### DFT calculations

**Fig. 6:**
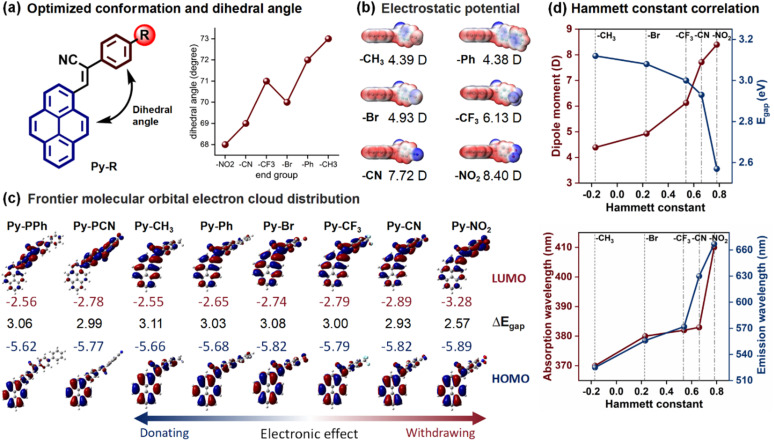
(a) Schematic illustration of the dihedral angle of Py-R influenced by the end groups; (b) molecular electrostatic surface potential energy diagrams and dipole moment values of Py-R; (c) frontier molecular orbital electron cloud distributions and energy gaps of Py-R and Py-PR; (d) the relationship of the Hammett constant and photophysical parameters of Py-R with different end groups.

The end group effects on the photophysical properties at the single-molecule level were interpreted by density functional theory (DFT) calculations performed using Gaussian 09 at the B3LYP/6-31g+(d,p) basis set (Fig. 6). First, the optimized molecular structures and electronic distributions of Py-R and Py-PR were analyzed. As shown in Table S3, all Py-R exhibit a distorted molecular conformation, with the dihedral angle between the pyrene and α-cyanostilbene units increasing from 68° to 73° (Fig. 6a) as the electronic effect of the end group changes from D to A, accompanied by a decreased molecular dipole moment from 4.38 to 8.40 D (Fig. 6b) indicating that the electronic nature of the end groups significantly influences the molecular conformation and dipole moment, resulting in distinct optical properties.

The electron distribution of the frontier molecular orbitals further illustrates this effect (Fig. 6c). In the Py-R system, the highest occupied molecular orbital (HOMO) and the lowest unoccupied molecular orbital (LUMO) of Py-CH_3_ are primarily distributed across the entire molecule, showing a weak ICT effect. As the electronic effect of the end group shifts from the donor to the acceptor, the electron in the HOMO gradually localizes on the pyrene, while in the LUMO it shifts towards the α-cyanostilbene unit, which reflects an enhanced ICT effect from Py-CH_3_ to Py-NO_2_. Although the electron-withdrawing effects promote the enhanced ICT effect, the limited π-conjugated structure of Py-R makes the ICT very limited. In contrast to Py-PR with an extended π-conjugated structure, the electron density in the HOMO is mainly located on the pyrene and the π-bridge (phenyl), while in the LUMO, the electron density of Py-PPh is spread across the pyrene ring and α-cyanostilbene unit, and that of Py-PCN is centered on the α-cyanostilbene unit. This separation of the HOMO and LUMO enhances the ICT process, consistent with the observed solvatochromism. The calculated band gaps (Δ*E*_gap_) decrease from 3.11 to 2.57 eV in the Py-R system and from 3.03 to 2.99 eV in the Py-PR system as the end group changes from electron-donating to electron-withdrawing. This trend aligns with the end group's role in tuning the absorption and emission spectra across both Py-R and Py-PR systems, which is in good agreement with the experimental results. Moreover, the results show that both the theoretical results (dihedral angle, dipole moment and Δ*E*_gap_) and spectral experiments (UV-vis absorption and emission wavelength) are correlated with the Hammett constant (Fig. 6d), which provides a foundation for the rational design of broad colour-tunable fluorescent materials.

### Photochromism

**Fig. 7:**
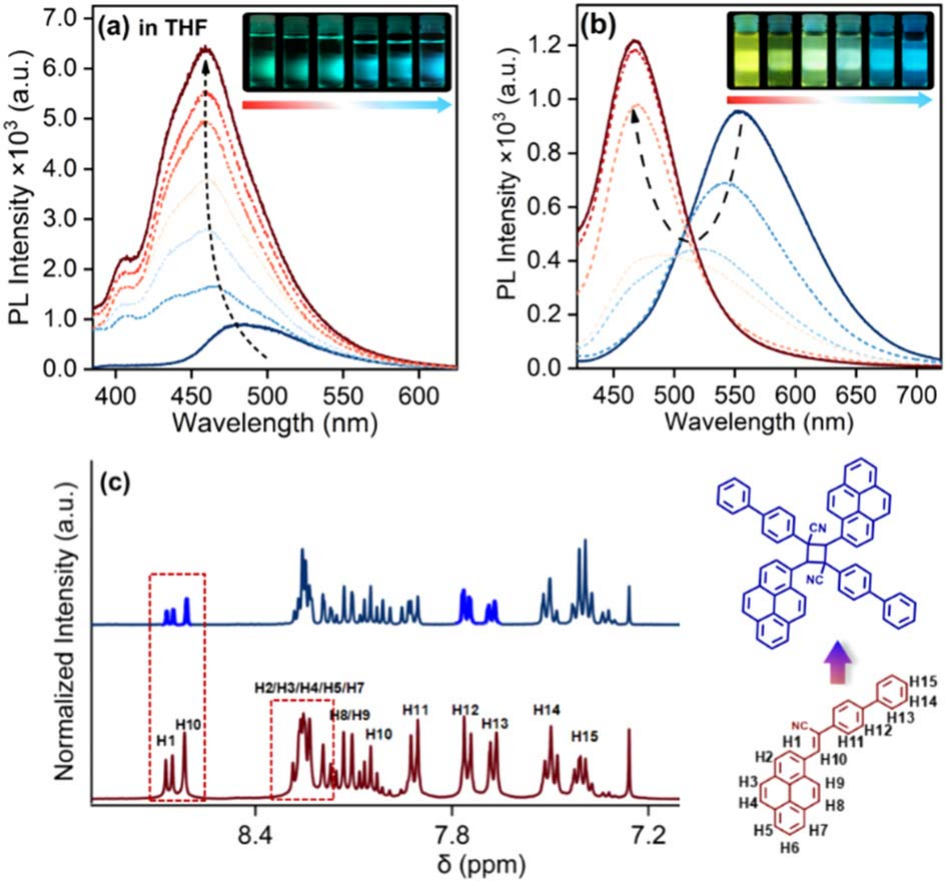
Emission spectra of Py-Ph (a) in THF (10^−5^ M) and (b) in THF/H_2_O with *f*_w_ = 90%; (c) ^1^H NMR spectrum of Py-Ph before and after irradiation.

Inspired by our previous work, the photo-induced transformations of α-cyanostilbene pyrene-based molecules have also been explored.^39^ The α-cyanostilbene pyrene-based compounds Py-R and Py-PR display intriguing photo-responsive behaviour both in solution and aggregated states upon irradiation. For instance, in THF (10^−5^ M), the *λ*_em_ of Py-Ph is gradually blue-shifted from 480 to 460 nm with a nearly 7-fold enhancement in emission intensity, after being irradiated with a 30 W UV lamp for approximately 3 h (Fig. 7a). More remarkably, in the aggregated state (*f*_w_ = 90%), the emission intensity of Py-Ph undergoes a significant blue-shift from 552 to 466 nm (Fig. 7b). Meanwhile, the emission intensity initially decreases and then increases, and the emission colour changes sequentially from yellow to warm white and finally to blue under the same experimental conditions. Similarly, all Py-R compounds exhibit analogous photochromism to that of Py-Ph (Fig. S72–S79).

Further evidence was provided by NMR spectroscopy and high-resolution mass spectrometry (HRMS) (Fig. 7c and S25). The ^1^H NMR spectra of Py-Ph clearly shows six pairs of double peaks at *δ* 8.66, 8.14, 8.10, 7.92, 7.75, and 7.67 ppm, three triplet peaks at 8.06, 7.50 and 7.41 ppm and a singlet peak at 8.62 ppm with integration rate of 1 : 1 : 1 : 2 : 2 : 2 : 1 : 2 : 1 : 1, which corresponds to the H1, H8, H9, H11, H12, H13, H6, H14, H15 and H10, respectively. The other proton peaks in the range of 8.29–8.17 ppm correspond to the H2, H3, H4, H5 and H7. After UV irradiation for 3 h, the characteristic proton peaks at 8.66 and 8.61 ppm remain, indicating the presence of Py-Ph. However, new triplet and the singlet proton peaks emerge at 7.34 ppm and 6.91 ppm, suggesting the formation of a new product.^40,41^ Based on the proton NMR integration area, the ratio of Py-Ph to the new compound is determined to be 1 : 1.8. Thus, it is inferred that Py-Ph undergoes an intermolecular [2 + 2] cycloaddition to achieve 2@Py-Ph. Additional HRMS analysis shows that the molecular peak (*m*/*z* + Na^+^) of Py-Ph is 428.1409. After 3 h of irradiation, the mass-to-charge ratio (*m*/*z* + Na^+^) changed to 833.2916, indicating the formation of a new cycloaddition product, 2@Py-Ph (Fig. S25). Thus, these results offer detailed evidence demonstrating the photo-induced cycloaddition reaction of Py-Ph to form the new cycloaddition product, 2@Py-Ph, *via* a [2 + 2] cycloaddition reaction.^42^

As part of an in-depth study of the photochromic process, stable single crystals of Py-CH_3_ with an *E*-conformation (E-Py-CH_3_) were successfully grown by treating the Py-CH_3_ sample in chloroform under UV irradiation for 30 min, followed by slow evaporation at room temperature (Fig. S68). This result provides molecular-level visual evidence to confirm the molecular rotation from *Z* → *E* conformation under irradiation. Such evidence is beneficial for investigating the photochromic mechanism by molecular dynamics simulations.

**Fig. 8:**
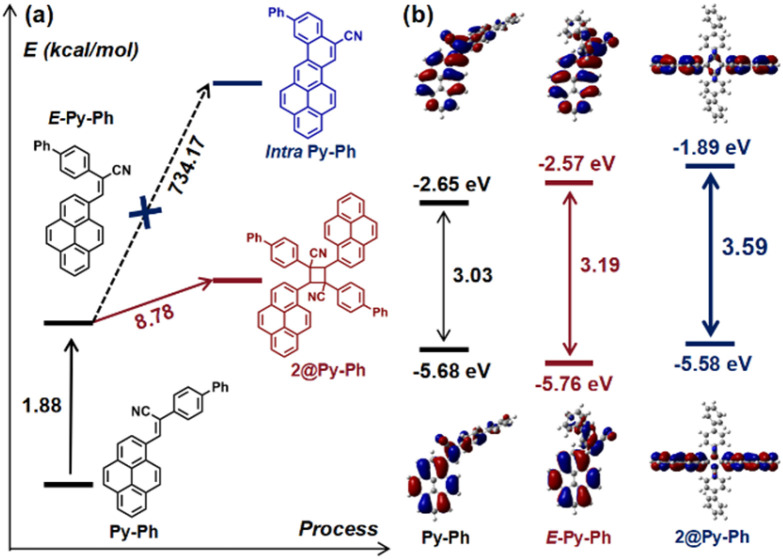
(a) Relative energy changes during the photochemical process of Py-Ph. (b) Energy gap and FMO of Py-Ph, E-Py-Ph and 2@Py-Ph.

To further understand the photochromic process at the molecular level, the relative energies of these molecules in *E*- and *Z*-conformations were calculated based on optimized geometric structures (Table S5). Taking Py-Ph as an example (Fig. 8), Py-Ph tends to adopt a *Z*-conformation in the ground state, and the energy barrier for the *Z* → *E* isomerization is 1.88 kcal mol^−1^ (a.u.), indicating that the molecule can easily overcome this energy barrier and transform into the *E*-conformation under extra stimulus. It can further undergo a [2 + 2] cycloaddition to form 2@Py-Ph through an endothermic process of 8.78 kcal mol^−1^. In contrast, due to a relatively high energy barrier of 734.17 kcal mol^−1^, Py-Ph cannot undergo intramolecular cyclization to form Intra-Py-Ph. Additionally, the calculated energy gap (Δ*E*_gap_) is 3.03 eV for Py-Ph, 3.19 eV for E-Py-Ph, and 3.59 eV for 2@Py-Ph, respectively. The enhanced Δ*E*_gap_ can promote the blue-shifted emission of the UV-irradiated Py-Ph sample, which is consistent with the experimental emission spectra. This conclusion is also applicable to both Py-R and Py-PR systems (Tables S5 and S6).

### Application in anti-counterfeiting and colourful ink

**Fig. 9:**
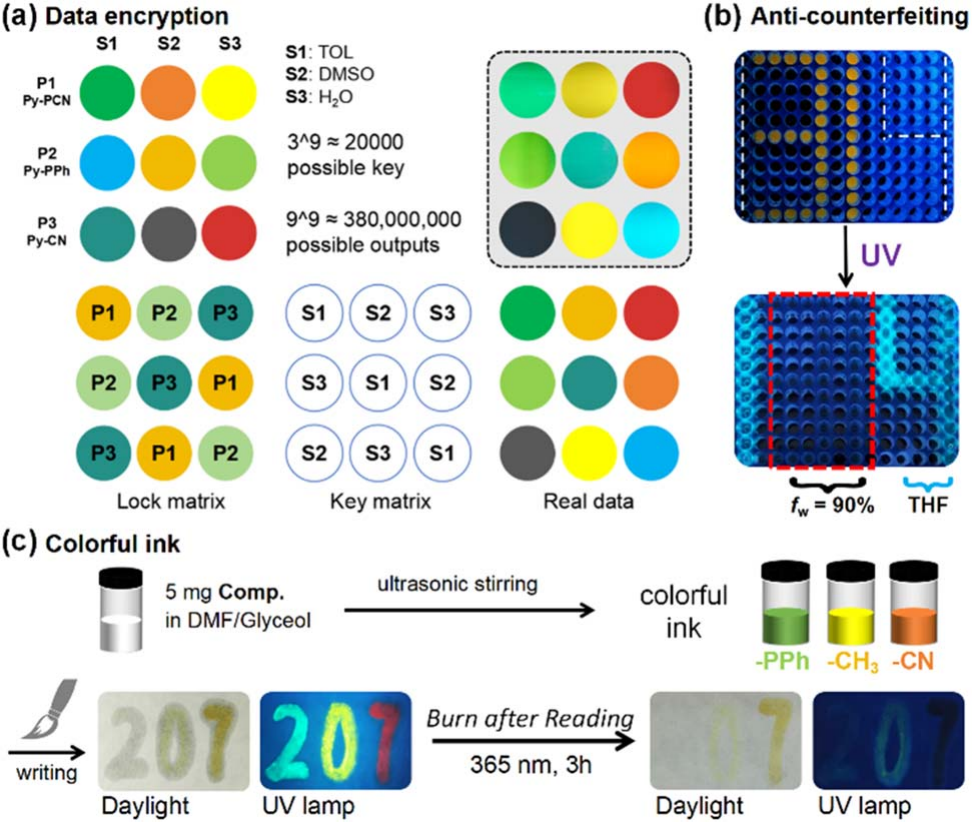
Application examples of Py-R and Py-PR. (a) Solvent-based data encryption using Py-PCN, Py-PPh and Py-CN. (b) Fluorescent anti-counterfeiting using Py-Ph in THF and THF/H_2_O mixture (*f*_w_ = 90%) in a 384-well PCR plate. (c) Schematic illustration of the preparation and application of colourful fluorescent ink.

The broad tunable emission of α-cyanostilbene pyrene-based molecules Py-R and Py-PR with AIE properties and photochromic properties could be very useful in fluorescence materials for enhancing information security.^43^ First, a solvent effect data-encryption method was developed.^44^ As shown in Fig. 9a, the 3 × 3 matrix containing three typical AIEgens vertically, that is Py-PCN (P1), Py-PPh (P2) and Py-CN (P3), is defined as the “lock” matrix. There are three different solvents along the horizontal axis that is toluene (S1), dimethyl sulfoxide (S2), and water (S3), which are defined as the “key” matrix. The 3 × 3 “output” matrix is generated by adding corresponding elements from both the “lock” and “key” matrices. For example, when combining S1 with P1, P2 and P3, a green, blue, and cyan output is generated, respectively. Therefore, only the “key” matrix containing three solvents in the correct positions will produce the desired “output” matrix. In theory, there are about 20 000 possible “key” and nearly 380 000 000 “output” results, but only one is correct. The above findings clearly illustrate the high level of data encryption security achieved by the proposed “lock–key” strategy.

The fluorescence contrast of Py-R in solution and the aggregated state, both before and after UV irradiation, enables its use in information storage through a controlled “turn-on” or “turn-off” mode. As shown in Fig. 9b, Py-Ph was dissolved in THF and in a THF/H_2_O mixture (*f*_w_ = 90%), respectively. These solutions were used to create a “1314” pattern (the symbol represents “*Forever and Ever*” in Chinese culture) in a 384-well PCR plate, where the symbols “1” and “4” were filled with THF solution, and “31” with the *f*_w_ = 90% solution. Before UV irradiation, a distinct “31” pattern with bright yellow fluorescence was observed, attributable to the emission from the aggregates. Upon exposure to a hand-held UV lamp, the “31” pattern gradually faded, and subsequently a “14” pattern emerged with bright blue emission because of photochromism. Py-Ph thus serves as an ideal 2D fluorescence anti-counterfeiting material with a high signal-to-noise ratio.

To further explore the potential of these compounds for information storage, Py-PPh, Py-CH_3_, and Py-CN (*ca.* 1 mg) were dissolved in a mixture of DMF (1 mL) and glycerol (5 mL) to prepare green, yellow, and red fluorescent inks. These inks were stored in brown bottles for several months and remained stable. They were used for Chinese handwriting with a traditional Chinese brush. As shown in Fig. 9c, the number “207” was easily written on filter paper using green, yellow, and red inks, which exhibited bright fluorescence under UV irradiation. The handwriting showed only a mild trace in daylight but could be almost completely erased by UV irradiation, achieving a “*Burn after Reading*” effect. This process demonstrates the efficiency of pyrene-based cyanostilbene molecules as erasable fluorescent inks, providing multistage data security protection. In summary, the unique combination of AIE and photochromic properties in α-cyanostilbene pyrene-based molecules offers a versatile platform for high-security data encryption and information storage. The proposed methods and materials hold significant potential for practical applications in the field of information security.

## Conclusions

In summary, a series of pyrene-based AIEgens (Py-R and Py-PR) were designed and synthesized *via* the Knoevenagel reaction. They exhibit a pronounced AIE characteristic with broad colour-tunable emission ranging from 500 to 667 nm, and enhanced *Ф*_f_ from 4% to 61% *via* regulating the end groups. Experimental and theoretical studies reveal that the electronic effect of the end group plays a crucial role in determining the optical properties and crystal packing of these α-cyanostilbene pyrene-based AIEgens. By tuning the energy gap and electrostatic potential (ESP) distribution, which correlates with the Hammett constant, we demonstrate significant control over their emission behaviour as much as possible. Importantly, all compounds undergo *Z* → *E* isomerization and intramolecular [2 + 2] cycloaddition reactions, resulting in remarkable photochromic behaviour under UV irradiation both in solution and aggregated states.

Based on the AIE properties, broad colour-tunable emission, and photochromic properties of these AIEgens, the selected cyan (Py-PPh), yellow (Py-CH_3_) and red (Py-CN) emitters were utilized as advanced fluorescent materials for the preparation of colourful fluorescent ink for information storage, and the hidden information can be destroyed after reading, achieving a “*Burn After Reading*” effect. Thus, this work not only provides a new molecular strategy for developing broad colour-tunable emission AIEgens *via* precisely tuning the end groups but also offers insights into understanding the influence of end-group electronic effects on aggregation mode and emission behaviour. Furthermore, it explores the potential applications of such advanced molecules in multistage data security protection.

## Author contributions

C. Y. Zeng: methodology, data curation, investigation, writing – original draft. S. Liang: data curation, investigation. J. Y. Lin: data curation. W. Liu: data curation, software. Z. X. Xie: data curation, software. W. X. Cai: data curation. C. Redshaw: writing – review & editing. X. Feng: conceptualization, funding acquisition, resources, supervision, writing – review & editing. B. Z. Tang: writing – review & editing, software, conceptualization.

## Conflicts of interest

There are no conflicts to declare.

## Supplementary Material

SC-016-D5SC03105B-s001

SC-016-D5SC03105B-s002

## Data Availability

The data supporting this article have been included as part of the SI. Crystallographic data for Py-CF_3_, Py-PPh, (*E*)-Py-CH_3_ and Py-OMe have been deposited at the CCDC: 2433820, 2433821, 2433819, and 1529865.^45–48^ Detailed experimental procedures, characterization data of the compounds (NMR, HRMS, UV-vis and emission spectra and X-ray single-crystal diffraction analysis) and DFT calculation data. See DOI: https://doi.org/10.1039/d5sc03105b.
