# Supplementary material for: Achieving controllable packing mode and broad colour-tunable emission via the end group effect in pyrene-based aggregation-induced emission luminogens
Source: Chem Sci. 2025 Aug 19;16(36):16719–28. doi: 10.1039/d5sc03105b (PMC12362467; doi:10.1039/d5sc03105b)
Supplement: SC-016-D5SC03105B-s001 [file SC-016-D5SC03105B-s001.pdf]

Supplementary Information (SI) for Chemical Science.
This journal is © The Royal Society of Chemistry 2025

Achieving controllable packing mode and broad color-tunable emission via end group effect in pyrene-based aggregation-induced emission luminogens

Chongyang Zeng,[a] Shan Liang,[a] Jieyu Lin,[a] Wei Liu,[a] Zhixin Xie,[a] Wenxuan Cai,[a] Carl Redshaw,[b] Xing Feng,*[a] Ben Zhong Tang*[c]

[a] *Guangdong Provincial Key Laboratory of Functional Soft Condensed Matter, Guangdong Provincial Key Laboratory of Information Photonics Technology, School of Material and Energy Guangdong University of Technology Guangzhou 510006, P. R. China;*

[b] *Chemistry, School of Natural Sciences, University of Hull, Hull, Yorkshire HU6 7RX, UK;*

[c] *School of Science and Engineering, Shenzhen Institute of Aggregate Science and Technology, The Chinese University of Hong Kong Shenzhen, Guangdong 518172, P. R. China.*

Email: hyxhn@sina.com (X. Feng); tangbenz@cuhk.edu.cn (B. Z. Tang)

Table of Content

1. Experimental Section S1

2. NMR spectra S5

3. High-resolution mass spectrometry (HRMS) S13

4. Photophysical Properties S18

5. X-ray single crystal diffraction analysis S35

6. DFT calculation S39

7. Photochromism Properties S41

1. Experimental Section

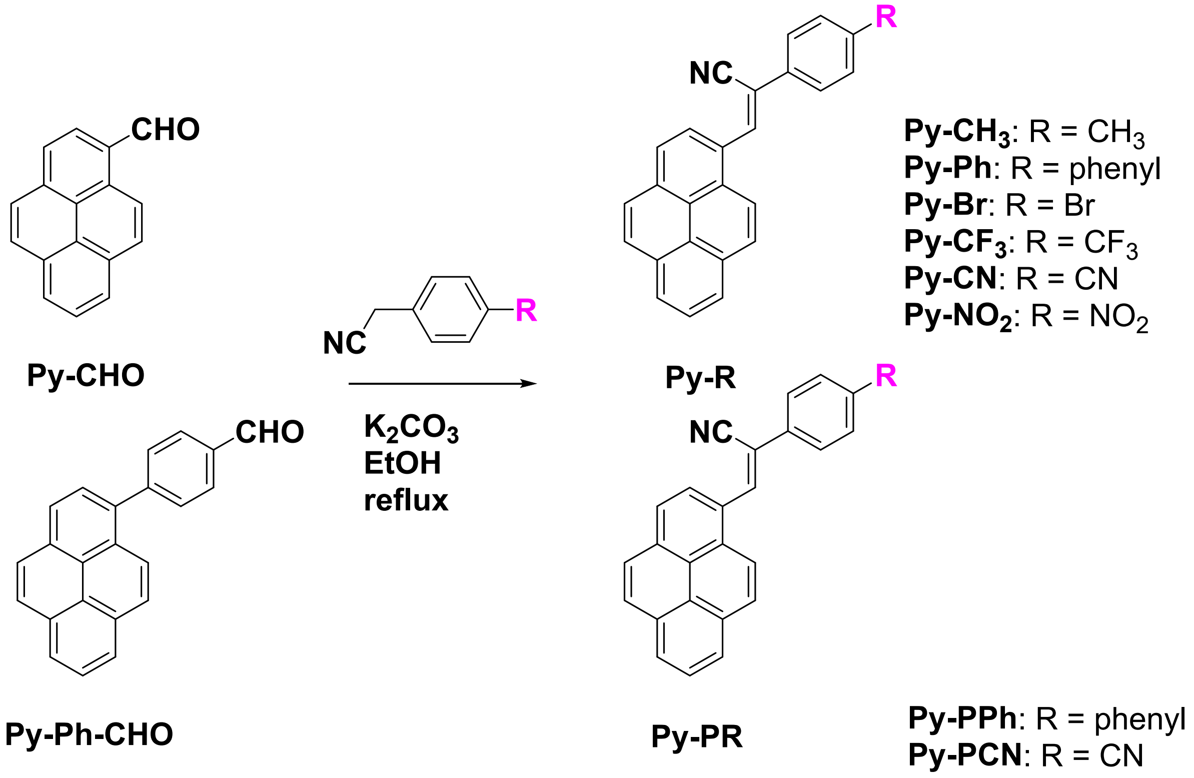

Scheme S1. The synthetic pathways of molecules **Py-R** and **Py-PR**.

1.1 Materials

Unless otherwise stated, all reagents and solvents used were purchased from commercial sources and were used without further purification. 4-(Pyren-1-yl)benzaldehyde was synthesized according to the previous report.

1.2. Characterization

^{1}H, ^{13}C and ^{19}F NMR spectra were recorded on a Bruker AVANCE III 400/600 MHz spectrometer using $CDCl_3$ as the solvent ($CHCl_3$, ^{1}H: $\delta = 7.26$ ppm, ^{13}C: $\delta = 77.06$ ppm). High-resolution mass spectra (HRMS) were recorded using an LC/MS/MS, which consisted of a High-Performance Liquid Chromatography (HPLC) system (Ultimate 3000 RSLC, Thermo Scientific, USA) and a Q Exactive Orbitrap mass spectrometer. UV-Vis absorption spectra and photoluminescence (PL) spectra were recorded on a Shimadzu UV-2600 and a Hitachi F-4700 fluorescence spectrometer. PL quantum yields were measured using absolute methods using a Hamamatsu C11347-11 Quantaurus-QY Analyzer. The quantum chemistry calculations were performed using the Gaussian 09 (B3LYP/6-31 G$^+$ (d,p) basis set) software package.

Crystallographic data were collected on a Bruker APEX 2 CCD diffractometer with graphite monochromated Mo K_α radiation (λ = 0.71073 Å) in the ω scan mode or on a Rigaku AFC12 diffractometer equipped with a HyPix detector and a rotating anode source, also with Mo K_α radiation.

1.3 General synthetic procedure for compounds Py-R and Py-PR

Compounds **Py-R** and **Py-PR** were synthesized from 1-pyrenecarboxaldehyde or 4-(pyren-1-yl)benzaldehyde using the corresponding phenylacetonitrile derivatives by a Knoevenagel condensation reaction in high yield.

Synthesis of Py-CH_3: A mixture of 1-pyrenecarboxaldehyde (100.0 mg, 0.43 mmol, 1 eq.) and the phenylacetonitrile derivative (68.2 mg, 0.52 mmol, 1.2 eq.) in ethanol (15 mL) at room temperature was stirred under argon, and saturated K_2CO_3 (2 mL) was added. The mixture was refluxed at 75 °C for 24 h. After cooling to room temperature, the mixture was filtered and washed twice with ethanol. The obtained solid was recrystallized from a mixture of dichloromethane and methanol to obtain the target compound **Py-R** as a yellow solid (95 mg, 65% yield). ^{1}H NMR (400 MHz, $CDCl_3$) δ 8.63 (s, 1H), 8.53 (s, 1H), 8.25 (m, 4H), 8.19-8.12 (m, 2H), 8.11-8.02 (m, 2H), 7.74 (d, J = 8.1 Hz, 2H), 7.33 (d, J = 8.0 Hz, 2H), 2.45 (s, 3H) ppm. ^{13}C NMR (100 MHz, $CDCl_3$) δ 139.7, 139.3, 132.7, 131.7, 131.3, 130.7, 129.8, 129.7, 128.6, 128.1, 127.4, 126.3, 126.1, 126.1, 125.9, 124.9, 124.7, 122.6, 118.3, 114.8, 21.3 ppm. HRMS (ESI) m/z (100%) calcd for $C_{26}H_{17}N$ $[M+Na^+]^+$: 366.1253, found: 366.1243.

Py-Ph was obtained as a yellow solid (117 mg, 67% yield). ^{1}H NMR (400 MHz, $CDCl_3$) δ 8.67 (d, J = 8.1 Hz, 1H), 8.62 (s, 1H), 8.27 (m, 4H), 8.17 (m, 2H), 8.12-8.03 (m, 2H), 7.92 (d, J = 8.3 Hz, 2H), 7.76 (d, J = 8.3 Hz, 2H), 7.68 (d, J = 7.4 Hz, 2H), 7.50 (t, J = 7.6 Hz, 2H), 7.41 (t, J = 7.3 Hz, 1H). ^{13}C NMR (100 MHz, $CDCl_3$) δ 142.2, 140.0, 133.4, 132.8, 131.3, 130.7, 129.9, 129.0, 128.8, 128.0, 127.9, 127.8, 127.4, 127.1, 126.6, 126.38, 126.28, 126.1, 126.0, 125.0, 124.7, 122.6, 118.2, 114.4, 100.1 ppm. HRMS (ESI) m/z (100%) calcd for $C_{31}H_{19}N$ $[M+Na^+]^+$: 428.1409, found: 428.1405.

Py-Br was obtained as a yellow solid (130 mg, 74%). ^{1}H NMR (600 MHz, $CDCl_3$) δ 8.59 (d, J = 8.0 Hz, 1H), 8.53 (s, 1H), 8.20 (dd, J = 8.7, 5.3 Hz, 4H), 8.14 (d, J = 9.2 Hz, 1H), 8.10 (d, J = 8.8 Hz, 1H), 8.05 (d, J = 8.8 Hz, 1H), 8.00 (t, J = 7.6 Hz, 1H), 7.65 (d, J = 8.5 Hz, 2H), 7.60 (d, J = 8.4 Hz, 2H) ppm. HRMS (ESI) m/z (100%) calcd for $C_{25}H_{14}BrN$ $[M+Na^+]^+$:

430.0202, found: 430.0196.

Py-CF$_3$ was obtained as an orange-yellow solid (131 mg, 75% yield). ^{1}H NMR (400 MHz, $CDCl_3$) δ 8.66 (d, J = 8.1 Hz, 1H), 8.61 (s, 1H), 8.26-8.23 (m, 2H), 8.23-8.21 (m, 1H), 8.19 (d, J = 6.3 Hz, 2H), 8.16-8.13 (m, 1H), 8.09 (s, 1H), 8.07 (d, J = 2.1 Hz, 1H), 8.04 (d, J = 7.6 Hz, 1H), 7.92 (d, J = 8.2 Hz, 2H), 7.76 (d, J = 8.3 Hz, 2H) ppm. ^{13}C NMR (100 MHz, $CDCl_3$) δ 142.2, 137.9, 133.2, 131.2, 130.6, 130.0, 129.1, 129.0, 127.4, 127.2, 126.5, 126.27, 126.2, 126.1, 126.0, 125.0, 124.7, 124.4, 122.2, 117.7, 113.1 ppm. ^{19}F NMR (376 MHz, $CDCl_3$) δ -62.66 ppm (-**CF$_3$**). HRMS (ESI) m/z (100%) calcd for $C_{26}H_{14}F_3N$ [M+Na$^+$]$^+$: 420.0970, found: 420.0967.

Py-CN was obtained as an orange-red solid (114 mg, 74% yield). ^{1}H NMR (400 MHz, $CDCl_3$) δ*:* 8.71 (s, 2H), 8.23 (dd, J = 26.9, 10.3 Hz, 6H), 8.15-8.06 (m, 2H), 7.95 (d, J = 6.7 Hz, 2H), 7.82 (s, 2H) ppm. ^{13}C NMR (100 MHz, $CDCl_3$) δ 142.9, 132.9, 129.3, 129.2, 127.4, 126.9, 126.7, 126.5, 126.4, 126.1, 125.0, 122.1, 118.2, 112.7 ppm. HRMS (ESI) m/z (100%) calcd for $C_{26}H_{14}N_2$ [M+Na$^+$]$^+$: 377.1049, found: 377.1040.

Py-NO$_2$ was obtained as a red solid (125 mg, 78% yield). ^{1}H NMR (600 MHz, $CDCl_3$) δ 8.78 (s, 1H), 8.74 (d, J = 8.1 Hz, 1H), 8.40 (d, J = 8.8 Hz, 2H), 8.31 (s, 2H), 8.29 (d, J = 6.5 Hz, 2H), 8.26-8.23 (m, 1H), 8.21 (d, J = 8.9 Hz, 1H), 8.14 (d, J = 9.0 Hz, 1H), 8.10 (t, J = 7.6 Hz, 1H), 8.03 (d, J = 8.8 Hz, 2H) ppm. HRMS (ESI) m/z (100%) calcd for $C_{25}H_{14}NO_2$ [M+H]$^+$: 374.1055, found: 374.1070.

Py-PPh was obtained as a yellow solid **Py-PPh** (127 mg, 68% yield). ^{1}H NMR (400 MHz, $CDCl_3$) δ 8.26 (d, J = 7.9 Hz, 1H), 8.24-8.19 (m, 3H), 8.14 (s, 1H), 8.12 (d, J = 1.9 Hz, 3H), 8.08 (d, J = 9.3 Hz, 1H), 8.05 (d, J = 7.6 Hz, 1H), 8.03-8.00 (m, 1H), 7.84 (d, J = 8.5 Hz, 2H), 7.78 (d, J = 8.2 Hz, 2H), 7.75-7.70 (m, 3H), 7.68-7.64 (m, 2H), 7.49 (t, J = 7.5 Hz, 2H), 7.40 (t, J = 7.3 Hz, 1H). ^{13}C NMR (100 MHz, $CDCl_3$) δ 143.6, 142.1, 141.4, 140.0, 136.4, 133.4, 132.7, 131.4, 131.2, 131.0, 130.9, 129.4, 128.9, 128.4, 127.9, 127.7, 127.4, 127.4, 127.0, 126.4, 126.1, 125.3, 125.0, 125.0, 124.9, 124.7, 118.1, 111.2 ppm. HRMS (ESI) m/z (100%) calcd for $C_{37}H_{23}N$ [M+Na$^+$]$^+$: 504.1722, found: 504.1729.

Py-PCN was obtained as a yellow solid (122 mg, 72% yield). ^{1}H NMR (600 MHz, $CDCl_3$) δ 8.25 (d, J = 7.8 Hz, 1H), 8.23 (d, J = 7.6 Hz, 1H), 8.20 (d, J = 7.6 Hz, 1H), 8.18 (d, J = 9.3 Hz, 1H), 8.15-8.10 (m, 4H), 8.09-8.03 (m, 2H), 8.00 (d, J = 7.8 Hz, 1H), 7.84 (d, J = 8.3 Hz, 2H), 7.80 (d, J = 8.1 Hz, 2H), 7.77 (s, 1H), 7.75 (d, J = 8.8 Hz, 2H). ^{13}C NMR (150

MHz, $CDCl_3$) δ 144.7, 144.4, 138.8, 136.0, 132.8, 131.8, 131.4, 131.4, 131.1, 130.8, 129.8, 128.3, 128.0, 127.9, 127.3, 126.5, 126.2, 125.4, 125.1, 125.0, 124.8, 124.7, 124.6, 118.2, 117.3, 112.7, 109.7 ppm. HRMS (ESI) m/z (100%) calcd for $C_{32}H_{18}N_2$ $[M]^+$: 430.1470, found: 430.1482.

2. NMR spectra

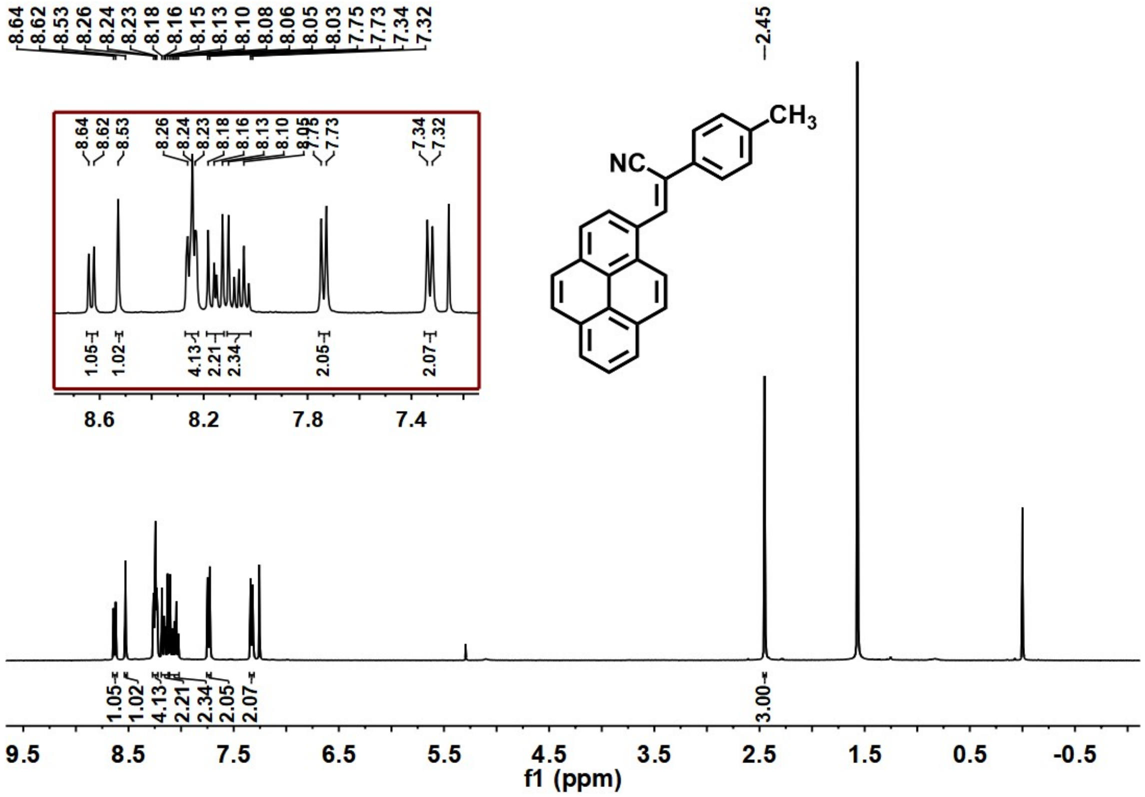

Figure S1. ^{1}H NMR spectra (400 Hz, $CDCl_3$) of **Py-CH_3**.

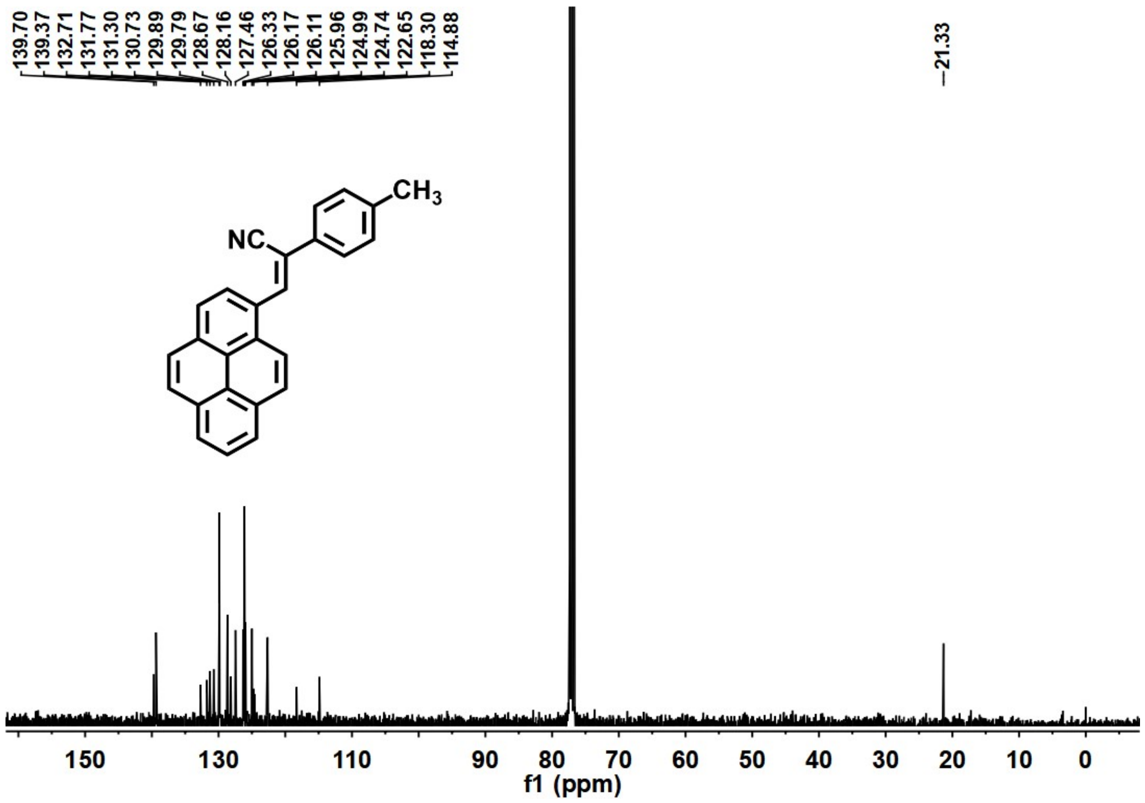

Figure S2. ^{13}C NMR spectra (100 Hz, $CDCl_3$) of **Py-CH_3**.

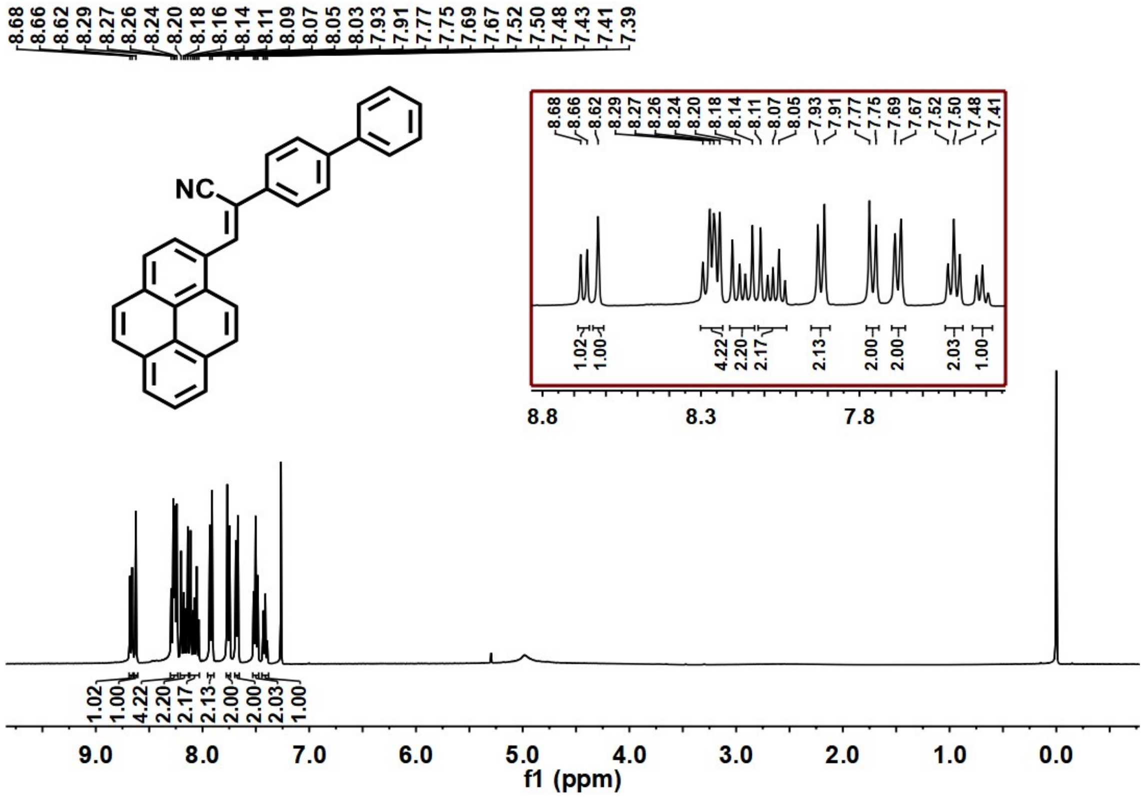

Figure S3. ^{1}H NMR spectra (400 Hz, $CDCl_3$) of **Py-Ph**.

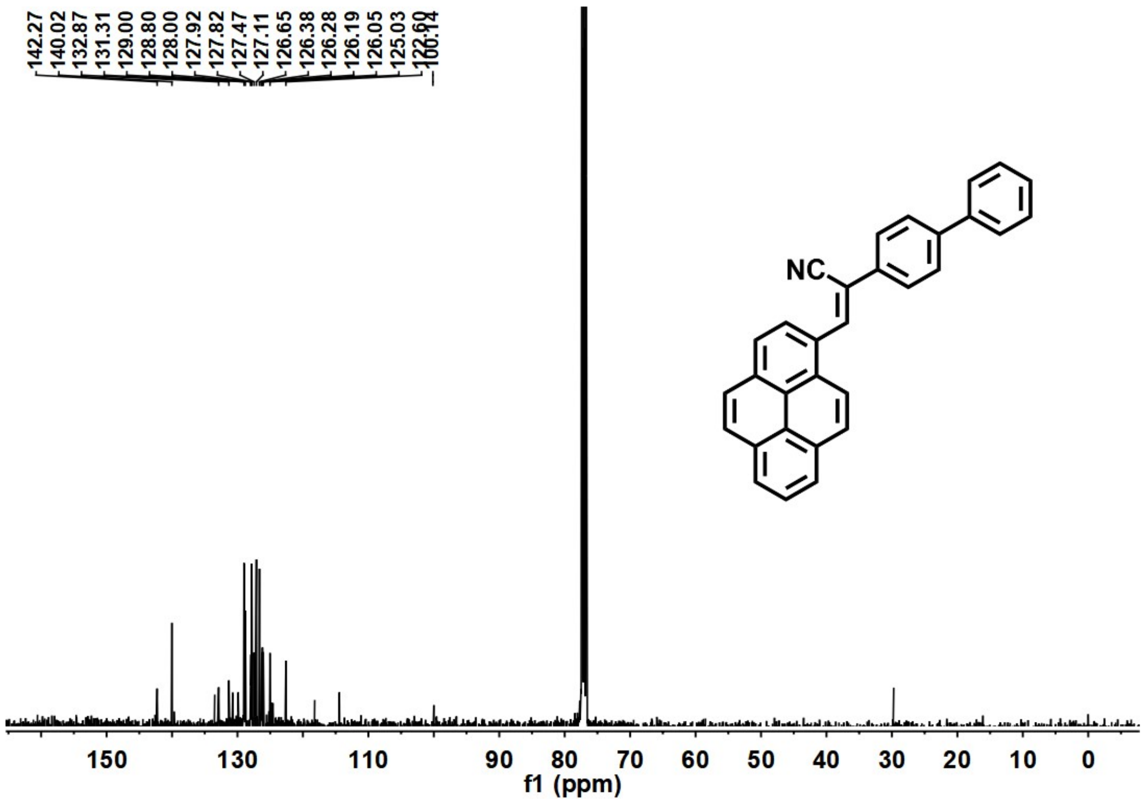

Figure S4. ^{13}C NMR spectra (100 Hz, $CDCl_3$) of **Py-Ph**.

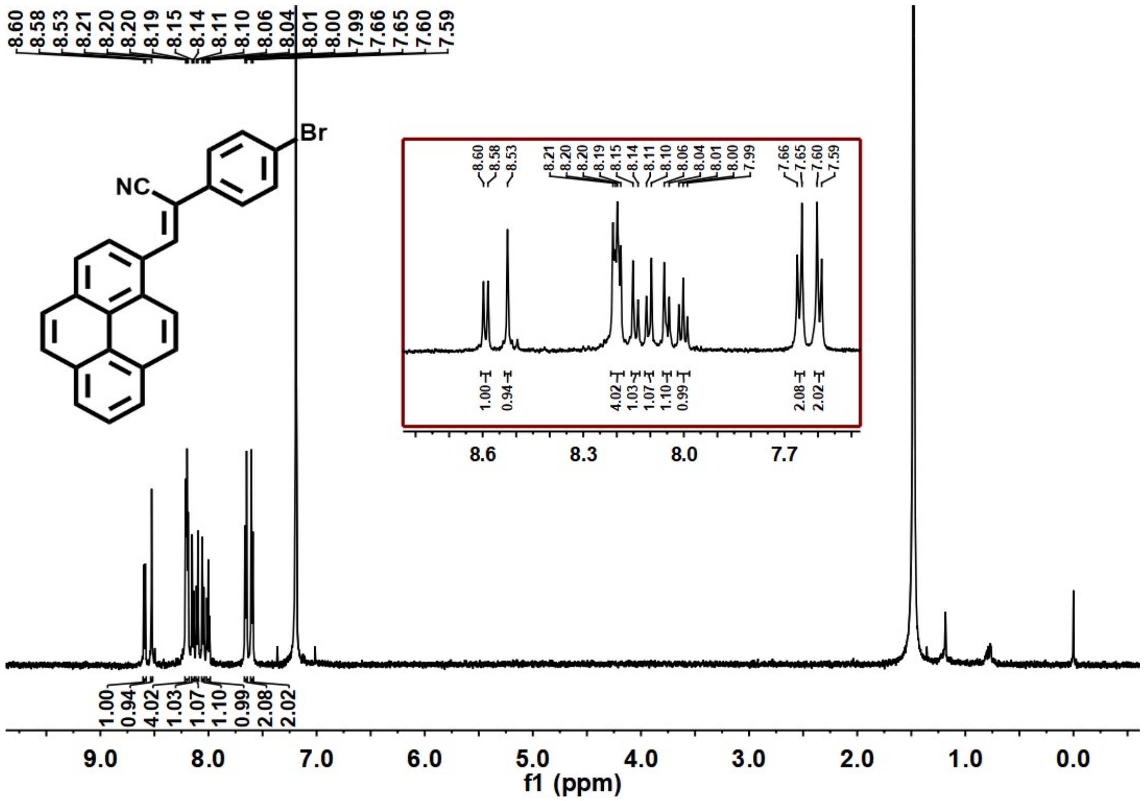

Figure S5. ^{1}H NMR spectra (600 Hz, $CDCl_3$) of **Py-Br**.

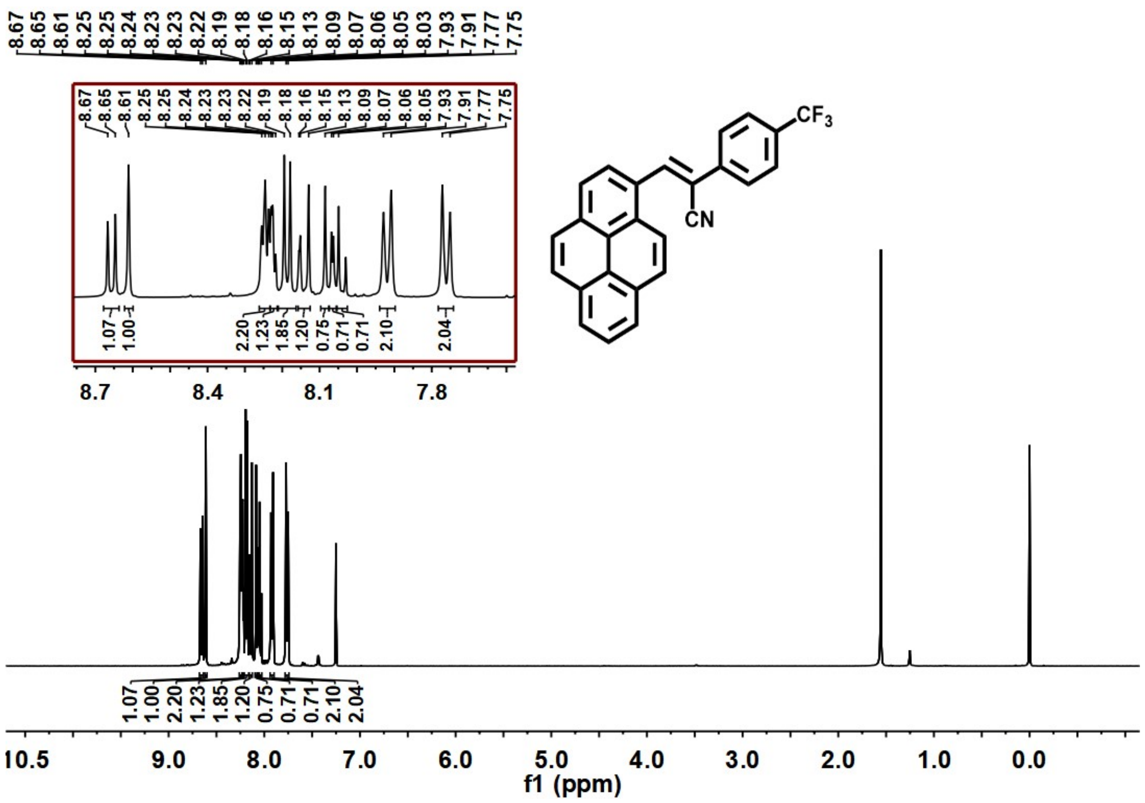

Figure S6. ^{1}H NMR spectra (400 Hz, $CDCl_3$) of **Py-CF$_3$**.

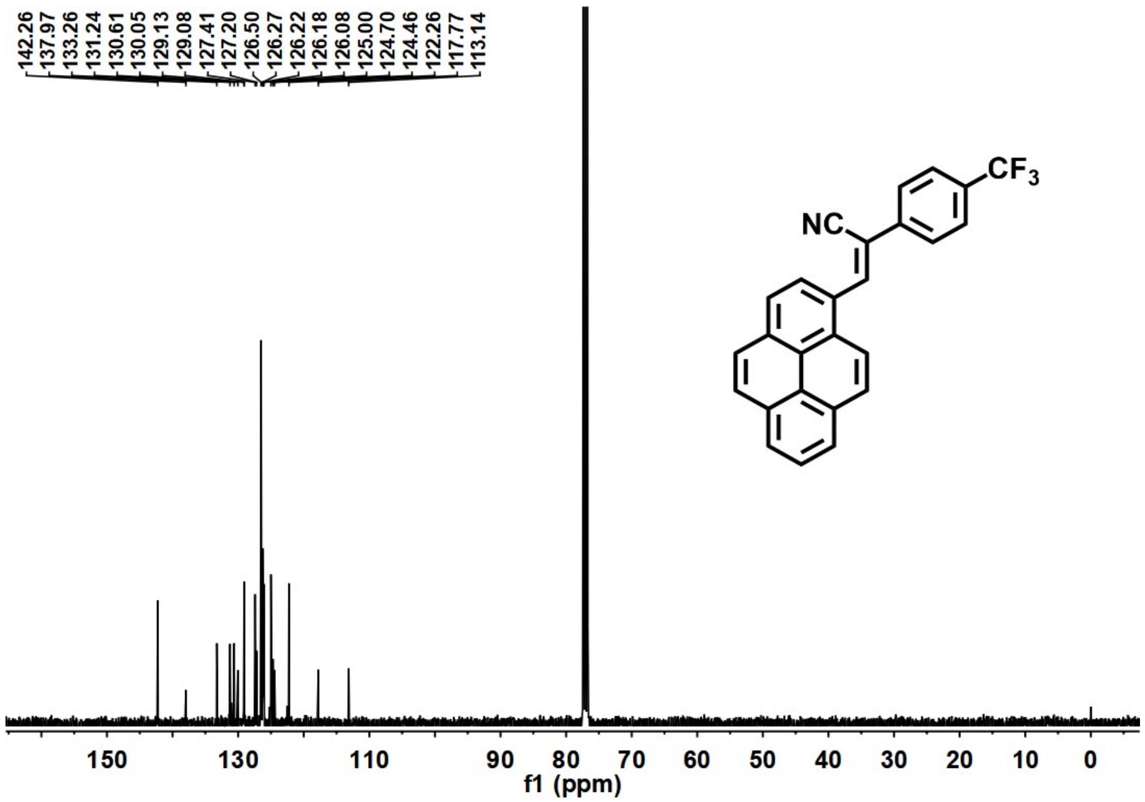

Figure S7. ^{13}C NMR spectra (100 Hz, $CDCl_3$) of **Py-CF$_3$**.

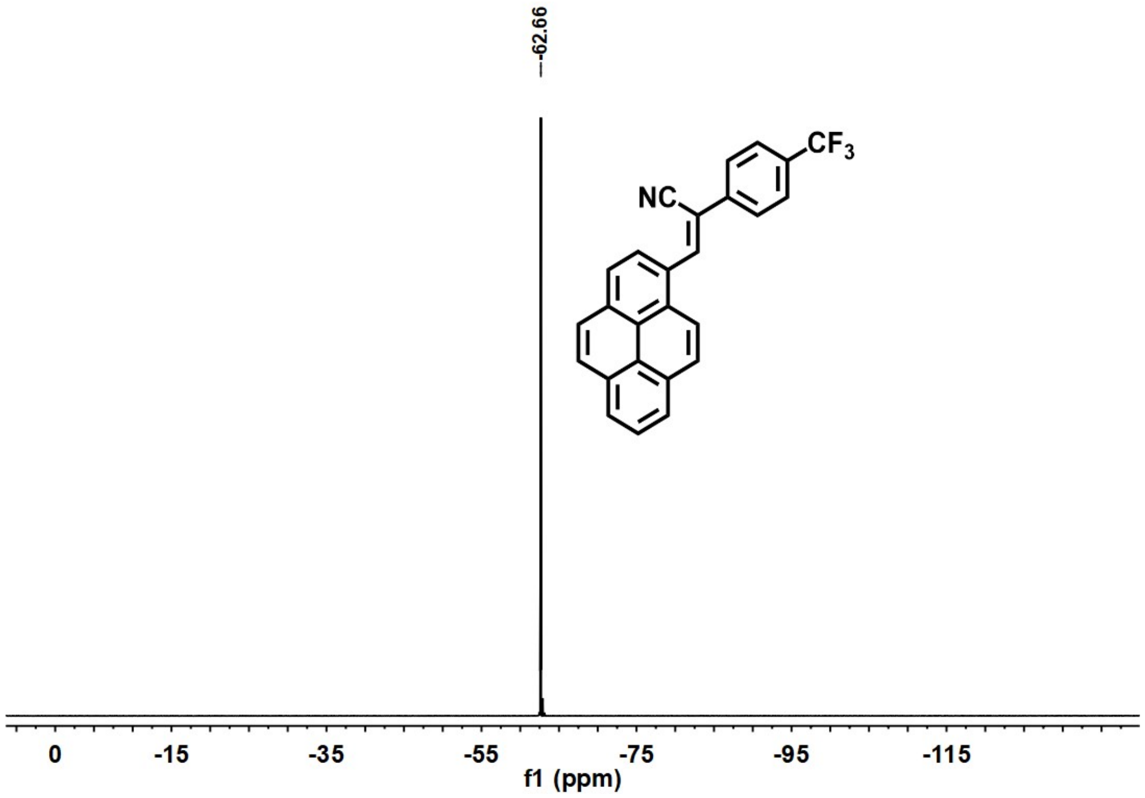

Figure S8. ^{19}F NMR spectra (376 Hz, $CDCl_3$) of **Py-CF$_3$**.

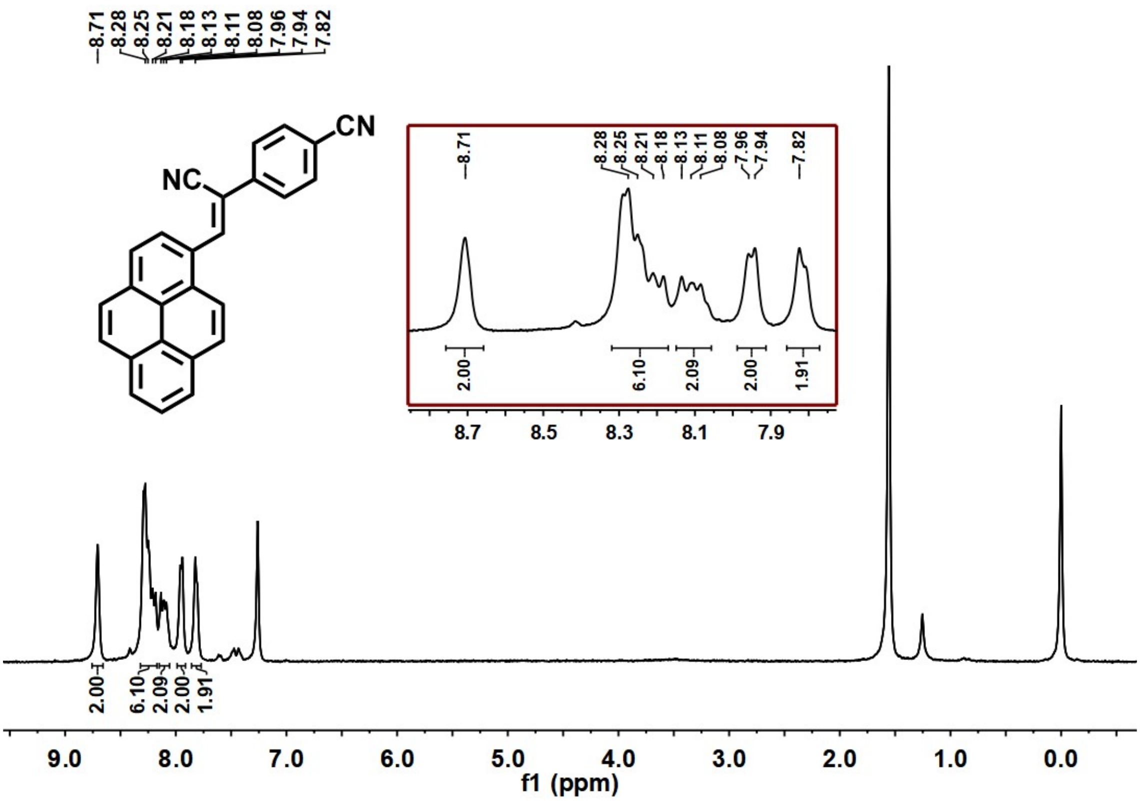

Figure S9. ^{1}H NMR spectra (400 Hz, $CDCl_3$) of **Py-CN**.

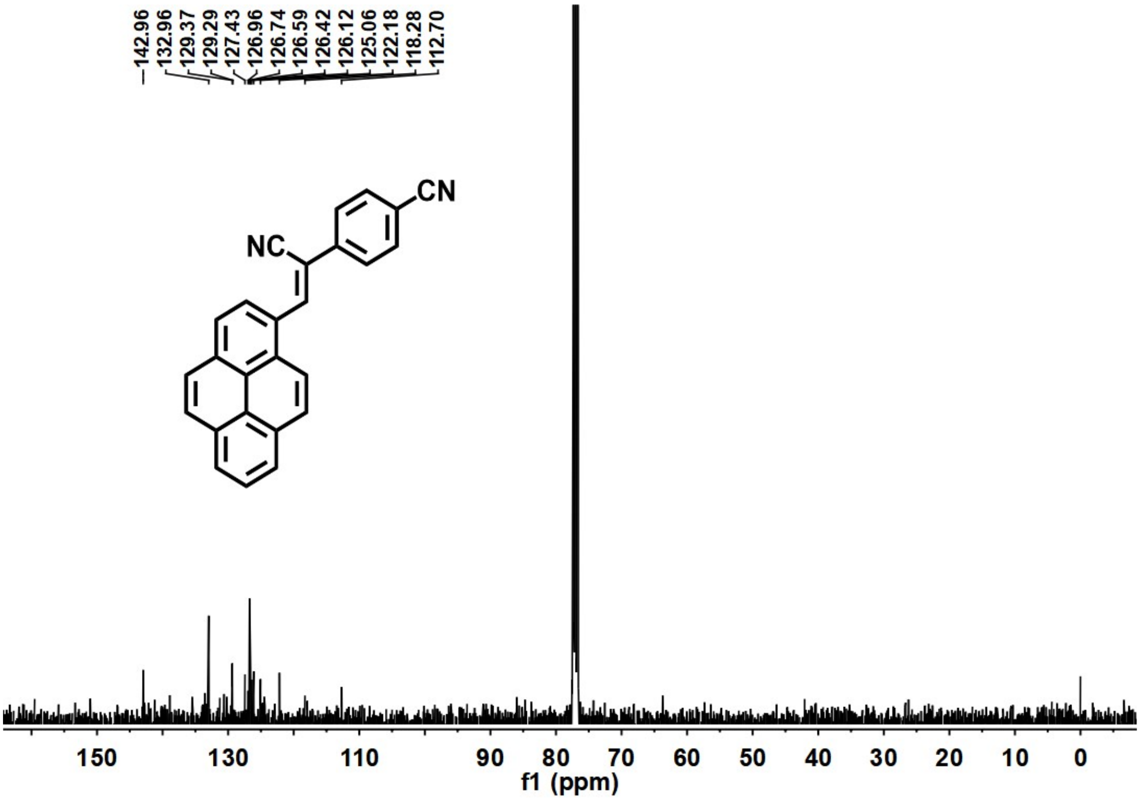

Figure S10. ^{13}C NMR spectra (100 Hz, $CDCl_3$) of **Py-CN**.

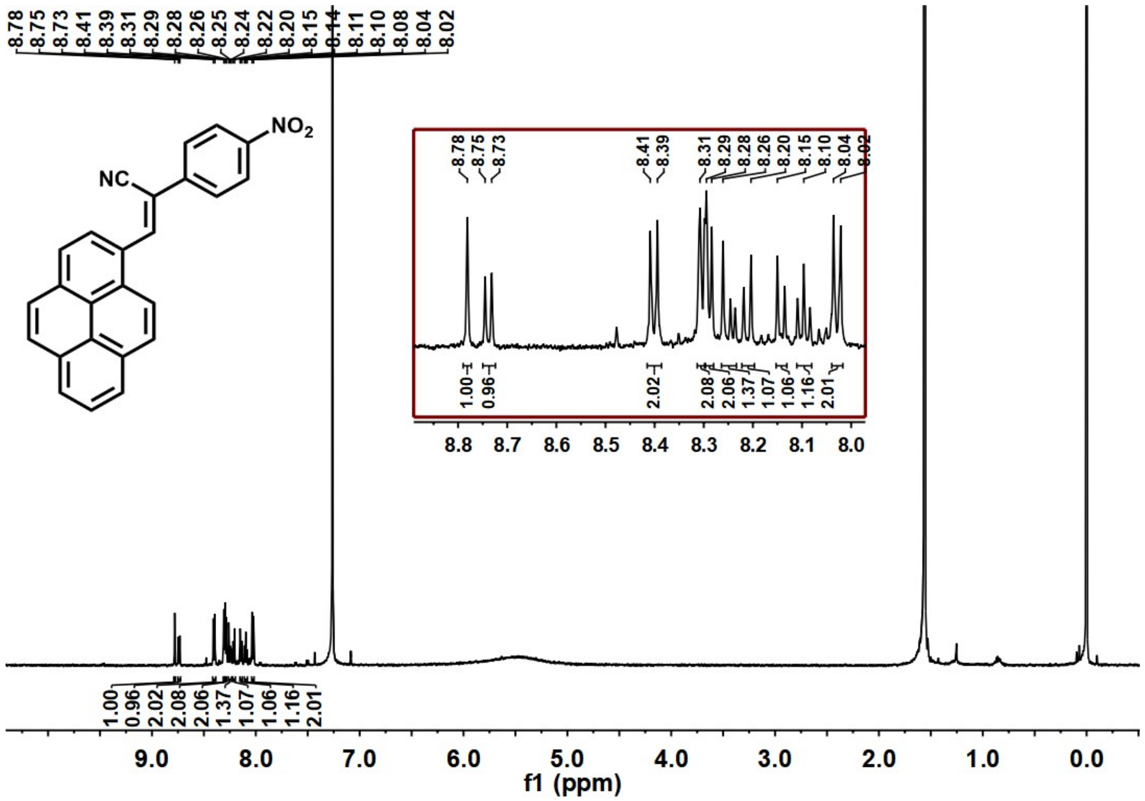

Figure S11. ^{1}H NMR spectra (600 Hz, $CDCl_3$) of **Py-NO$_2$**.

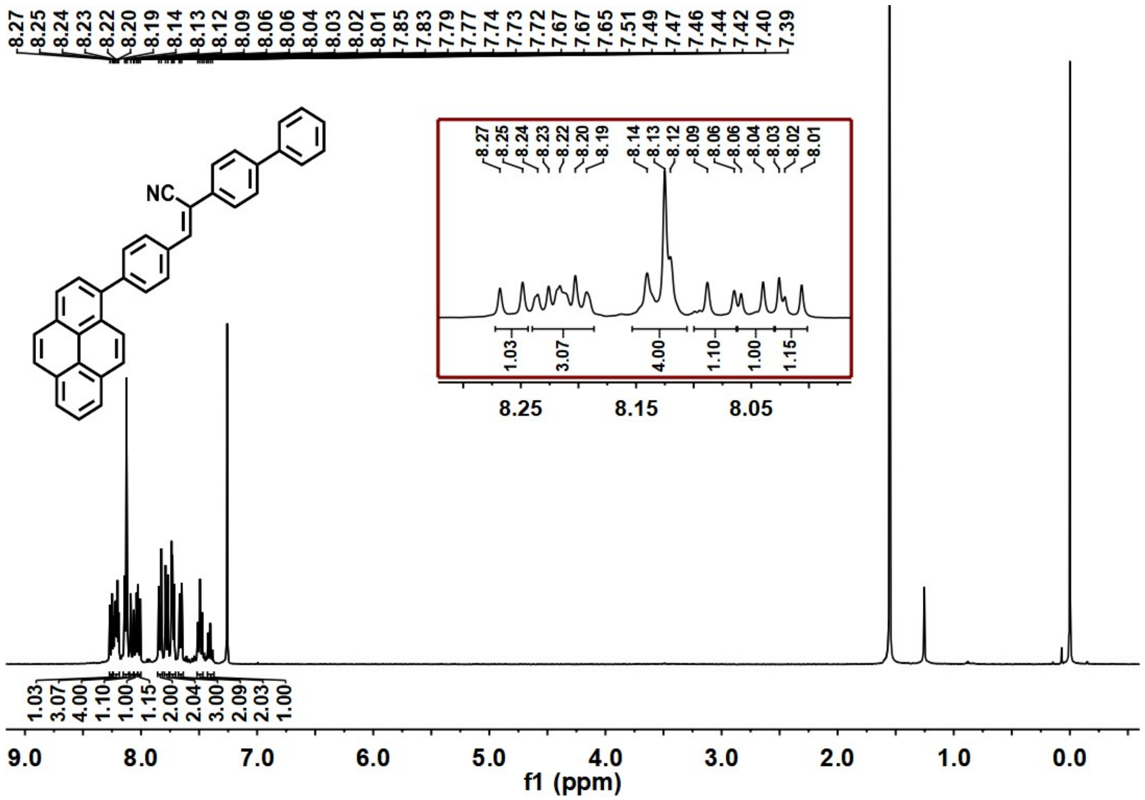

Figure S12. ^{1}H NMR spectra (400 Hz, $CDCl_3$) of **Py-PPh**.

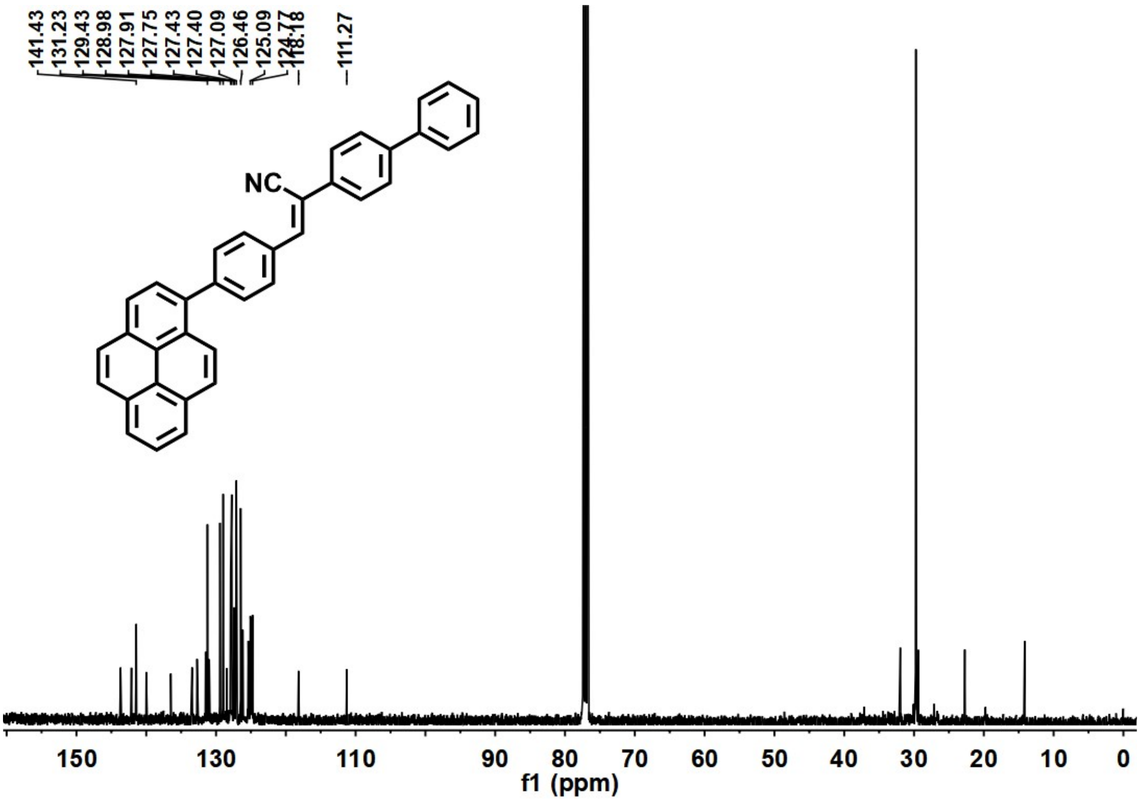

Figure S13. ^{13}C NMR spectra (100 Hz, $CDCl_3$) of **Py-PPh**.

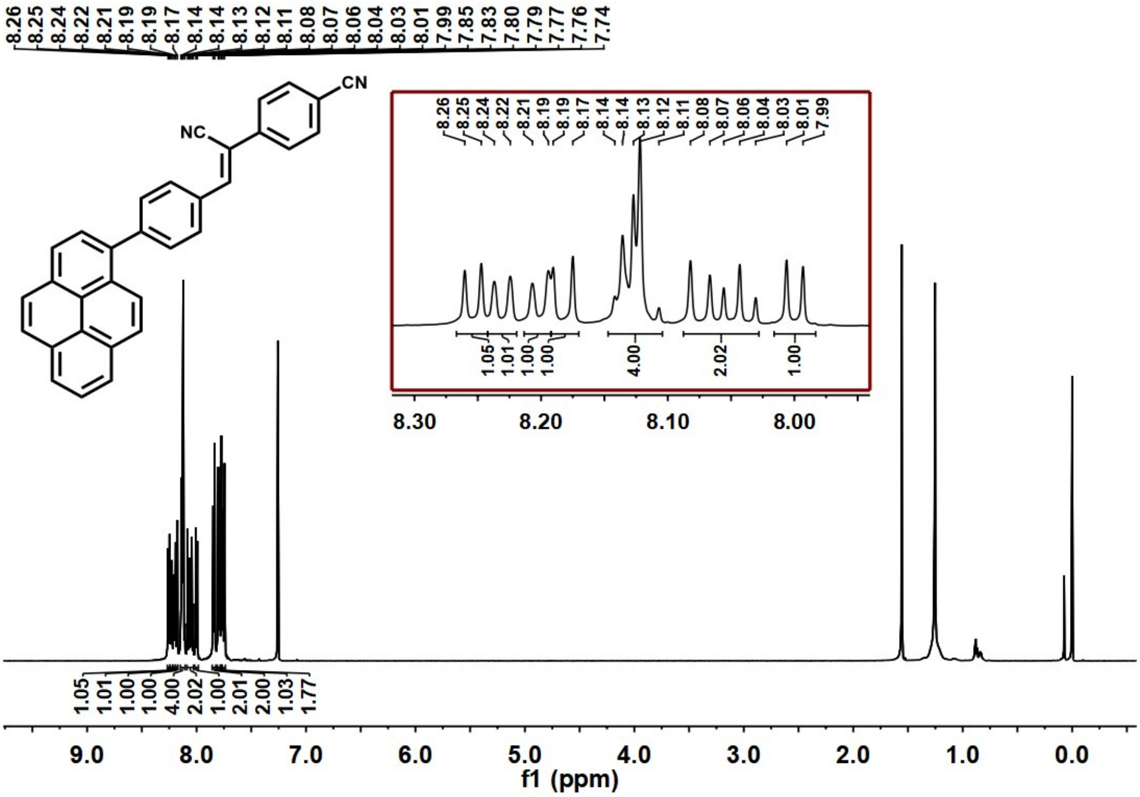

Figure S14. ^{1}H NMR spectra (600 Hz, $CDCl_3$) of **Py-PCN**.

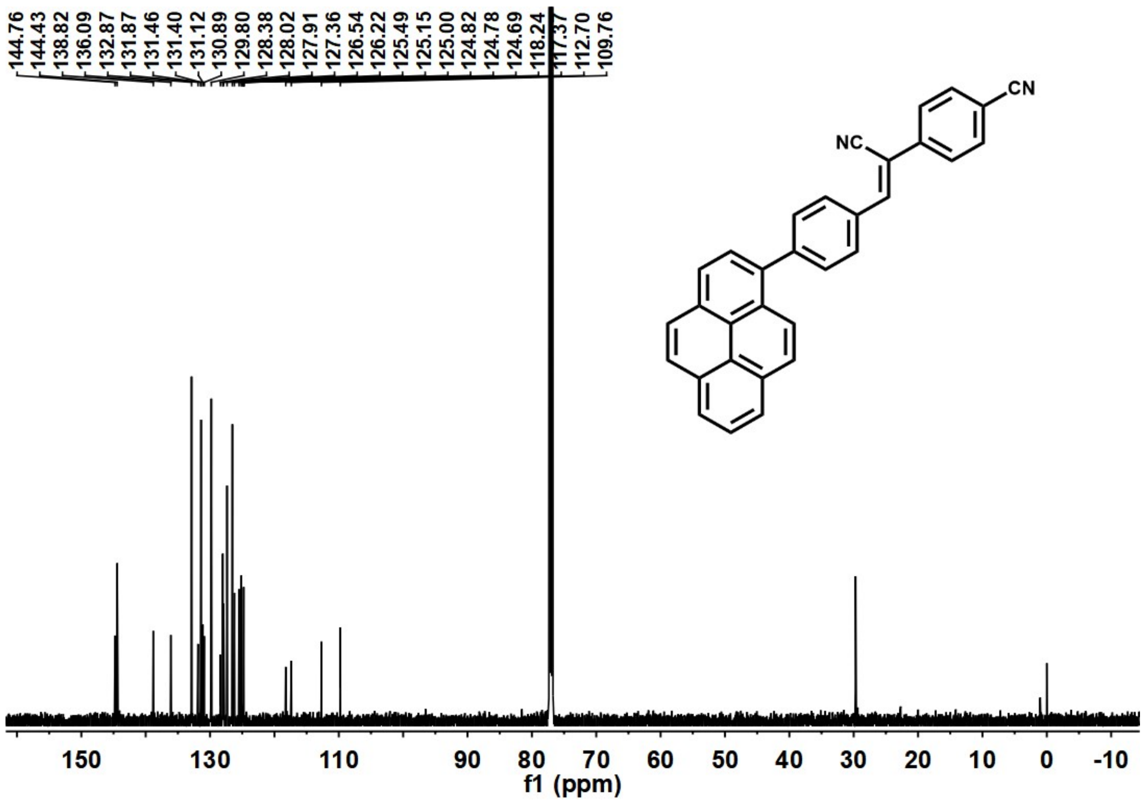

Figure S15. ^{13}C NMR spectra (150 Hz, $CDCl_3$) of **Py-PCN**.

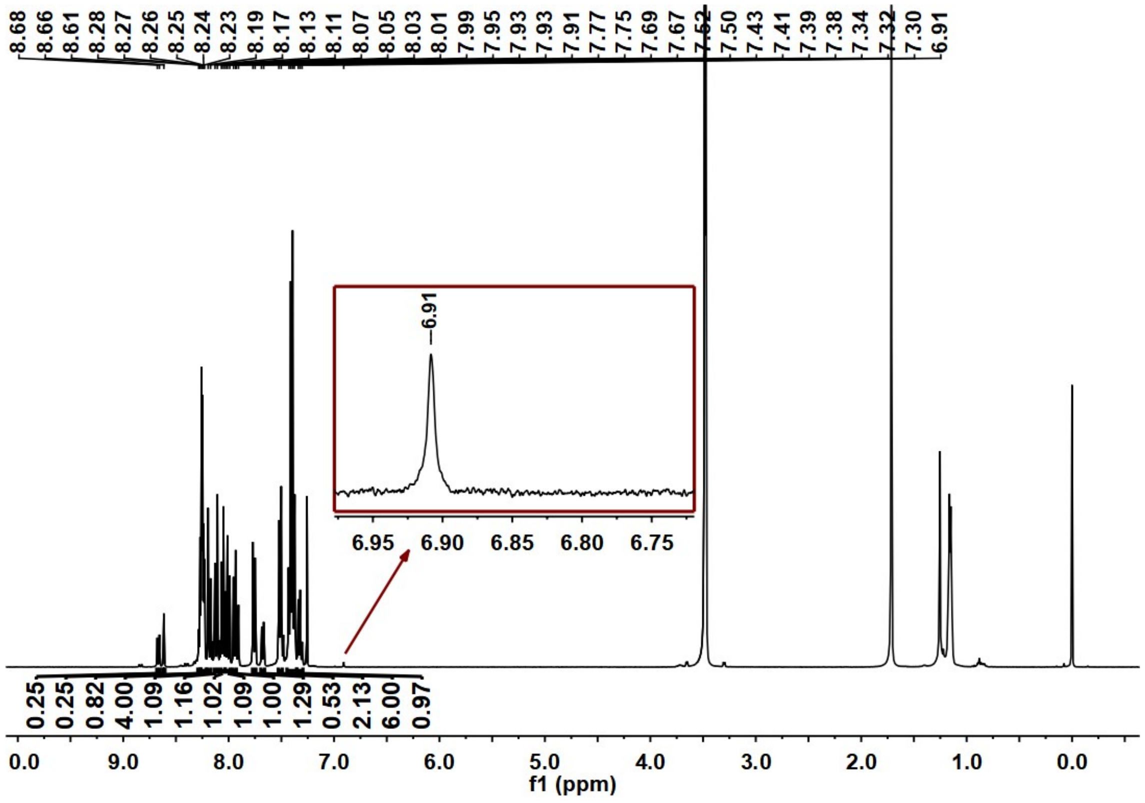

Figure S16. ^{1}H NMR spectra (400 Hz, $CDCl_3$) of **Py-Ph** after UV (365 nm) irradiation 3h

3. High-resolution mass spectrometry (HRMS)

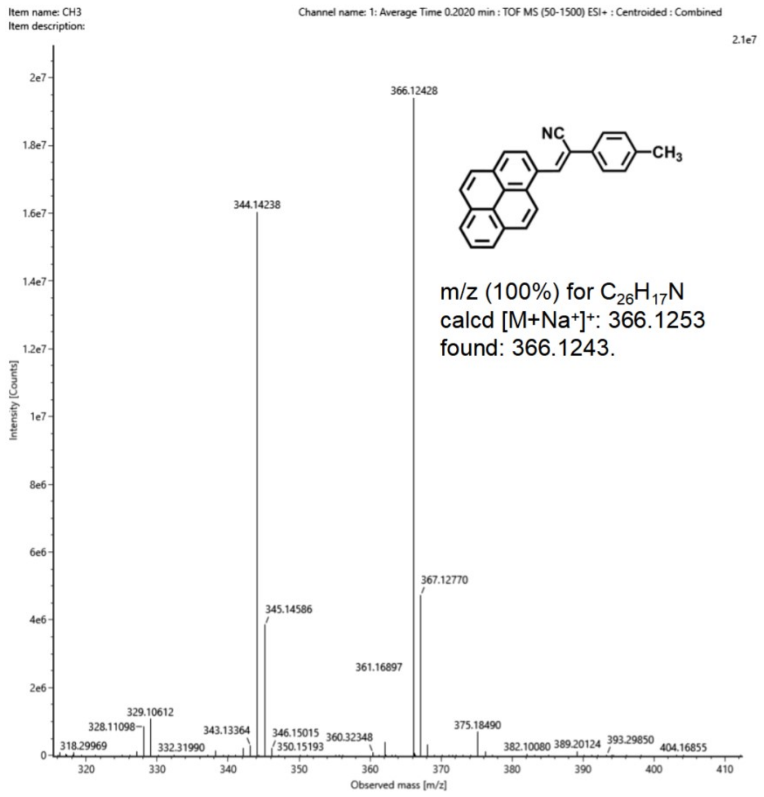

Figure S17. HRMS of **Py-CH$_3$**.

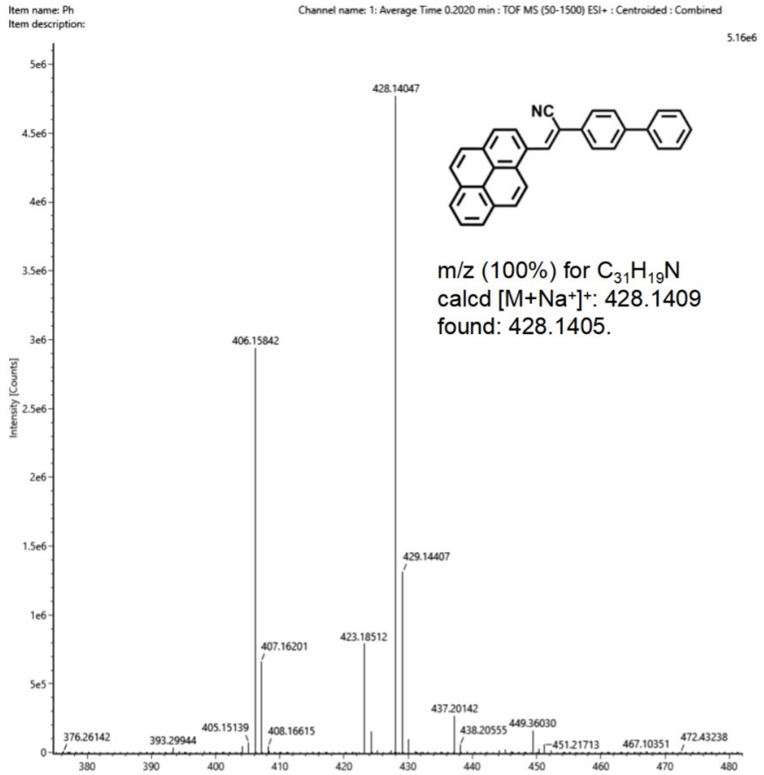

Figure S18. HRMS of **Py-Ph**.

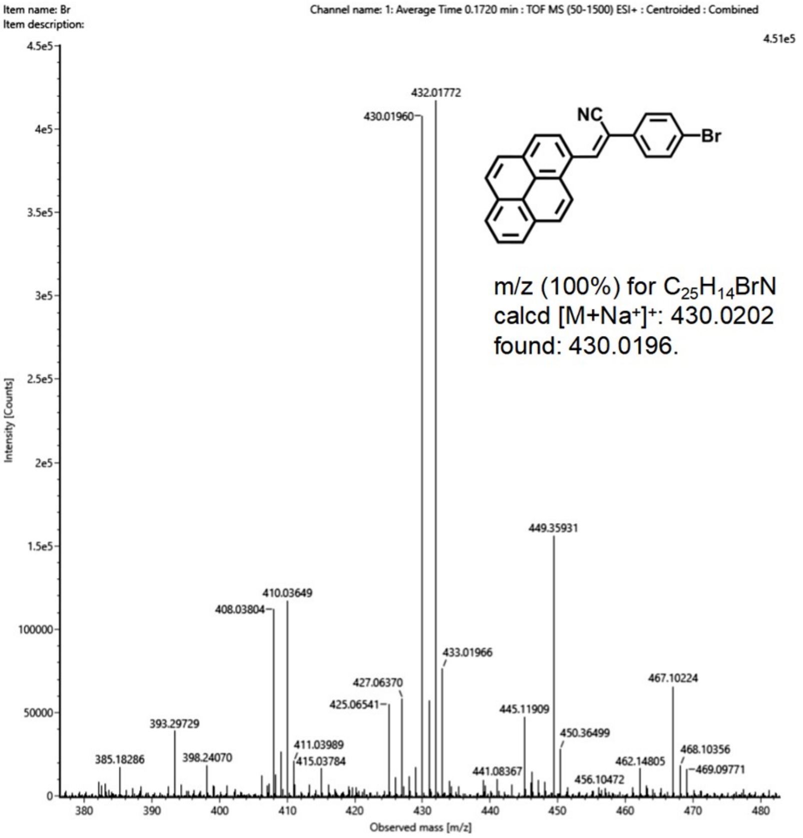

Figure S19. HRMS of **Py-Br**.

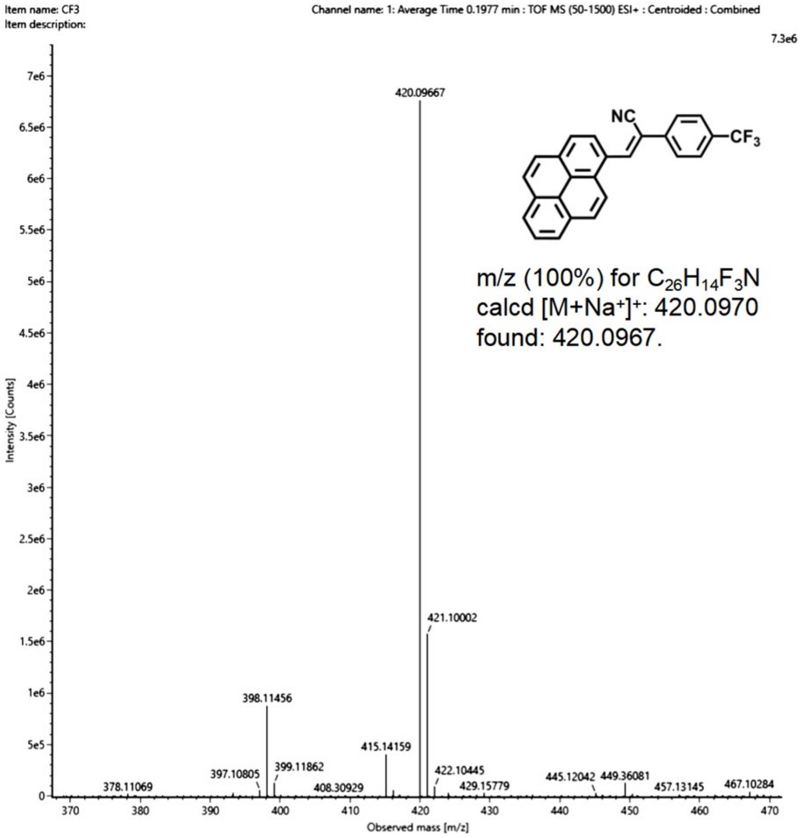

Figure S20. HRMS of **Py-CF$_3$**.

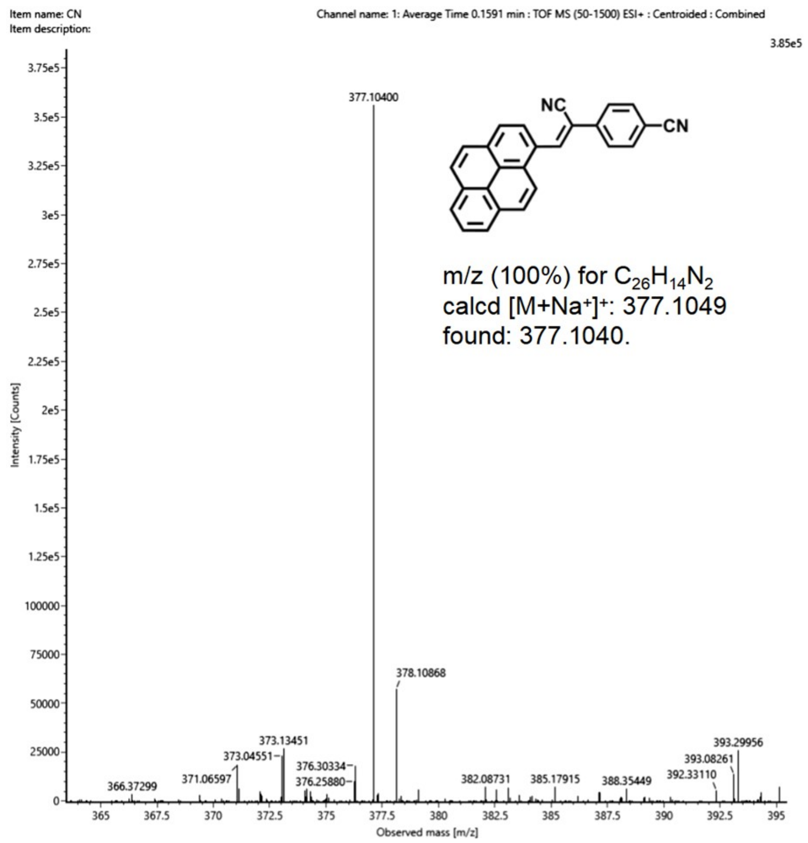

Figure S21. HRMS of **Py-CN**.

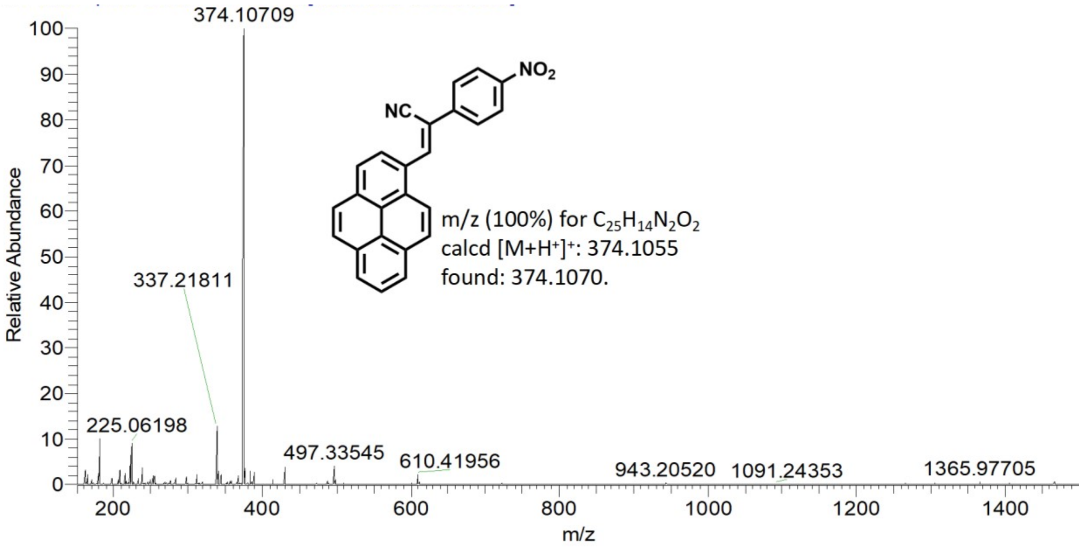

Figure S22. HRMS of **Py-NO$_2$**.

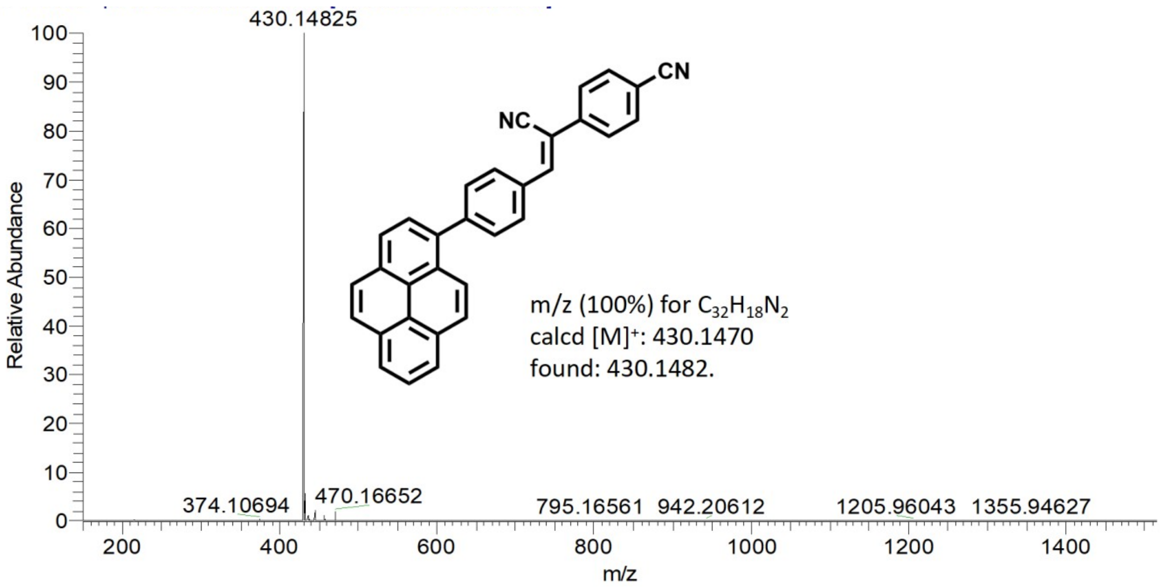

Figure S23. HRMS of **Py-PCN**.

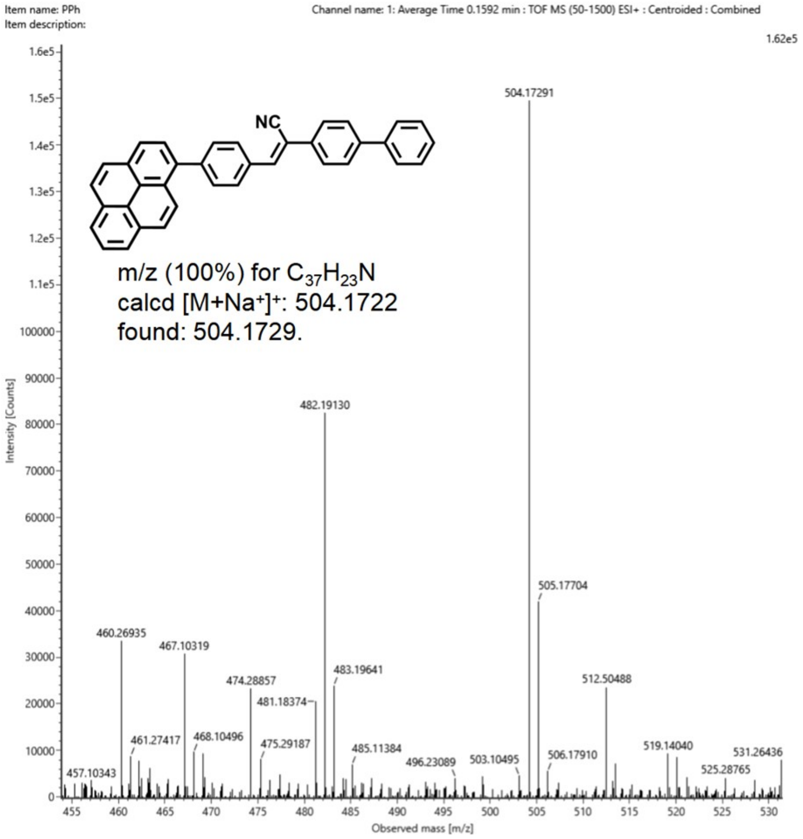

Figure S24. HRMS of **Py-PPh**.

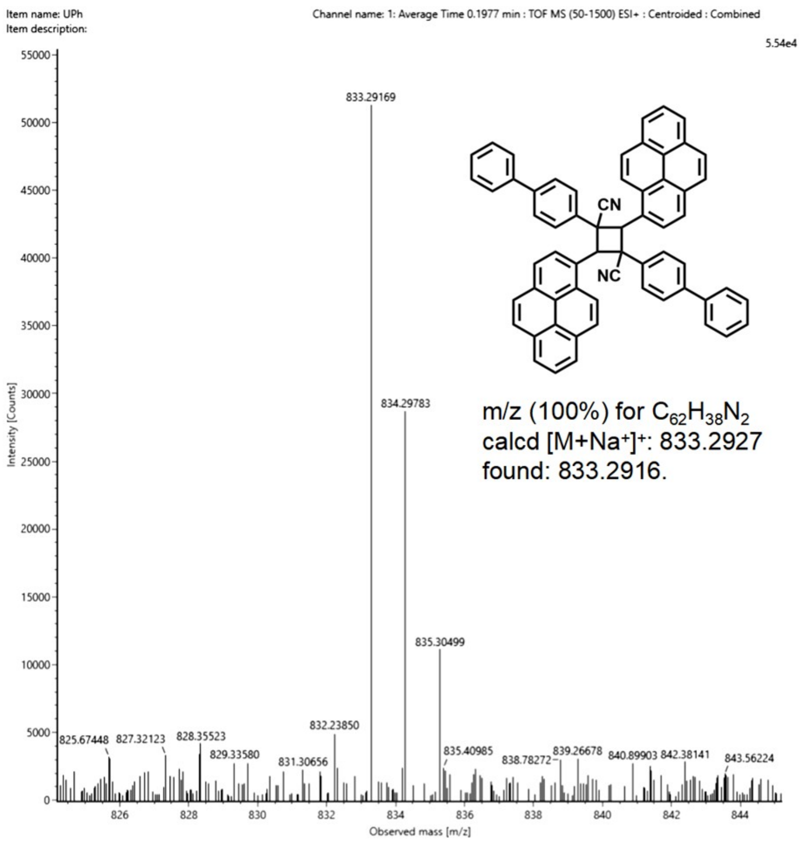

Figure S25. HRMS of **2@Py-Ph**.

4. Photophysical Properties

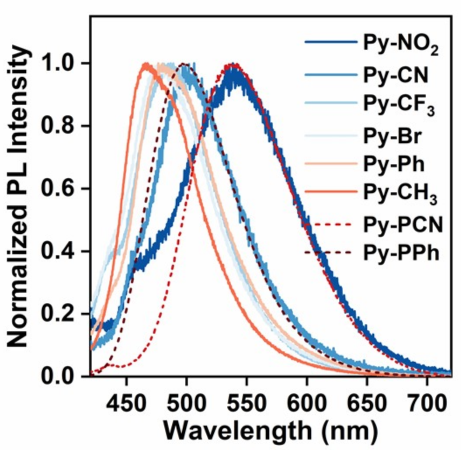

Figure S26. Emission spectra of **Py-R** and **Py-PR** in THF solution (10^{-5} M).

4.1 AIE properties

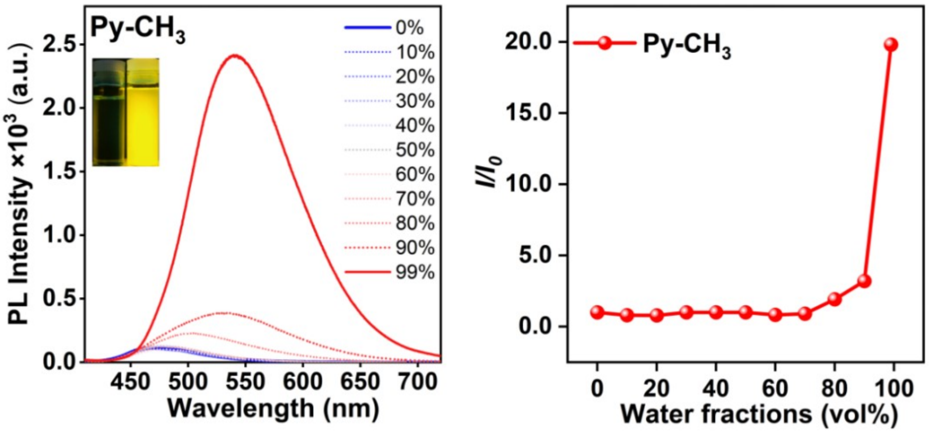

Figure S27. (Left): Emission spectra of **Py-CH₃** in the THF/H_2O with different f_w (10 μM). (Right): Plot of the relative PL intensity I/I_0 at different water fractions for **Py-CH₃**. Insert: Fluorescence images of **Py-CH₃** in f_w = 0% and 99% under UV irradiation (λ_{ex} = 365 nm)

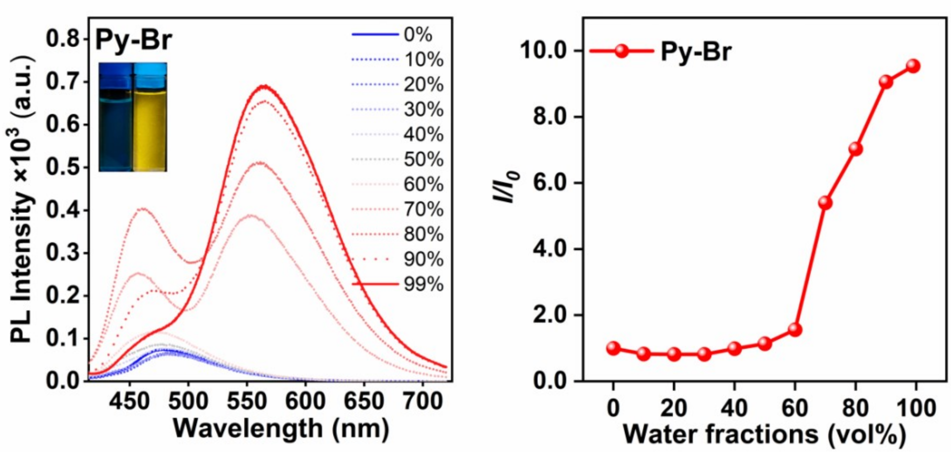

Figure S28. (Left): Emission spectra of **Py-Br** in the THF/H_2O with different f_w (10 μM). (Right): Plot of the relative PL intensity I/I_0 at different water fractions for **Py-Br**, (I is the maximum peak value of each curve). Insert: Fluorescence images of **Py-Br** in f_w = 0% and 99% under UV irradiation (λ_{ex} = 365 nm).

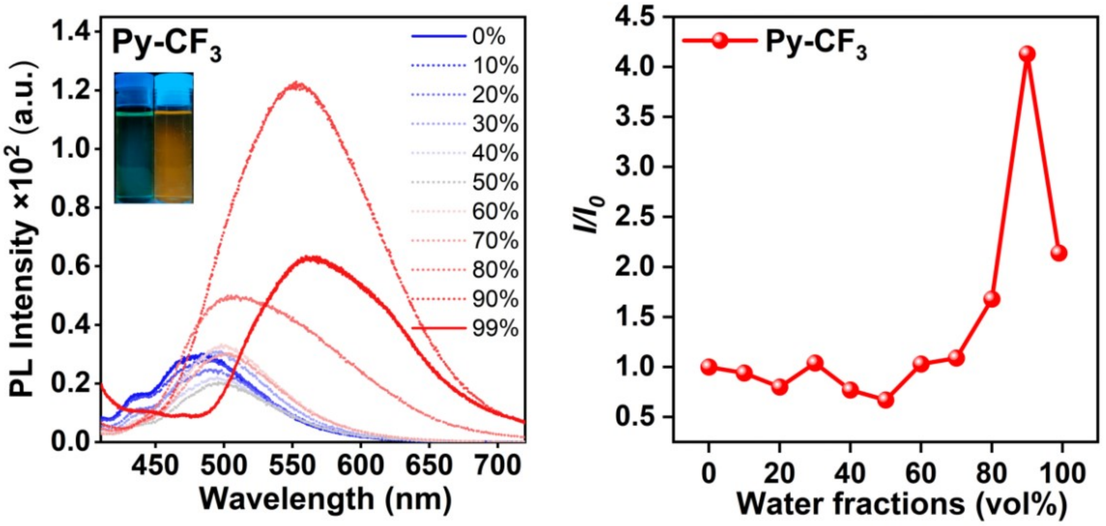

Figure S29. (Left): Emission spectra of **Py-CF$_3$** in the THF/H_2O with different f_w (10 μM). (Right): Plot of the relative PL intensity I/I_0 at different water fractions for **Py-CF$_3$**, (I is the maximum peak value of each curve). Insert: Fluorescence images of **Py-CF$_3$** in f_w = 0% and 99% under UV irradiation (λ_{ex} = 365 nm).

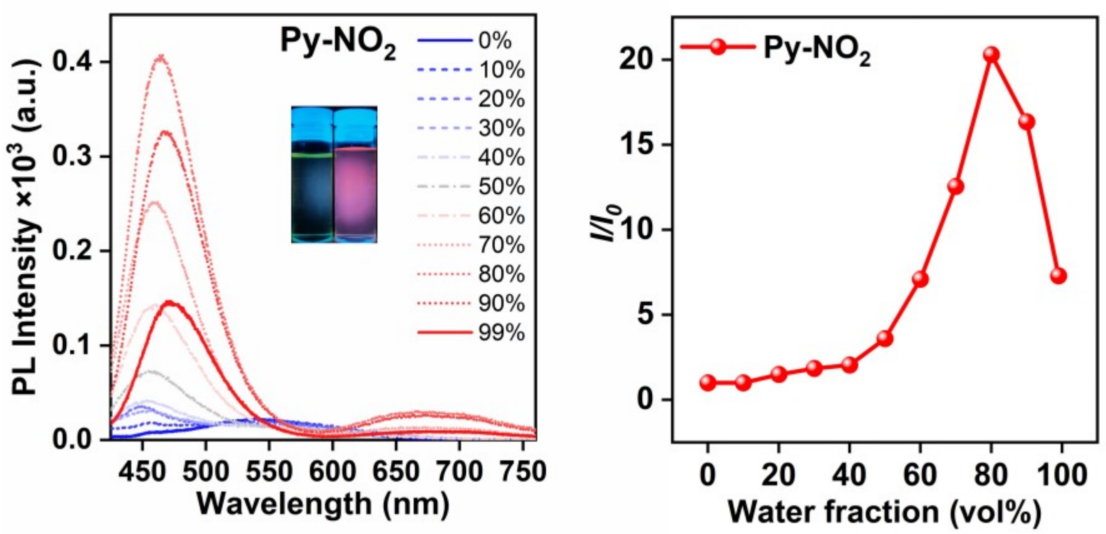

Figure S30. (Left): Emission spectra of **Py-NO$_2$** in the THF/H_2O with different f_w (10 μM). (Right): Plot of the relative PL intensity I/I_0 at different water fractions for **Py-NO$_2$**, (I is the maximum peak value of each curve). Insert: Fluorescence images of **Py-NO$_2$** in f_w = 0% and 99% under UV irradiation (λ_{ex} = 365 nm).

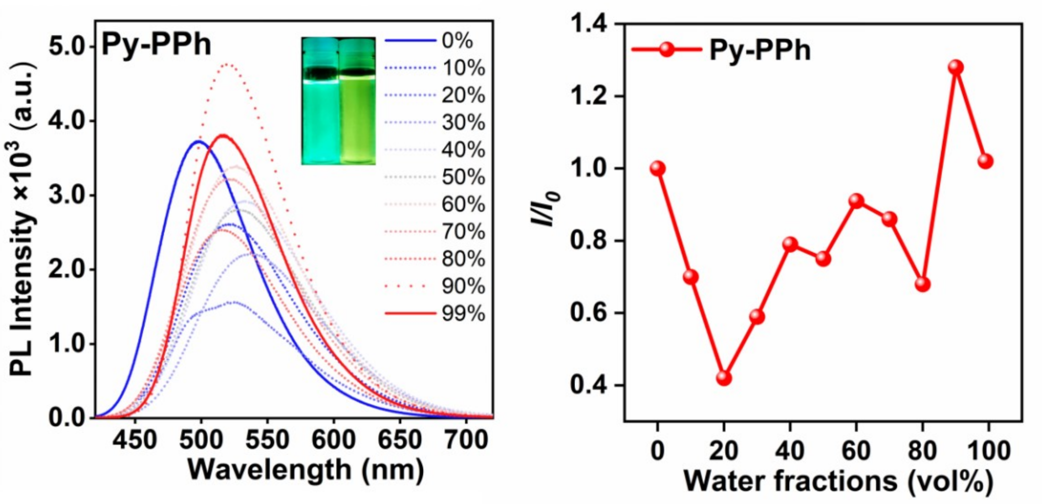

Figure S31. (Left): Emission spectra of **Py-PPh** in the THF/H_2O with different f_w (10 μM). (Right): Plot of the relative PL intensity I/I_0 at different water fractions for **Py-PPh**. Insert: Fluorescence images of **Py-PPh** in f_w = 0% and 99% under UV irradiation (λ_{ex} = 365 nm).

4.2 Solvent effect

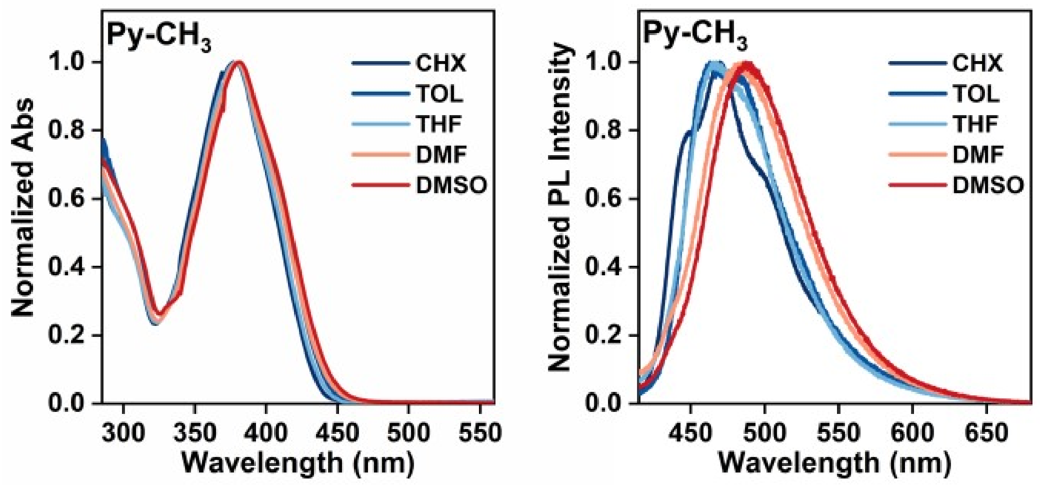

Figure S32. (Left) UV-Vis and (Right) emission spectra of **Py-CH$_3$** in various solvents.

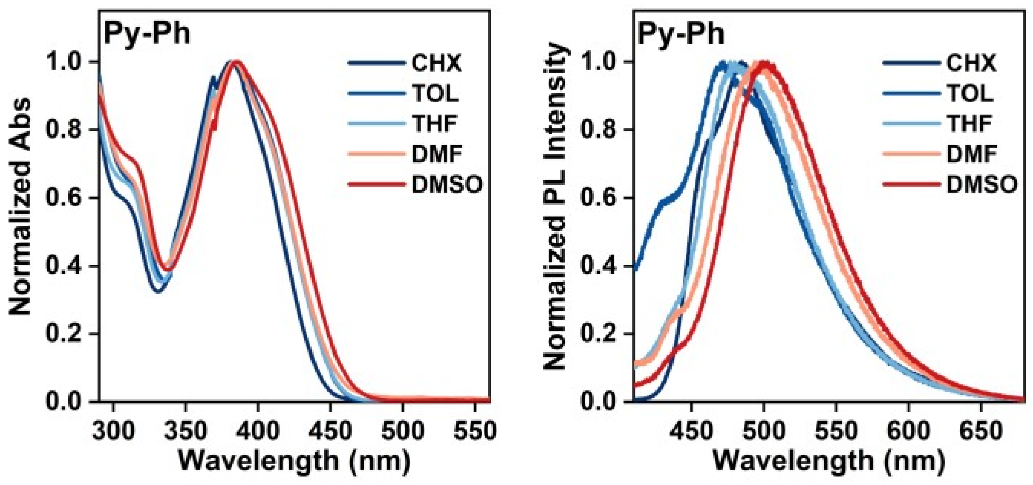

Figure S33. (Left) UV-Vis and (Right) emission spectra of **Py-Ph** in various solvents.

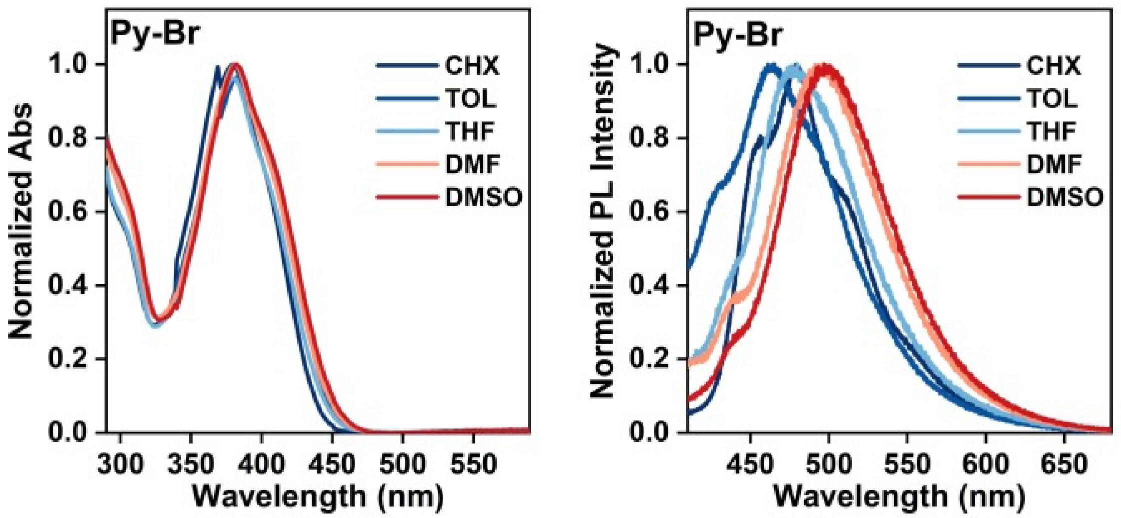

Figure S34. (Left) UV-Vis and (Right) emission spectra of **Py-Br** in various solvents.

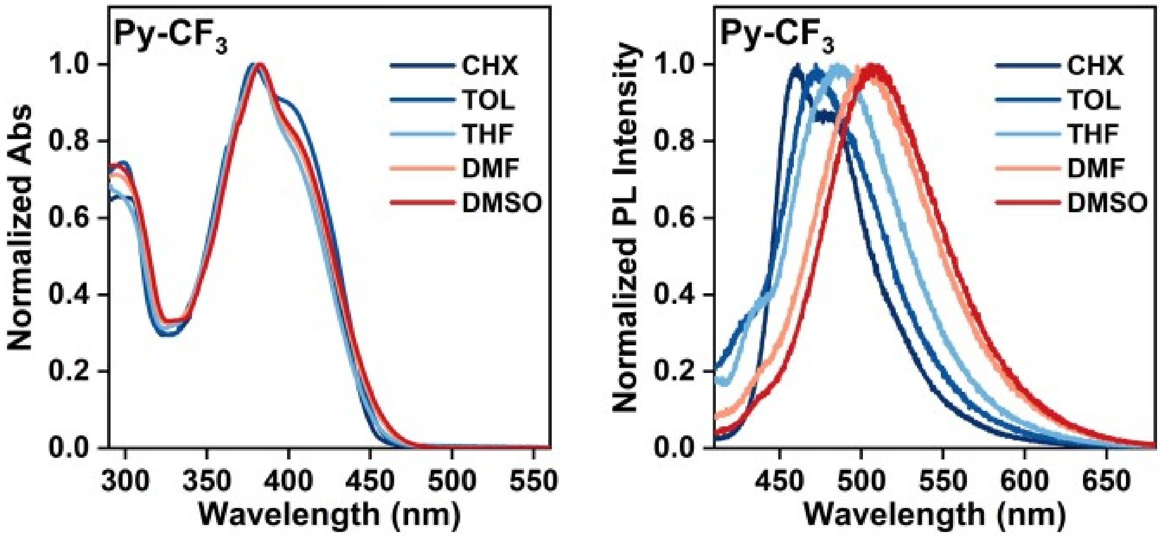

Figure S35. (Left) UV-Vis and (Right) emission spectra of **Py-CF$_3$** in various solvents.

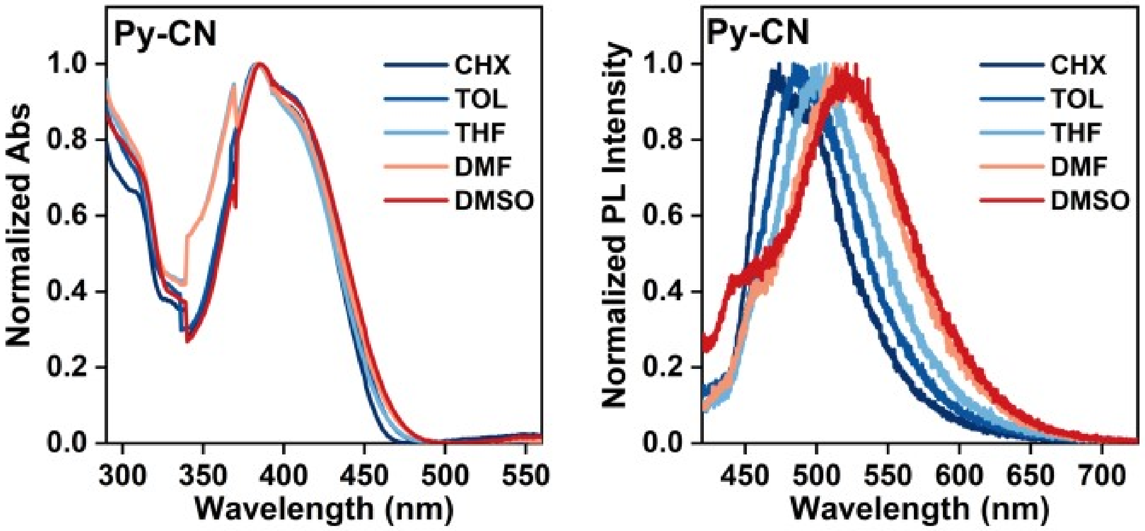

Figure S36. (Left) UV-Vis and (Right) emission spectra of **Py-CN** in various solvents.

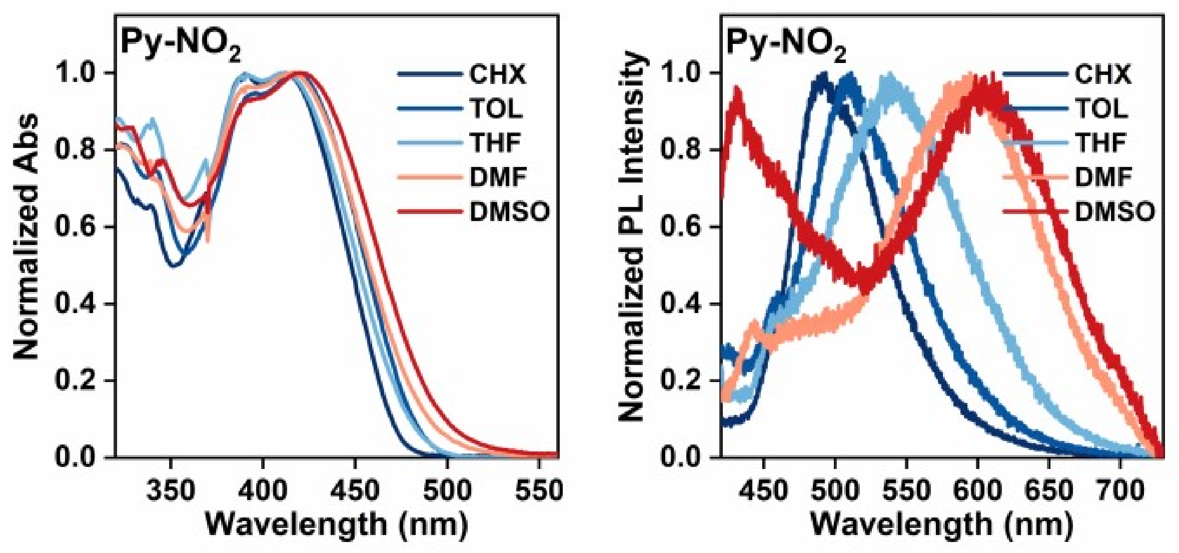

Figure S37. (Left) UV-Vis and (Right) emission spectra of **Py-NO$_2$** in various solvents.

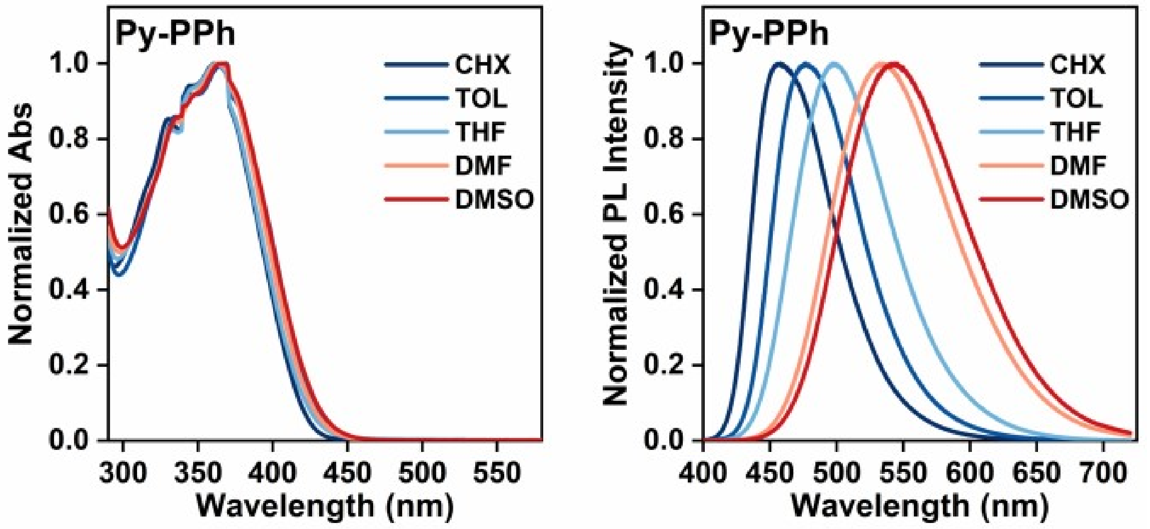

Figure S38. (Left) UV-Vis and (Right) emission spectra of **Py-PPh** in various solvents.

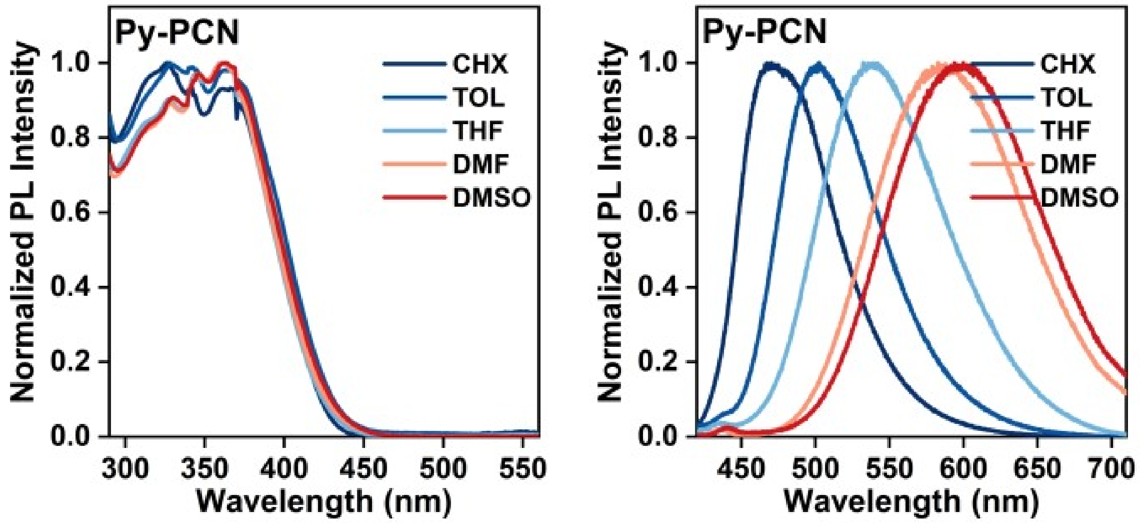

Figure S39. (Left) UV-Vis and (Right) emission spectra of **Py-PCN** in various solvents.

4.3 Time-resolved fluorescence spectra

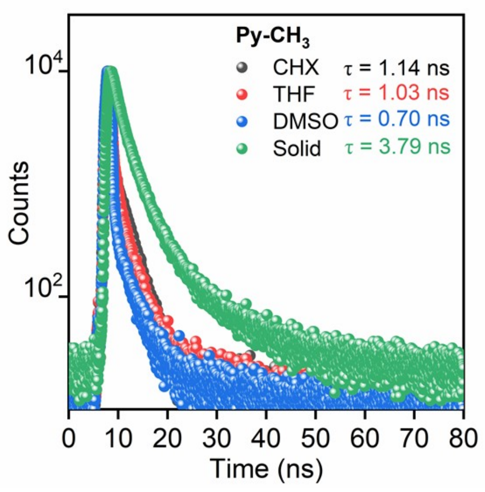

Figure S40. Time-resolved fluorescence spectra of **Py-CH$_3$** in different solvents and in the solid state.

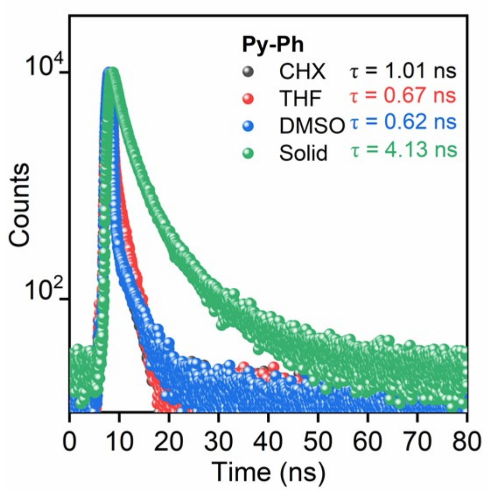

Figure S41. Time-resolved fluorescence spectra of **Py-Ph** in different solvents and in the solid state.

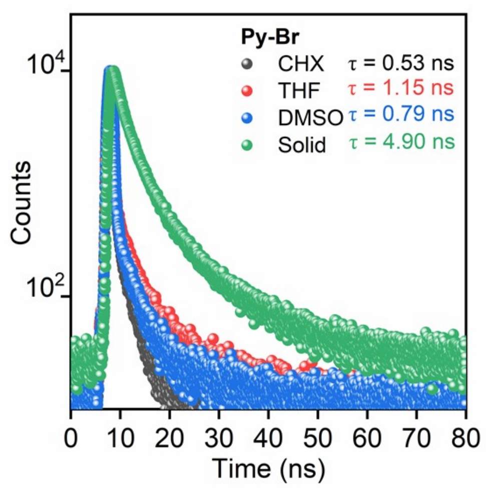

Figure S42. Time-resolved fluorescence spectra of **Py-Ph** in different solvents and in the solid state.

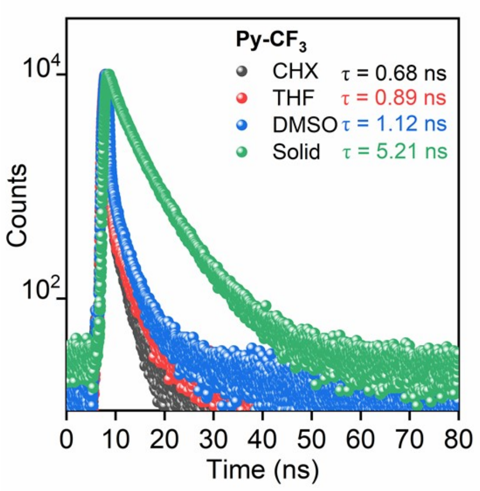

Figure S43. Time-resolved fluorescence spectra of **Py-CF$_3$** in different solvents and in the solid state.

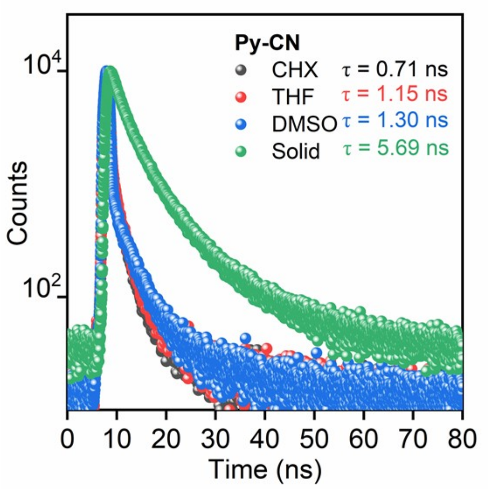

Figure S44. Time-resolved fluorescence spectra of **Py-CN** in different solvents and in the solid state.

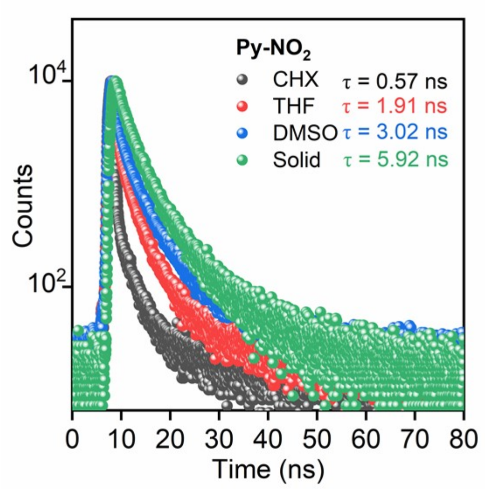

Figure S45. Time-resolved fluorescence spectra of **Py-NO$_2$** in different solvents and in the solid state.

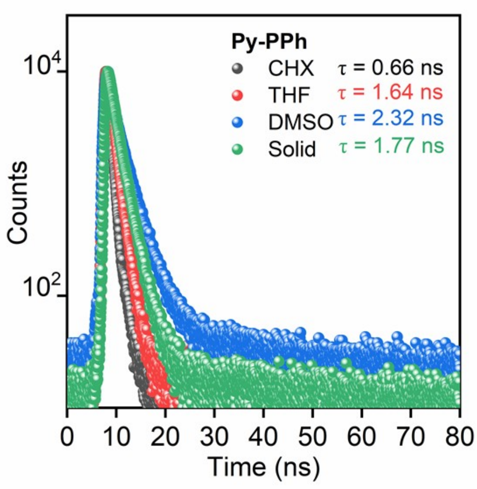

Figure S46. Time-resolved fluorescence spectra of **Py-PPh** in different solvents and in the solid state.

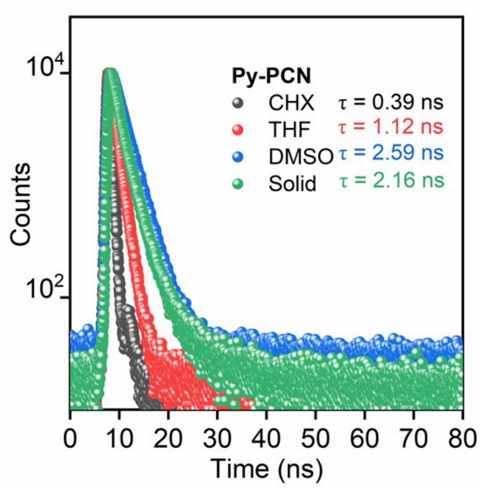

Figure S47. Time-resolved fluorescence spectra of **Py-PCN** in different solvents and in the solid state.

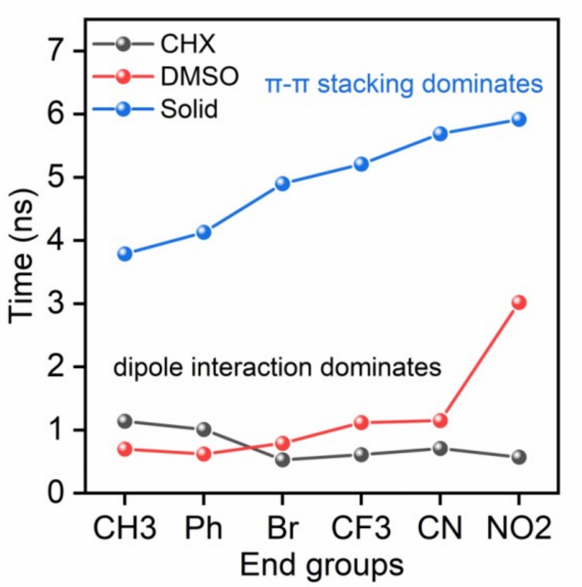

Figure S48. The change curve of end groups-dependent of fluorescence lifetime.

4.4 Concentration-dependent emission spectra

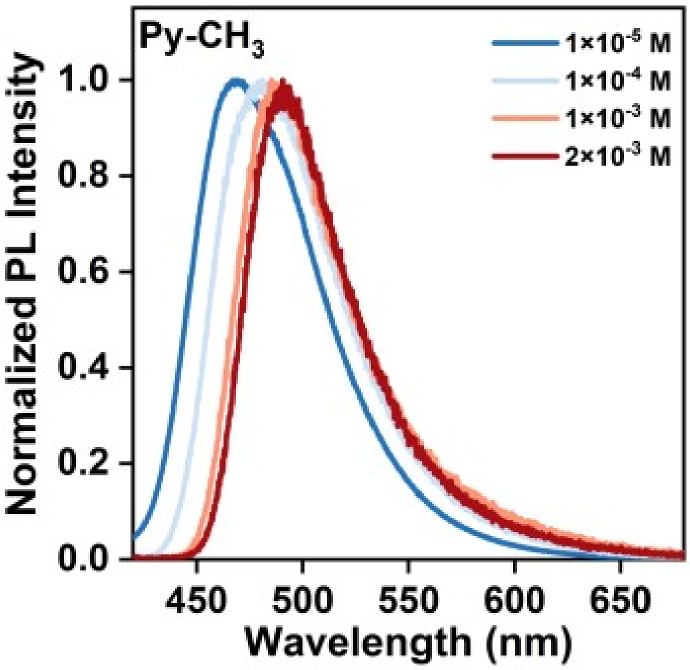

Figure S49. Emission spectra of **Py-CH$_3$** in THF at different concentrations.

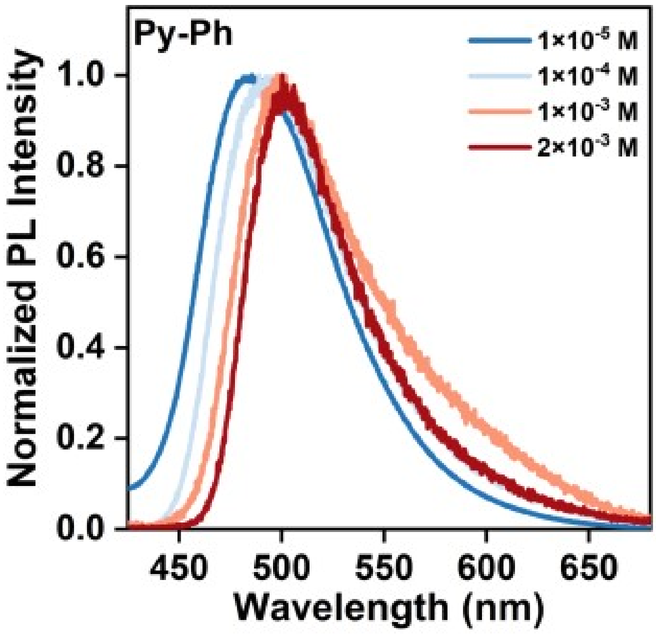

Figure S50. Emission spectra of **Py-Ph** in THF at different concentrations.

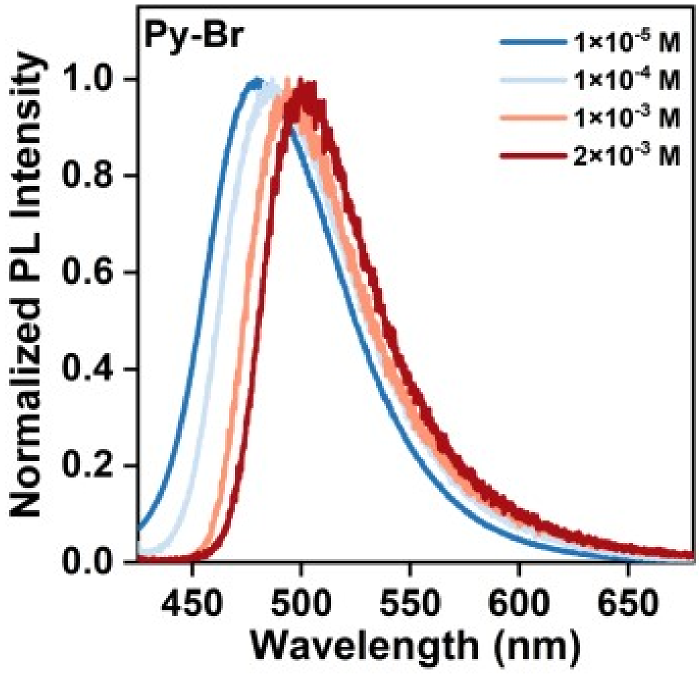

Figure S51. Emission spectra of **Py-Br** in THF at different concentrations.

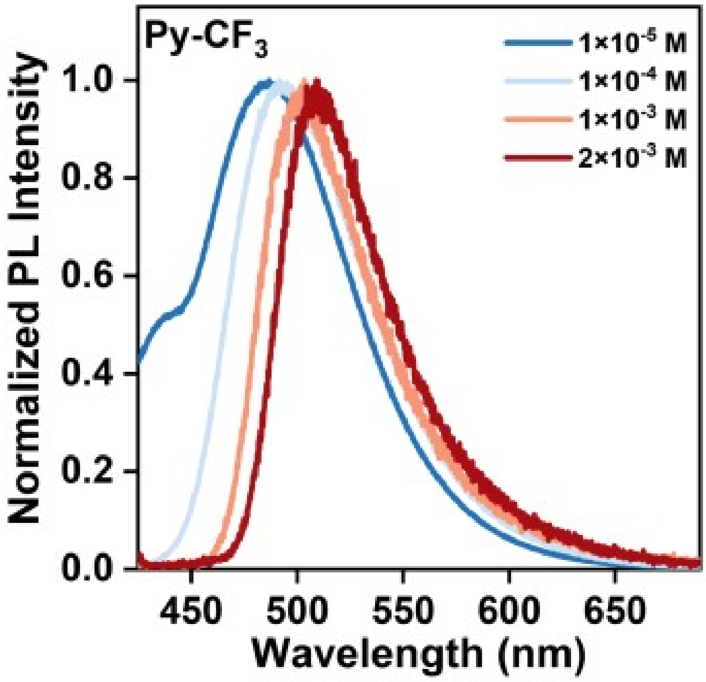

Figure S52. Emission spectra of **Py-CF$_3$** in THF at different concentrations.

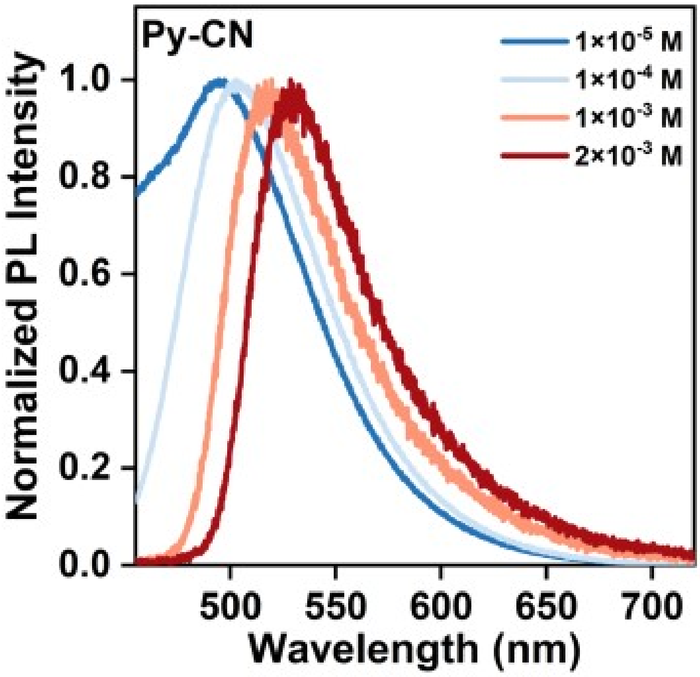

Figure S53. Emission spectra of **Py-CN** in THF at different concentrations.

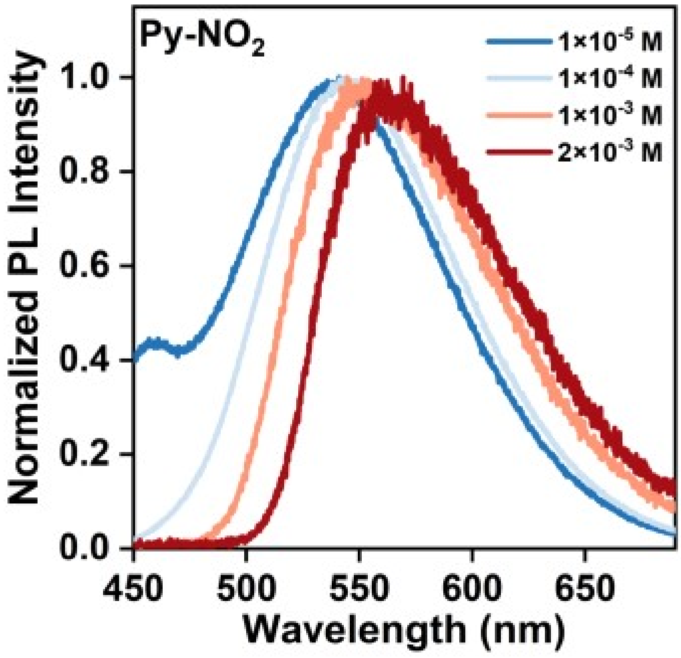

Figure S54. Emission spectra of **Py-NO$_2$** in THF at different concentrations.

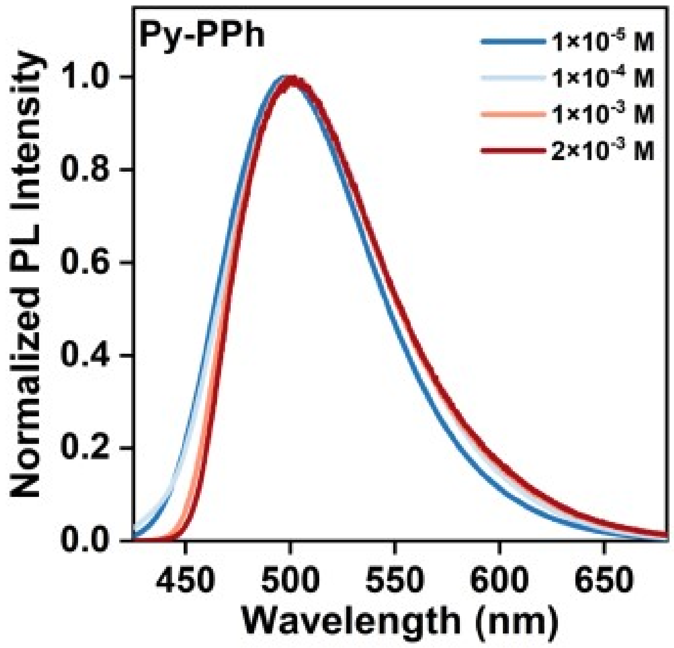

Figure S55. Emission spectra of **Py-PPh** in THF at different concentrations.

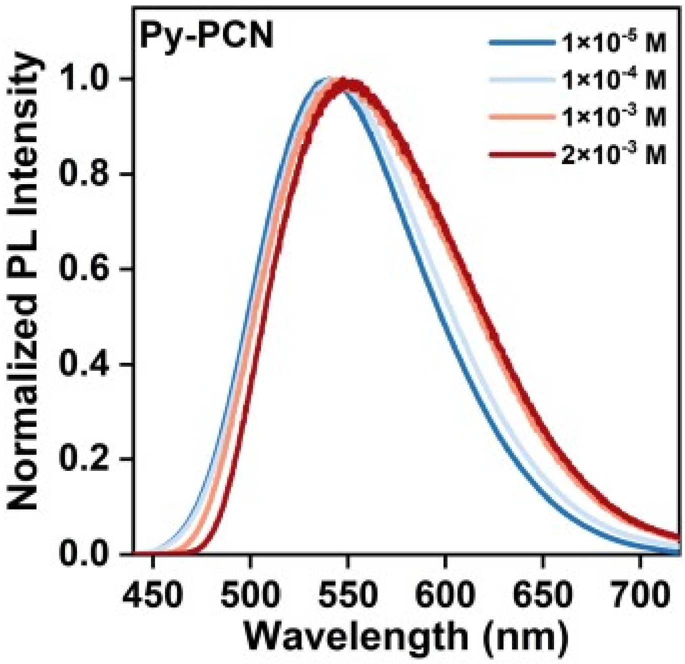

Figure S56. Emission spectra of **Py-PCN** in THF at different concentrations.

4.5 Viscosity-dependent emission spectra

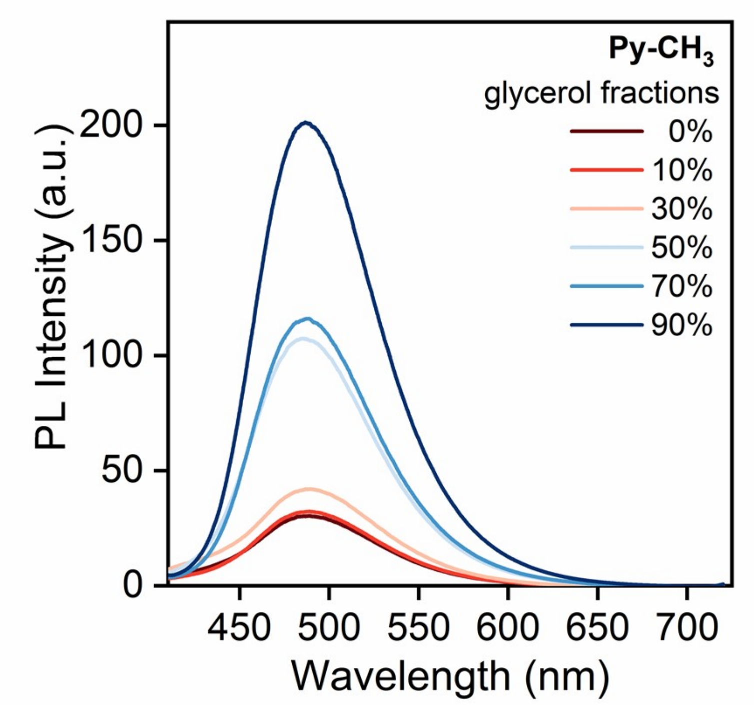

Figure S57. Emission spectra of **Py-CH$_3$** in DMSO/glycerol with different glycerol fractions.

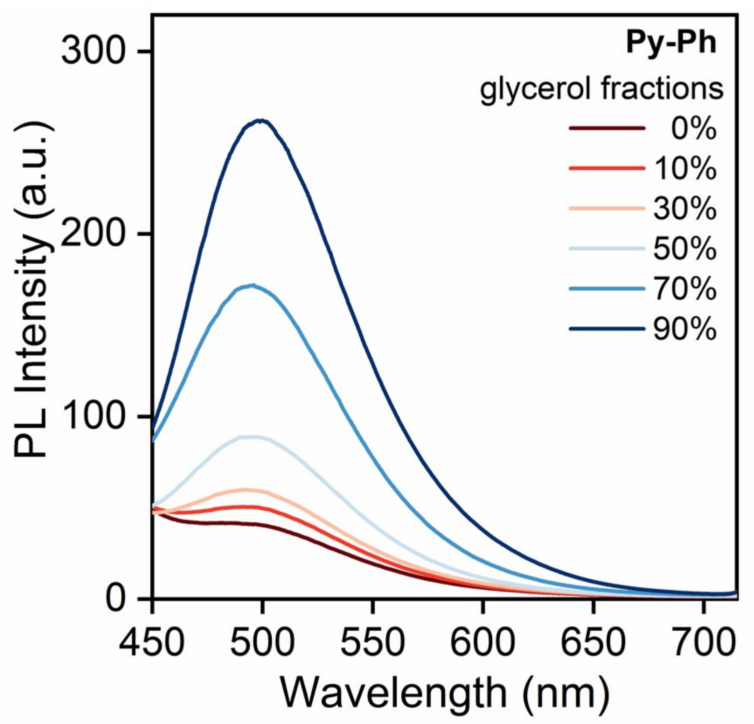

Figure S58. Emission spectra of **Py-Ph** in DMSO/glycerol with different glycerol fractions.

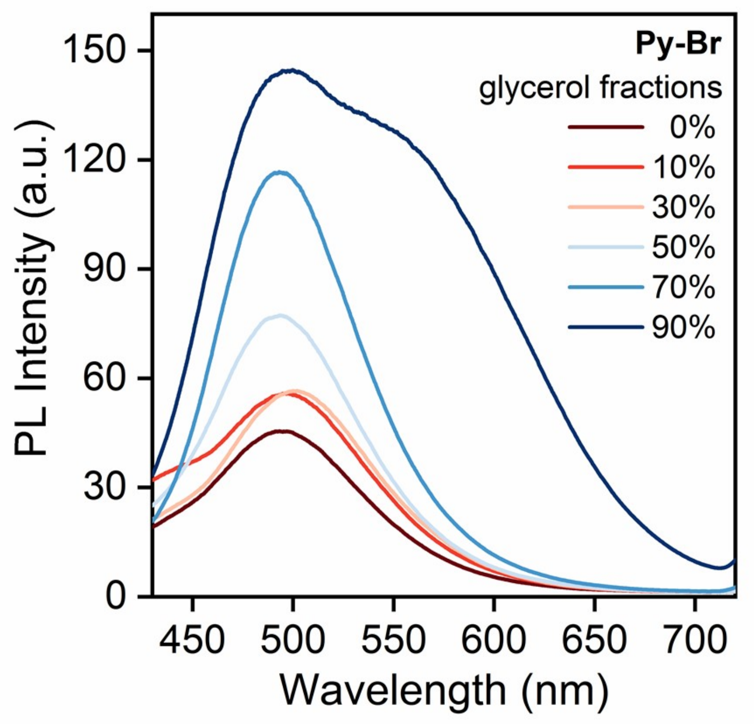

Figure S59. Emission spectra of **Py-Br** in DMSO/glycerol with different glycerol fractions.

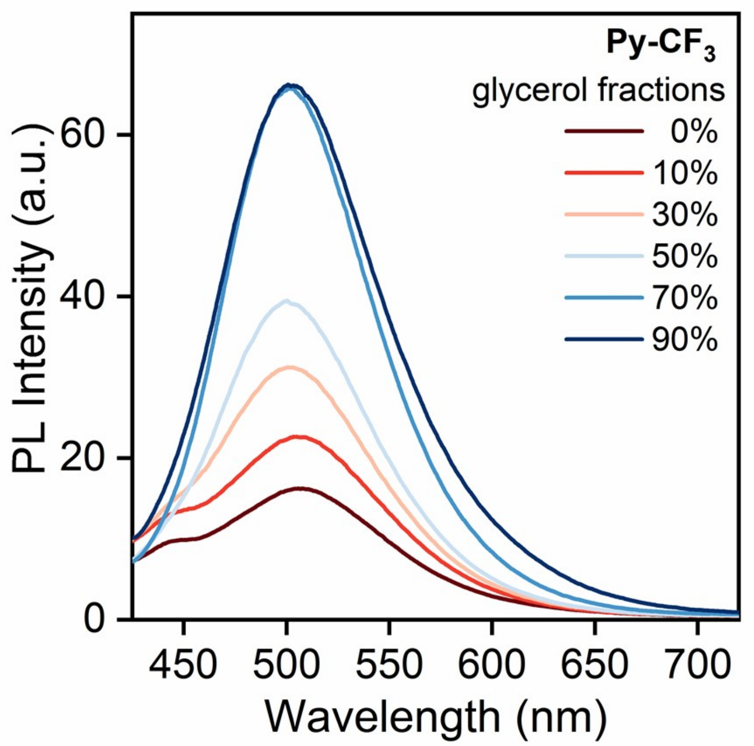

Figure S60. Emission spectra of **Py-CF_3** in DMSO/glycerol with different glycerol fractions.

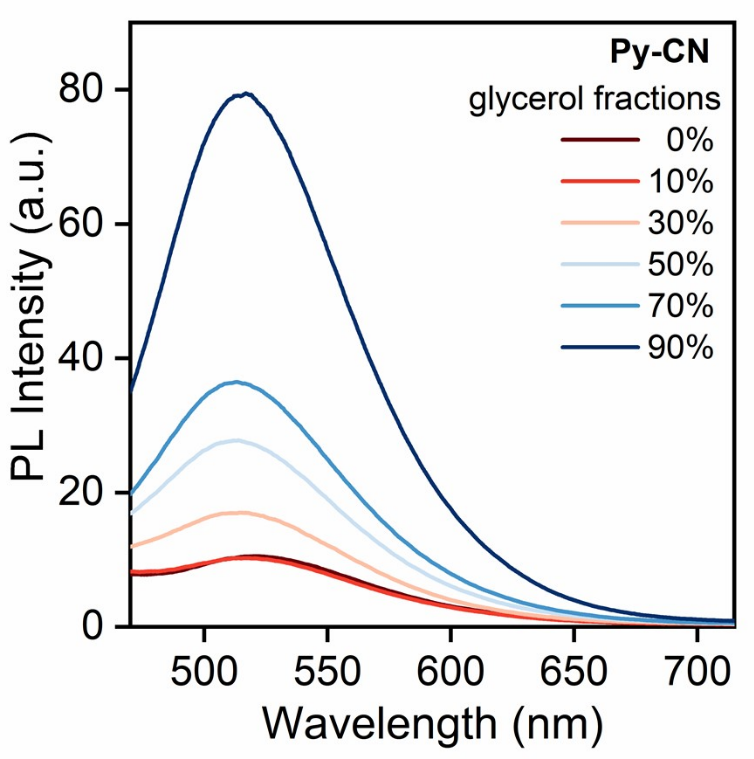

Figure S61. Emission spectra of **Py-CN** in DMSO/glycerol with different glycerol fractions.

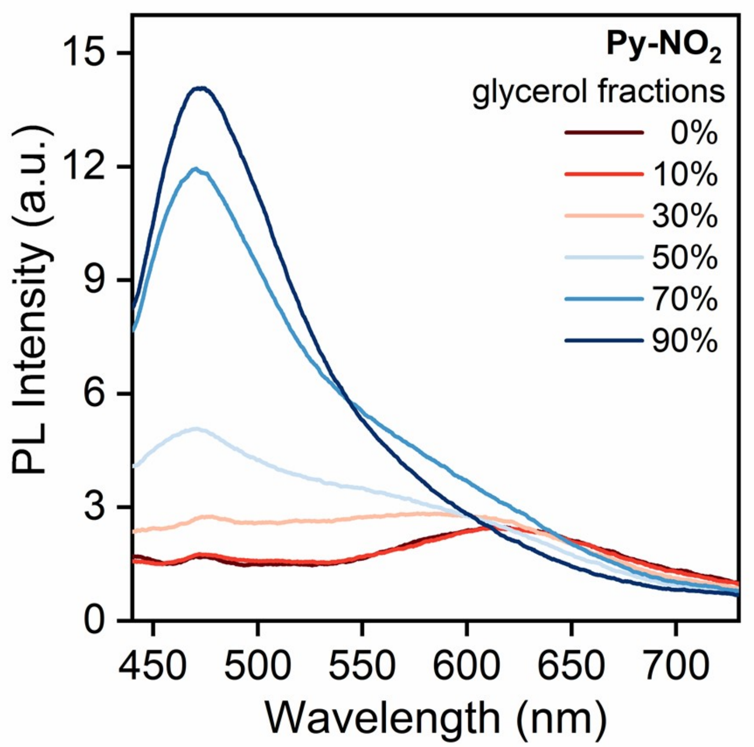

Figure S62. Emission spectra of **Py-NO_2** in DMSO/glycerol with different glycerol fractions.

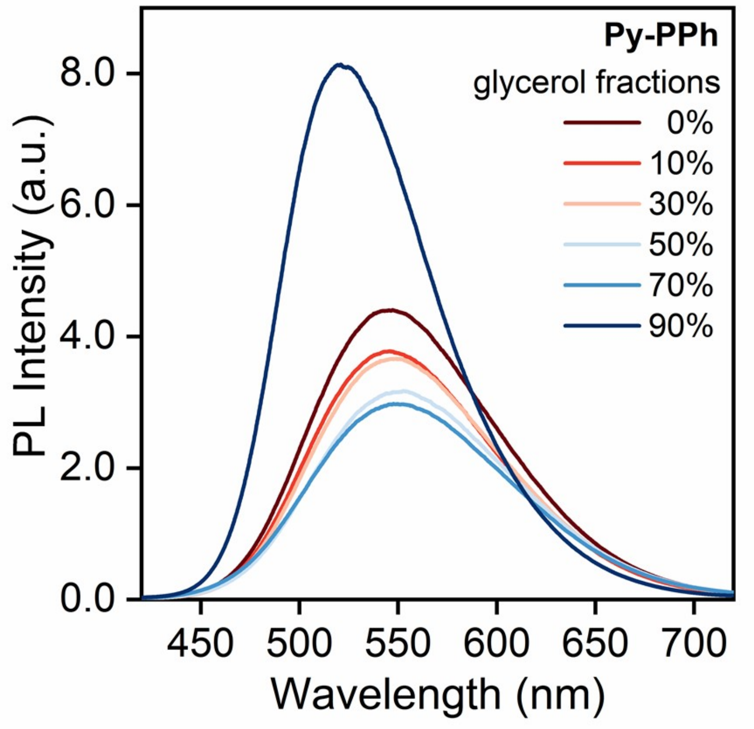

Figure S63. Emission spectra of **Py-PPh** in DMSO/glycerol with different glycerol fractions.

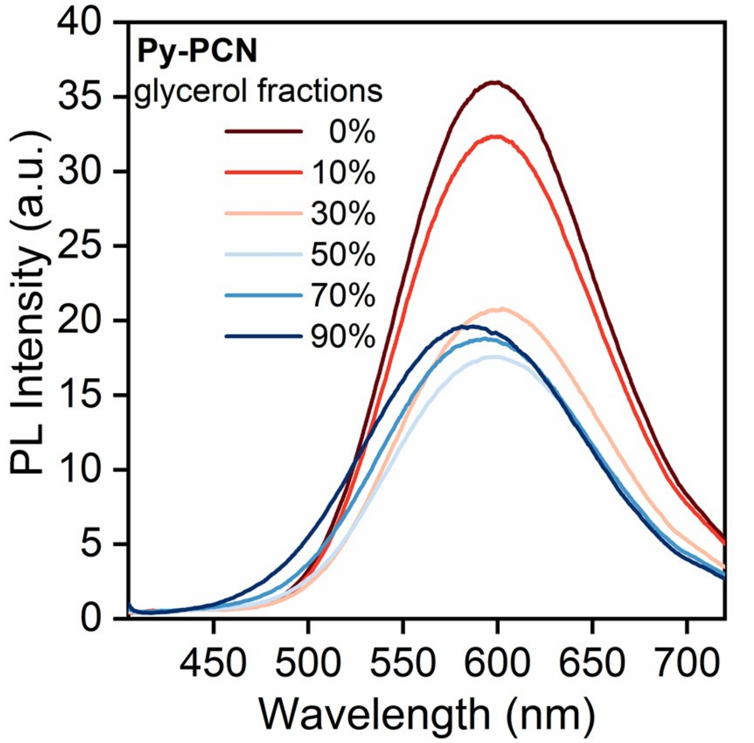

Figure S64. Emission spectra of **Py-PCN** in DMSO/glycerol with different glycerol fractions.

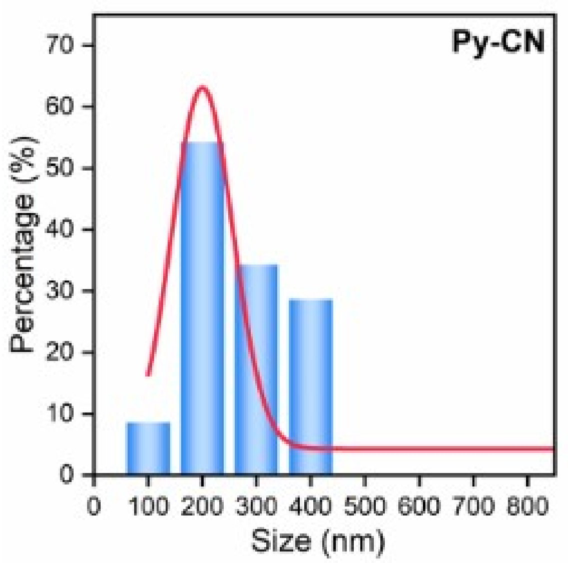

Figure S65. The particle size distribution of **Py-CN** calculated by Nano measured according to their SEM photos.

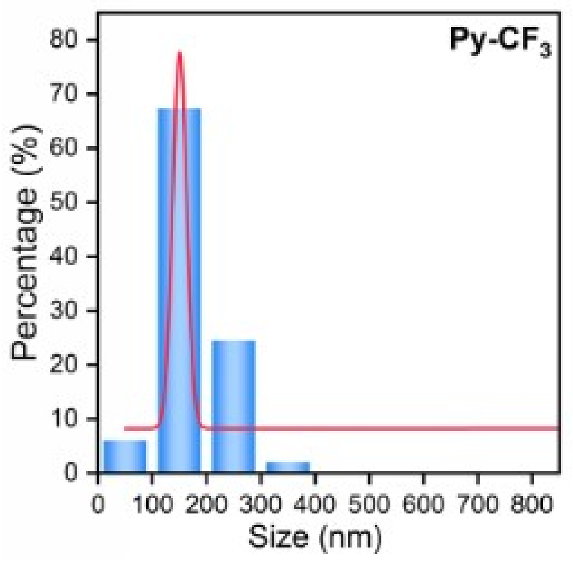

Figure S66. The particle size distribution of **Py-CF_3** calculated by Nano measured according to their SEM photos.

5. X-ray single crystal diffraction analysis

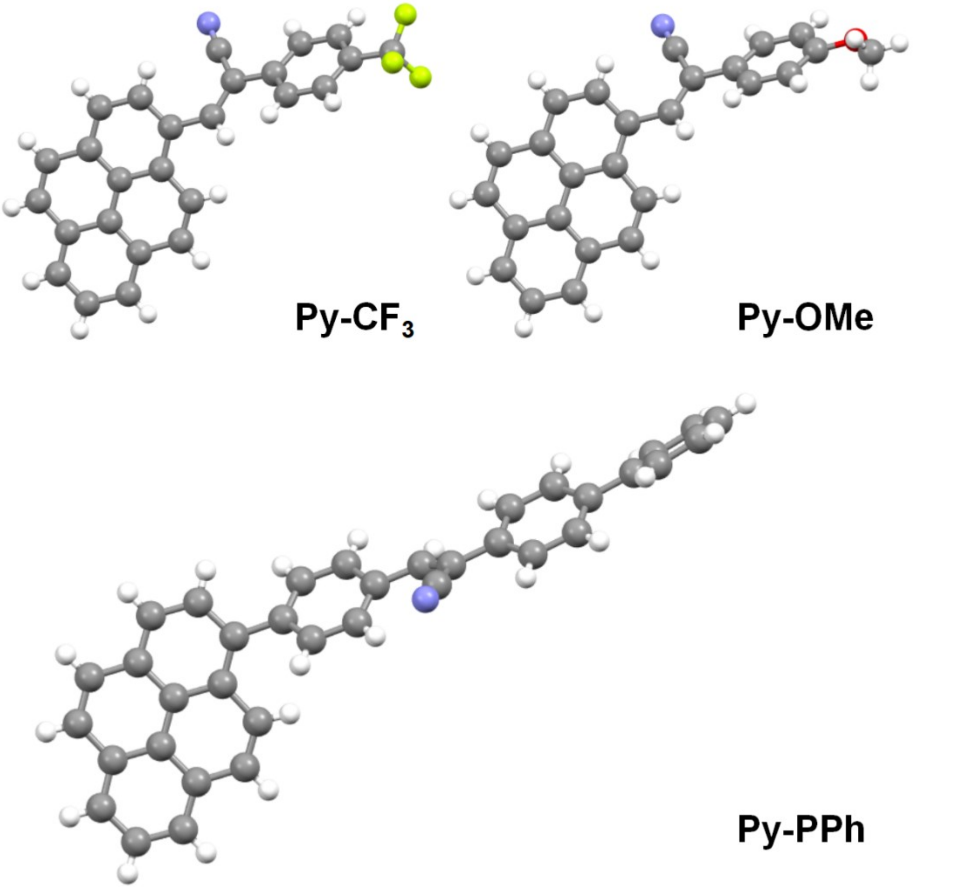

Figure S67. Single crystal structures of **Py-CF$_3$**, **Py-OMe** [1] and **Py-PPh**

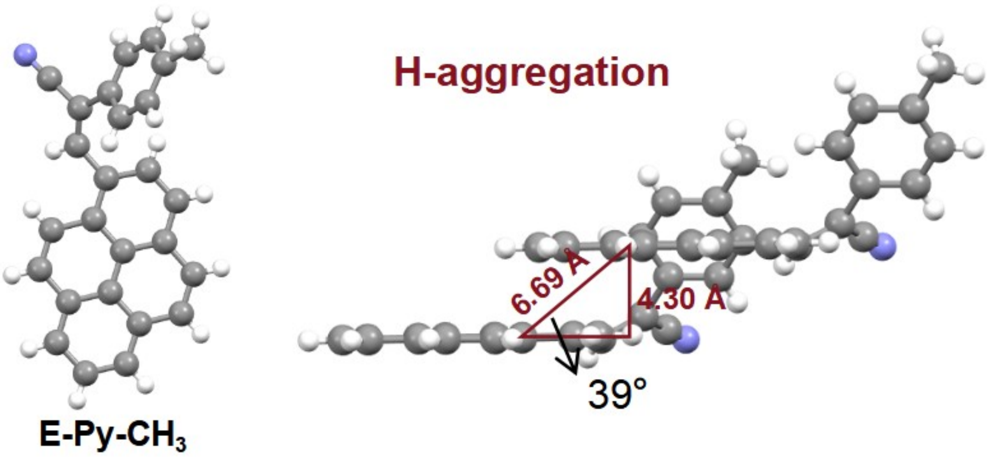

Figure S68. Single crystal structure and packing arrangement of **E-Py-CH$_3$**.

[1] J. Katla, H. R. Bhat, P. C. Jha, P.S. Ghalsasi, and S. Kanvah, *ChemistrySelect* **2017**, *2*, 1902.

Table S1. Key crystal parameters of compounds **Py-CF$_3$, Py-PPh** and **(*E*)-Py-CH$_3$**.

Compound	**Py-CF$_3$**	**Py-PPh**	***E*-Py-CH$_3$**
Empirical	2·$C_{36}H_{14}F_3N$	$C_{37}H_{23}N$	$C_{26}H_{17}N$
Formula weight	794.76	481.56	343.40
Crystal system	Monoclinic	Orthorhombic	Monoclinic
Space group	$P2_1/c$	$Pna2_1$	Pc
a[Å]	8.102(2)	7.5759(2)	7.9507(2)
b[Å]	34.566(12)	14.0073(6)	6.6959(3)
c[Å]	13.851(5)	23.4629(6)	16.8907(6)
α[°]	90	90	90
β[°]	100.625(11)	90	95.0100(10)
γ[°]	90	90	90
Volume[Å^3]	3813(2)	2489.83(14)	895.78(6)
F(000)	1632.0	1008	360.0
Z	4	4	2
Dcalcd[Mg/m^3]	1.385	1.285	1.273
Measured reflns	28890	56783	8121
unique reflns	6698	7290	3983
parameters	-1.1(10)	-1.1(10)	2.5(10)
GOF on F^2	0.925	1.090	1.091
Largest diff map features /e Å^{-3}	0.39/-0.32	0.25/-0.21	0.29/-0.21

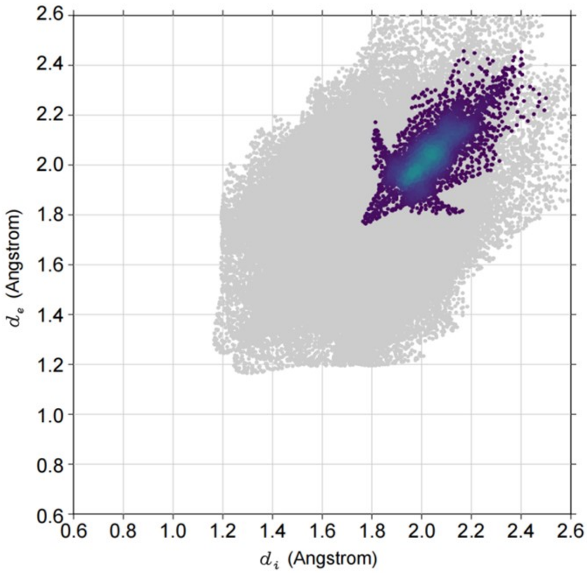

Figure S69. Hirshfeld surfaces and decomposed fingerprint plots of the intermolecular C-C interaction of **Py-PPh**

Table S2. The contribution of various atoms in intermolecular interactions

	**Py-CF$_3$**	**Py-OMe**	**Py-PPh**
H-H	26.80%	31.96%	42.66%
C-H	21.35%	50.27%	41.19%
N-H	9.22%	10.64%	8.08%
C-C	14.43%	1.01%	7.08%
C-N	0.39%	0.77%	0.99%
H-F	20.57%	---	---
F-F	6.07%	---	---
O-H	---	5.36%	---
C-F	0.26%	---	---
N-N	0.91%	---	---

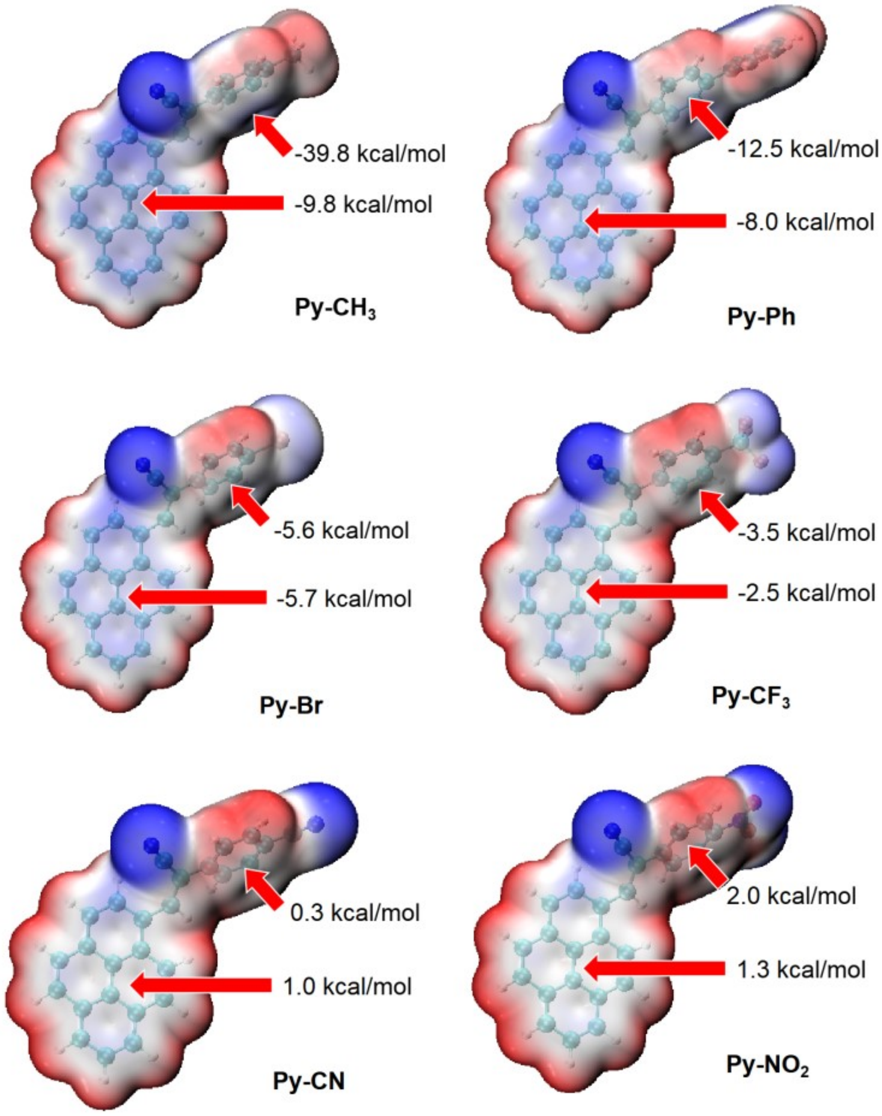

Figure S70. Molecular surface electrostatic potential of **Py-R**.

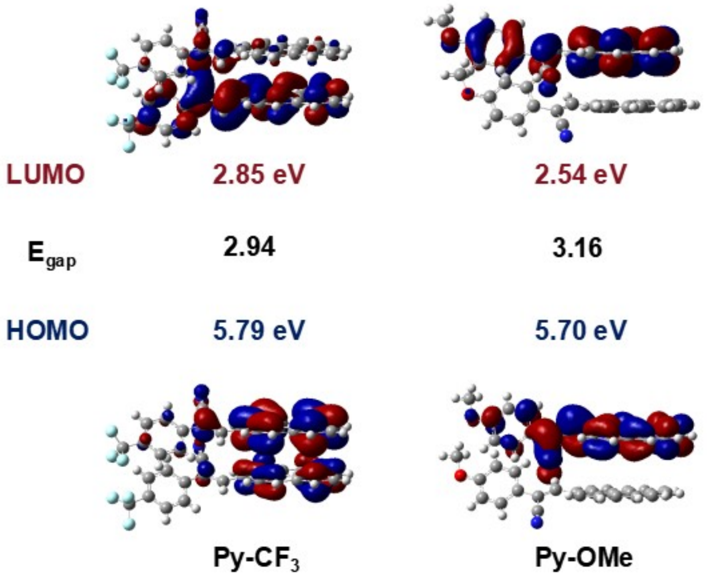

Figure S71. Frontline molecular orbital electron cloud distribution of dimers of **Py-CF$_3$** and **Py-OMe** (dimers are built from a single crystal packing modes) (B3LYP/6-31g$^+$ (d, p))

6. DFT calculation

6.1 Optimized conformation

Table S3. The optimized conformation of **Py-R** and **Py-PR** (B3LYP/6-31g^{+} (d, p))

Comps.	Top view	Side view
**Py-NO$_2$**	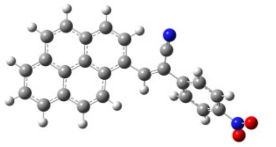	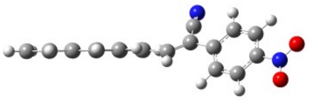
**Py-CN**	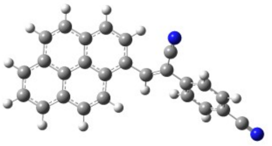	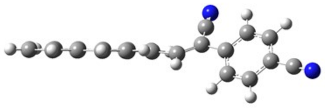
**Py-CF$_3$**	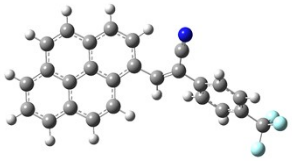	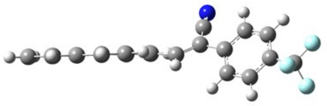
**Py-Br**	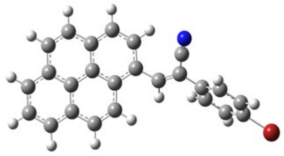	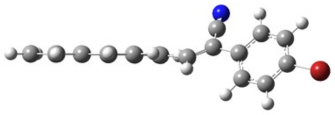
**Py-Ph**	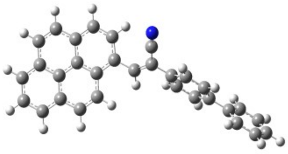	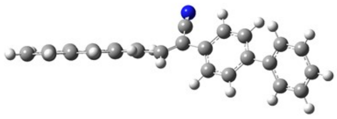
**Py-CH$_3$**	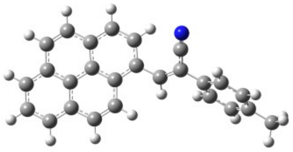	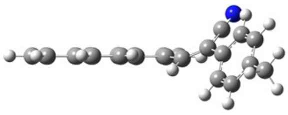
**Py-PPh**	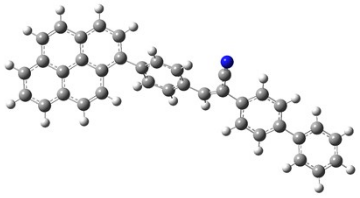	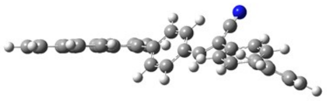
**Py-PCN**	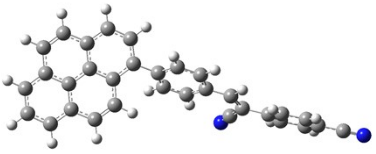	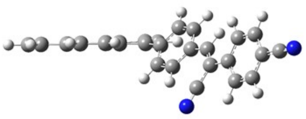

6.2 TD-DFT parameters

Table S4. The theoretical parameters of **Py-R** and **Py-PR** (B3LYP, 6-31g^{+} (d,p))

Sample	Experimental λ_{ab} (nm)	Theoretical λ_{ab} (nm)	Osc. strength	Major cocontributions (% coefficients)	energy gaps （eV）
**Py-NO$_2$**	410	415	0.6259	H → L (98%)	2.57
	322	348	0.1996	H-2 → L (73%)	
**Py-CN**	383	378	0.9339	H → L (98%)	2.93
	315	324	0.3024	H-2 → L (50%)	
**Py-CF$_3$**	382	372	0.8947	H → L (98%)	3.00
	310	317	0.3025	H-2 → L (40%)	
**Py-Br**	380	370	0.9625	H → L (98%)	3.08
	311	308	0.2496	H-2 → L (63%)	
**Py-Ph**	382	378	1.1309	H → L (98%)	3.03
	313	331	0.2173	H-2 → L (74%)	
**Py-CH$_3$**	378	368	0.8196	H → L (98%)	3.11
	311	330	0.2201	H-2 → L (67%)	
**Py-PCN**	383	377	0.6758	H → L (98%)	2.99
	355	358	0.5021	H-2 → L (61%)	
	338	342	0.2539	H → L+2 (50%)	
**Py-PPh**	358	387	0.8867	H → L (98%)	3.06
	344	342	0.0302	H-2 → L (56%)	
	332	325	0.0612	H → L+2 (32%)	

7. Photochromism Properties

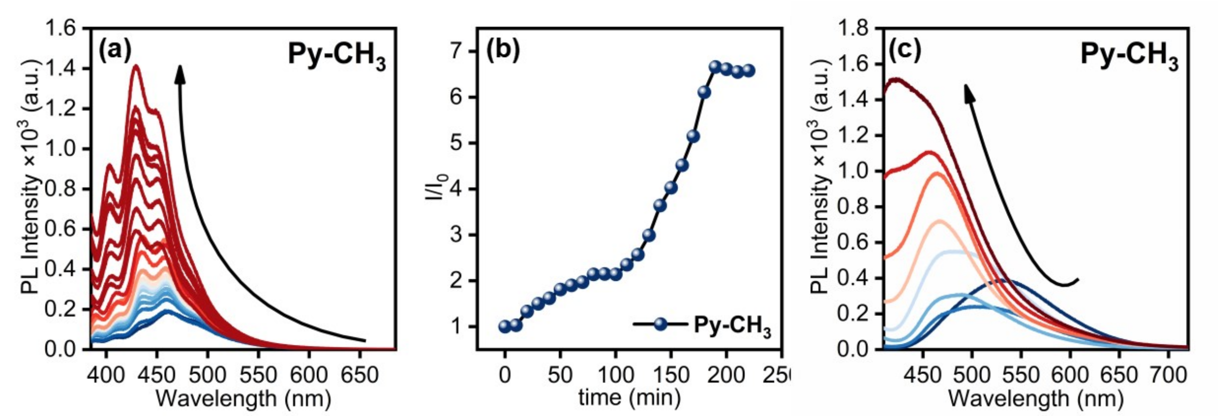

Figure S72. (a) Emission spectra of **Py-CH$_3$** in THF under different irradiation time (3 h); (b) Plot of fluorescence intensity (I/I$_0$) vs. irradiation time in THF; (c) Emission spectra of **Py-CH$_3$** with f_w = 90% under different irradiation time (1 h).

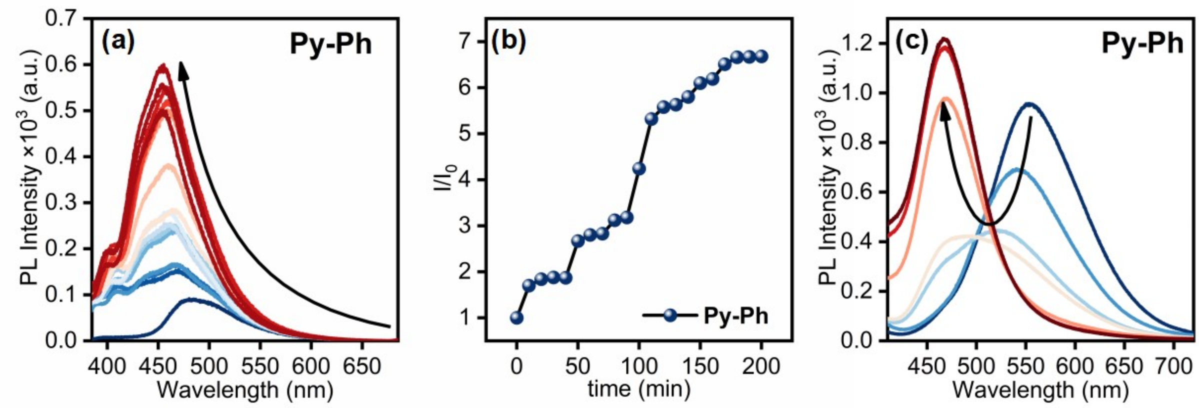

Figure S73. (a) Emission spectra of **Py-Ph** in THF under different irradiation time (3 h); (b) Plot of fluorescence intensity (I/I$_0$) vs. irradiation time in THF; (c) Emission spectra of **Py-Ph** with f_w = 90% under different irradiation time (1 h).

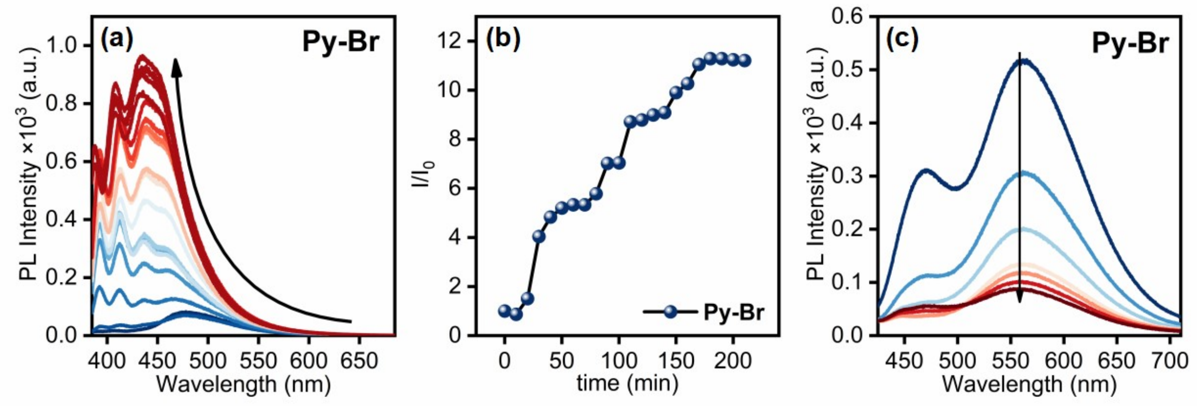

Figure S74. (a) Emission spectra of **Py-Br** in THF under different irradiation time (3 h); (b) Plot of fluorescence intensity (I/I$_0$) vs. irradiation time in THF; (c) Emission spectra of **Py-Br** with f_w = 90% under different irradiation time (1 h).

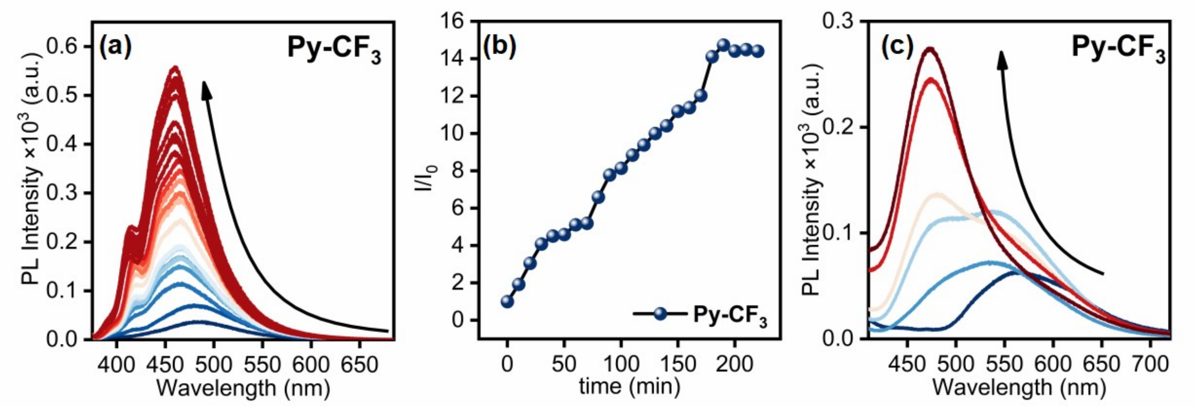

Figure S75. (a) Emission spectra of **Py-CF$_3$** in THF under different irradiation time (3 h); (b) Plot of fluorescence intensity (I/I$_0$) vs. irradiation time in THF; (c) Emission spectra of **Py-CF$_3$** with f_w = 90% under different irradiation time (1 h).

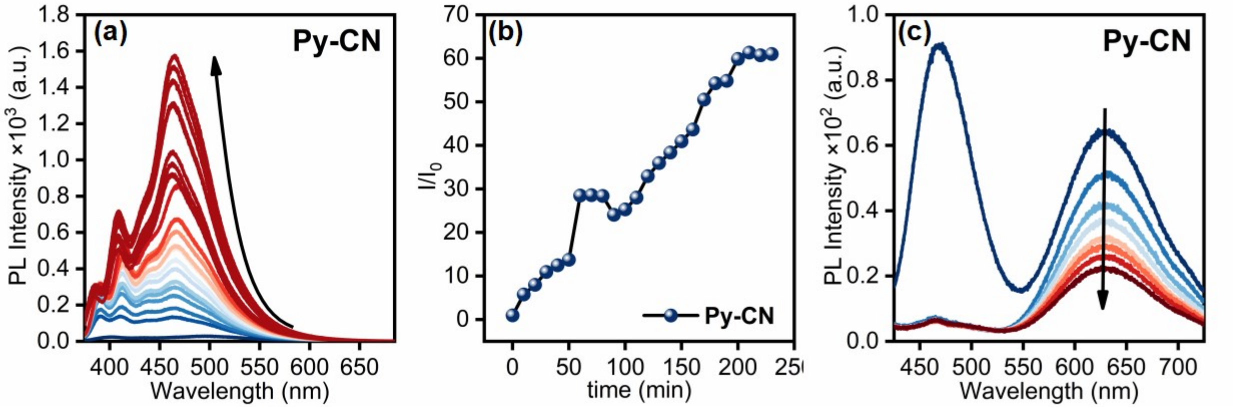

Figure S76. (a) Emission spectra of **Py-CN** in THF under different irradiation time (3 h); (b) Plot of fluorescence intensity (I/I$_0$) vs. irradiation time in THF; (c) Emission spectra of **Py-CN** with f_w = 90% under different irradiation time (1 h).

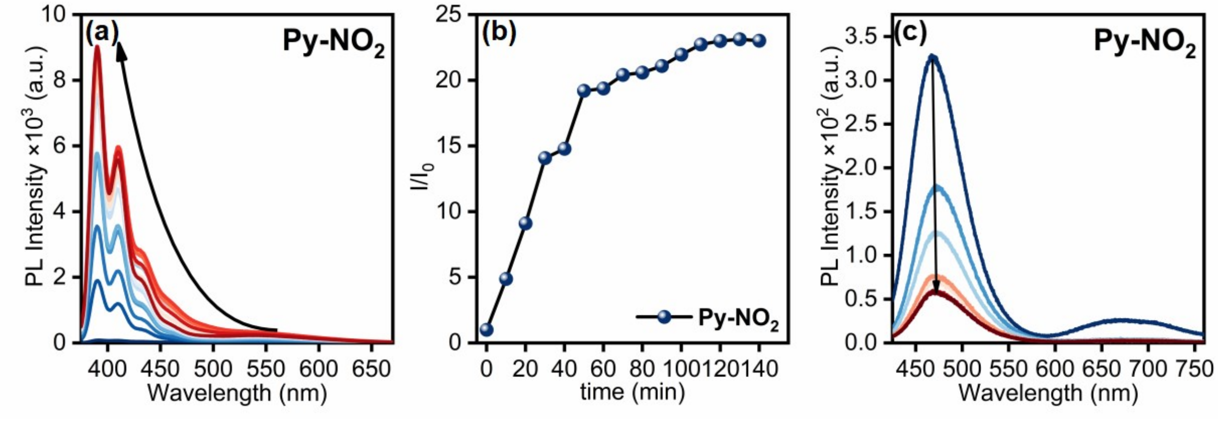

Figure S77. (a) Emission spectra of **Py-NO$_2$** in THF under different irradiation time (3 h); (b) Plot of fluorescence intensity (I/I$_0$) vs. irradiation time in THF; (c) Emission spectra of **Py-NO$_2$** with f_w = 90% under different irradiation time (1 h).

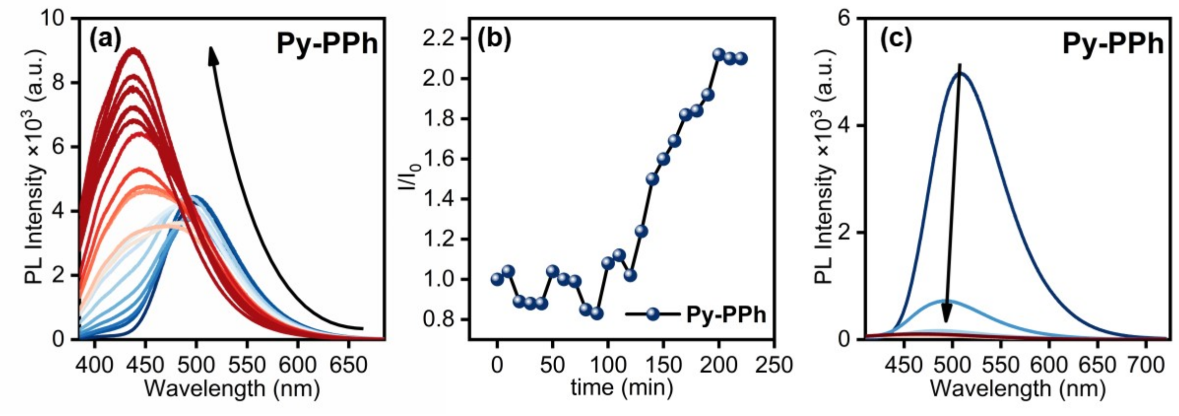

Figure S78. (a) Emission spectra of **Py-PPh** in THF under different irradiation time (3 h); (b) Plot of fluorescence intensity (I/I_0) vs. irradiation time in THF; (c) Emission spectra of **Py-PPh** with f_w = 90% under different irradiation time (1 h).

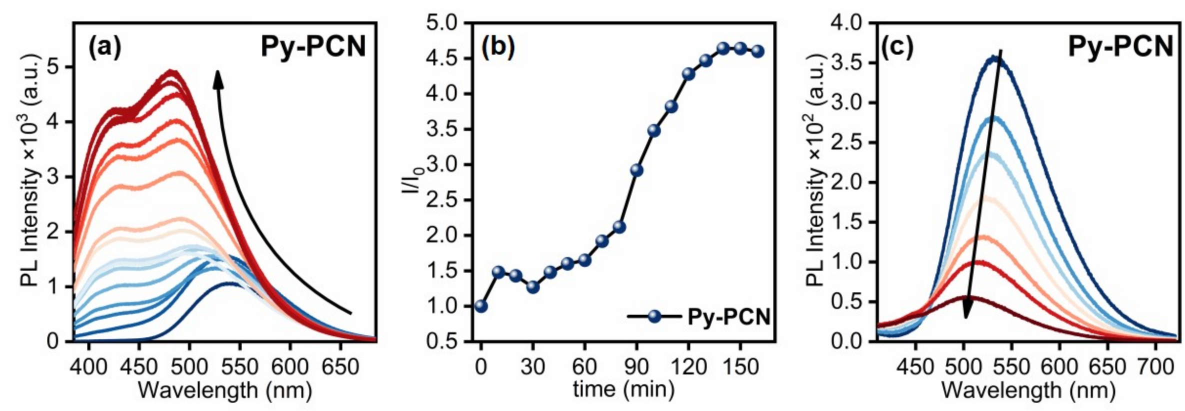

Figure S79. (a) Emission spectra of **Py-PCN** in THF under different irradiation time (3 h); (b) Plot of fluorescence intensity (I/I_0) vs. irradiation time in THF; (c) Emission spectra of **Py-PCN** with f_w = 90% under different irradiation time (1 h).

Table S5. The relative energy barrier in different chemical process

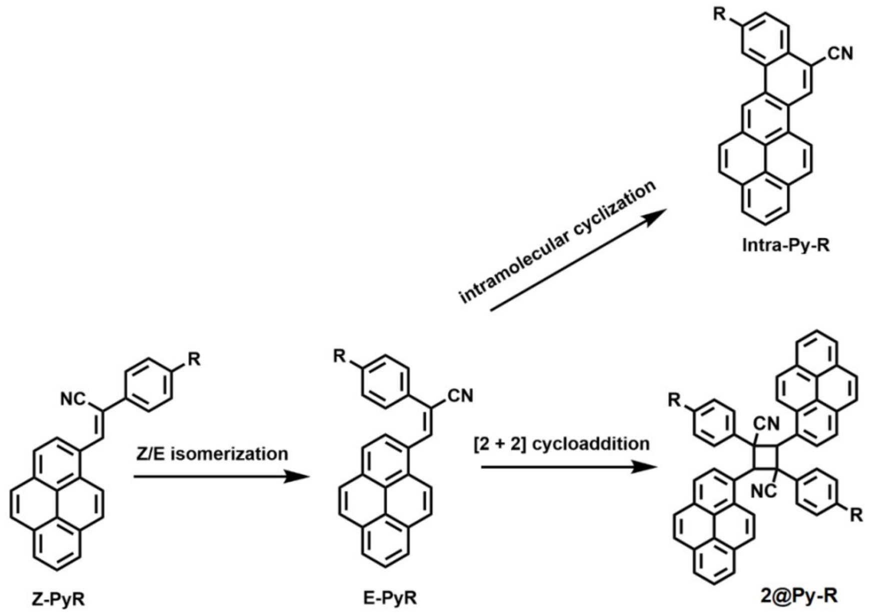

Comps.	Relative energy barrier (kcal/mol)		
	Z*→*E	[2+2] cycloaddition	Intra-cyclization
**Py-CH_3**	1.78	6.52	730.66
**Py-Ph**	1.88	8.78	734.17
**Py-Br**	3.27	3.79	727.76
**Py-CF_3**	1.78	5.95	731.43
**Py-CN**	1.82	11.75	732.28
**Py-NO_2**	1.97	7.51	728.79
**Py-PPh**	2.18	8.00	726.34
**Py-PCN**	3.16	8.56	730.08

Table S6. The HOMO, LUMO levels and energy gap of **Py-R** and **Py-PR** in different chemical process

Comps.	HOMO (eV)			LUMO (eV)			Energy gap (eV)		
	***Z*-**	***E*-**	**2@Py-R**	***Z*-**	***E*-**	**2@Py-R**	***Z*-**	***E*-**	**2@Py-R**
**Py-CH_3**	-5.66	-5.56	-5.51	-2.55	-2.35	-1.90	3.11	3.21	3.61
**Py-Ph**	-5.68	-5.76	-5.58	-2.65	-2.57	-1.89	3.03	3.19	3.59
**Py-Br**	-5.82	-5.69	-5.55	-2.74	-2.01	-1.87	3.08	3.26	3.68
**Py-CF_3**	-5.79	-5.78	-5.70	-2.79	-2.48	-1.95	3.00	3.30	3.75
**Py-CN**	-5.82	-5.83	-5.65	-2.89	-2.59	-2.06	2.93	3.24	3.59
**Py-NO_2**	-5.89	-5.88	-5.80	-3.28	-3.06	-2.97	2.57	2.82	2.83
**Py-PPh**	-5.62	-5.52	-5.37	-2.56	-2.36	-1.74	3.06	3.16	3.63
**Py-PCN**	-5.77	-5.63	-5.54	-2.78	-2.39	-1.91	2.99	3.24	3.63
